# Quantification of the physiochemical constraints on the export of spider silk proteins by *Salmonella *type III secretion

**DOI:** 10.1186/1475-2859-9-78

**Published:** 2010-10-25

**Authors:** Daniel M Widmaier, Christopher A Voigt

**Affiliations:** 1 Chemistry and Chemical Biology Graduate Program, University of California - San Francisco, San Francisco, CA, 94158, USA; 2 Department of Pharmaceutical Chemistry, University of California - San Francisco, San Francisco, California, 94158, USA

## Abstract

**Background:**

The type III secretion system (T3SS) is a molecular machine in gram negative bacteria that exports proteins through both membranes to the extracellular environment. It has been previously demonstrated that the T3SS encoded in *Salmonella *Pathogenicity Island 1 (SPI-1) can be harnessed to export recombinant proteins. Here, we demonstrate the secretion of a variety of unfolded spider silk proteins and use these data to quantify the constraints of this system with respect to the export of recombinant protein.

**Results:**

To test how the timing and level of protein expression affects secretion, we designed a hybrid promoter that combines an IPTG-inducible system with a natural genetic circuit that controls effector expression in *Salmonella *(*psicA*). LacO operators are placed in various locations in the *psicA *promoter and the optimal induction occurs when a single operator is placed at the +5nt (234-fold) and a lower basal level of expression is achieved when a second operator is placed at -63nt to take advantage of DNA looping. Using this tool, we find that the secretion efficiency (protein secreted divided by total expressed) is constant as a function of total expressed. We also demonstrate that the secretion flux peaks at 8 hours. We then use whole gene DNA synthesis to construct codon optimized spider silk genes for full-length (3129 amino acids) *Latrodectus hesperus *dragline silk, *Bombyx mori *cocoon silk, and *Nephila clavipes *flagelliform silk and PCR is used to create eight truncations of these genes. These proteins are all unfolded polypeptides and they encompass a variety of length, charge, and amino acid compositions. We find those proteins fewer than 550 amino acids reliably secrete and the probability declines significantly after ~700 amino acids. There also is a charge optimum at -2.4, and secretion efficiency declines for very positively or negatively charged proteins. There is no significant correlation with hydrophobicity.

**Conclusions:**

We show that the natural system encoded in SPI-1 only produces high titers of secreted protein for 4-8 hours when the natural *psicA *promoter is used to drive expression. Secretion efficiency can be high, but declines for charged or large sequences. A quantitative characterization of these constraints will facilitate the effective use and engineering of this system.

## Background

**Figure 1:**
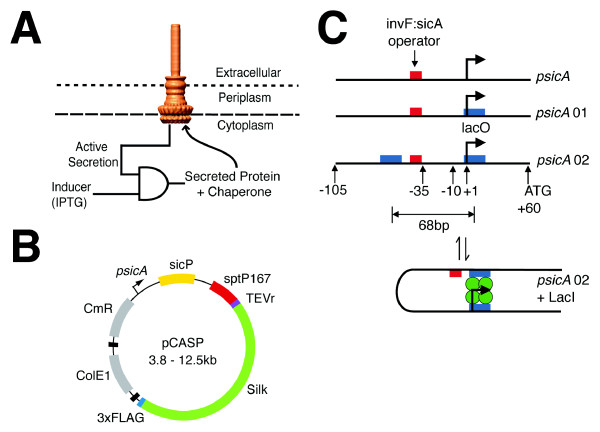
**An overview of the system is shown**. **A**. The protein to be secreted is controlled by a genetic AND gate that is responsive to a genetic circuit that responds to *psicA* inducer added to the culture media (IPTG). The needle image is from reference [48]. **B**. A map of the pCASP plasmid is shown. The black features represent transcriptional terminators. **C**. Three promoters used in this study are shown with critical features marked. The red box represents the invF:sicA operator binding site [14]. The blue boxes represent the LacO1 operator-binding site. Various spacings of Lac operators are generated and tested here to find optimal separation for LacO looping by the LacI tetramer (green circles).

Protein secretion is a useful tool for applications in biotechnology when proteins need to be exported for their function or to ease purification [1]. Gram negative bacteria have an inner and outer membrane, both of which need to be crossed for proteins to be exported to the extracellular environment. The type III secretion system (T3SS) has this capability, where it forms a large molecular machine that crosses both membranes (Figure 1) [2], [3-5]. In its natural context, the T3SS is a common virulence mechanism for pathogenic bacteria to deliver proteins to host cells during pathogenesis. T3SS's from various organisms have been harnessed to export recombinant proteins, including enzymes, peptides to induce an immune response, and spider silk proteins [4-11]. Little is known as to the range of foreign proteins that can be exported and any limits to their properties.

A T3SS is encoded in *Salmonella *Pathogenicity Island 1 (SPI-1), which is a 34 kilobase gene cluster [12]. Proteins secreted by the T3SS contain an N-terminal peptide tag and a chaperone binding domain [13]. The SptP tag contains 167 amino acids, including a 35 amino acid secretion signal and 104 amino acid chaperone binding domain [13]. SicP is the cognate chaperone and is required for secretion. SPI-1 contains a genetic circuit that links effector expression with the completed assembly of the needles [14,15]. This circuit consists of a transcription factor (InvF) that is activated by a chaperone (SicA) that is sequestered by effector proteins prior to the needle being completed. The InvF:SicA_2 _complex upregulates effector expression by inducing the *psicA *promoter. Previously, we created a plasmid system (pCASP) to export recombinant proteins, where *psicA *drives the expression of the SicP chaperone and the first 167 amino acids of SptP fused to the recombinant protein to be secreted (Figure 1) [6]. The tag can be removed post-secretion by using the TEV protease.

There are several outstanding questions regarding the suitability of using the SPI-1 T3SS for industrial applications. First, it is controlled by a complex regulatory network that ensures it is expressed at the correct time and location [16]. When induced in culture, SPI-1 genes are strongly expressed for ~6 hours before being repressed [15,17]. This could be related to its role in pathogenesis, where the SPI-1 T3SS is transiently expressed to inject effector proteins into mammalian cells to facilitate invasion [2]. After invasion, SPI-1 is strongly repressed and a second T3SS encoded within SPI-2 is expressed to promote intracellular survival [2]. Second, it is unclear how the overexpression of recombinant protein from a medium-high copy plasmid affects the efficiency of secretion. Finally, it has been observed that some recombinant proteins will secrete whereas others will not, but the physiochemical properties of peptides that favor secretion have not been well characterized.

The field of synthetic biology has yielded a number of genetic circuits that implement dynamic and logical operations [18]. Elowitz and co-workers created a library of multiple-input logic gates by inserting the transcription factor binding sites randomly into three locations: 1. upstream of the -35 site, 2. between the -35 and -10 sites, and 3. after the transcription start site [19]. They identified promoters that behave as OR- and AND- gates, with respect to the transcription factor inputs. In this paper, we apply this approach to create a hybrid genetic circuit that enables the characterization of how secretion is affected by the timing and magnitude of expression. This is based on regulatory components from the *E. coli *lactose utilization operon [20]. A 2-input AND gate is constructed by inserting lacI binding sites into the *psicA *promoter. This promoter is only active when the secretion needle is functional and the inducer IPTG is present (Figure 1). Further, by including upstream binding sites, looping induced by LacI tetramers can produce tighter control of basal expression [21-25]. We use these circuits to induce the expression at different timepoints and magnitudes, while maintaining the dynamics of the SPI-1 regulatory network, in order to measure how these parameters impact secretion.

Spider silk proteins are natural polymers with physical properties that exceed the most sophisticated petroleum-based polymers. Natural silks represent a variety of length, charge, and amino acid compositions that differ significantly from folded proteins. In previous work, we demonstrated the pCASP system by secreting three silk proteins, including web framing (ADF-1), cocoon (ADF-2), and dragline (ADF-3) silks from the orb-weaving spider *Araneus diadematus *[6]. In this manuscript, we expand the set of silk proteins to include those that are used in flagelliform silk (NCF1), egg sac/cocoon construction (BMF1), and dragline silk (ADF4, LHF1). These represent diverse amino acid compositions and mechanical properties. The flagelliform silk (NCF1) from the orb-weaving spider *Nephila clavipes *has rubber-like properties with the ability to stretch to > 300% of its length before failure. This is due to the presence of proline in the GPGGX motif which forms a 3_10 _helix structure that acts as a series of springs in the final fiber [26]. The *Bombyx mori *(silkworm) silk (BMF1) forms β-sheets composed of (GA)_n _repeats rather than the (A)_n _repeats common in spider silks, which leads to stiffer and less extensible fibers. Because of their long length and highly repetitive sequences, it has proven difficult to DNA sequence full length spider silk proteins, although there has been recent success [27]. Some recent evidence suggests that native length silk protein monomers are critical for obtaining the natural mechanical properties [28]. In this paper, we construct the first fully synthetic DNA construct of the 3129 amino acid full-length major ampullate spidroin 1 gene (LHF1) from *Latrodectus hesperus *(black widow spider) using only sequence information from NCBI [27]. This sequence retains the non-repetitive N- and C- terminal domains, which contain a strong Sec secretion signal and have been proposed to be important for assembly into fibrils [29-31]. The full-length sequence does not express in *Salmonella *and we create a series of truncation mutants to characterize the length dependence on expression and secretion.

While not our original intention, spider silk proteins provide a unique test set for studying the constraints on secretion. Foremost, they are long polypeptides that have no internal structure. This eliminates the effects of protein stability and unfolding kinetics, both of which are critical for a protein to be secreted [5]. They also naturally contain extreme variations in amino acid composition, charge, length, and hydrophobicity. Further, the amino acid sequences are strongly resistant to proteolysis. The calculation of physiochemical properties, such as charge and hydrophobicity, are more accurate for extended peptides than folded proteins.

Work has been done previously to identify the properties of natural effector proteins that make them conducive to secretion. Effectors have highly variable sequences and show no amino acid similarity to each other [32]. Different computational approaches have been developed to search for features that distinguish effectors from non-secreted proteins [33]. These have been mostly applied to understanding the composition and structure of the N-terminal sequence in order to identify secretion signals. It has also been noted that throughout the entire protein, serine and asparagine are enriched, while phenylalanine and arginine are reduced [34]. Structural algorithms have predicted that effectors proteins are more disordered than non-secreted proteins [35].

## Results and Discussion

### Addition of LacI binding sites to the SicA promoter

SPI-1 contains an internal promoter that controls the expression of effector proteins that are exported from the cell. The sicA promoter is controlled by a genetic circuit that induces the upregulation of effector proteins after the needle is functional. This promoter is one of the last to be turned on after SPI-1 is induced [15]. Here, we construct a version of the sicA promoter that can be activated by a small molecule inducer. The goal is to create a 2-input AND gate (Figure 1A), where the promoter is still derepressed by the SPI-1 regulatory network, but it is also controlled by the external inducer. Beyond this project, this approach could provide a general mechanism whereby the environmental inputs and dynamics of a regulatory pathway are preserved, while also enabling external control.

**Figure 2:**
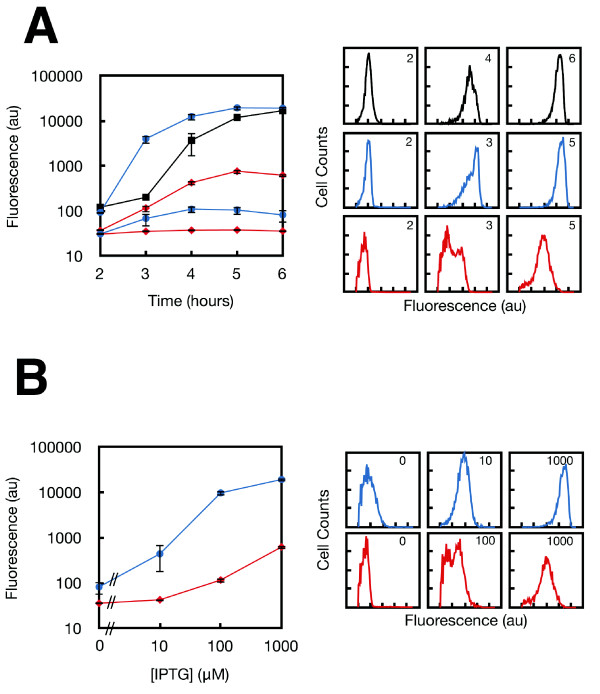
**The responses of the IPTG-inducible *psicA *promoters are shown with respect to time and concentration of inducer**. **A**. The temporal response of the wild-type *psicA *promoter (black) is shown. The dynamics of the *psicA*01 (blue circles) and *psicA*02 (red diamonds) are shown with 0 and 1000 μM IPTG. The timepoints are labeled (in hours) on the cytometry distributions. **B**. The transfer functions of the *psicA*01 and *psicA*02 promoters are shown over a range of IPTG concentrations. The cytometry histograms are shown with the concentration of inducer labeled (in units of μM). Each point shown is an average of three independent colonies and error bars are +/- one standard deviation.

First, a hybrid *psicA *promoter was constructed with a single lac operator binding site that overlaps transcription initiation (*psicA*01) (Figure 1C). The lacI gene is expressed from a second plasmid pREP-4 (p15a, Kan^R^, LacUV5 promoter LacI). Promoter activity is determined using transcriptional fusions between the promoter and green fluorescent protein with fluorescence measured using flow cytometry (Methods). The wild type *psicA *promoter has a 125-fold induction when cells are grown in SPI-1 inducing medium (IM) over a time course of 6 hours (Figure 2A). The wild-type promoter is one of the last promoters in SPI-1 to turn on and it reaches its half-maximal activity 4 hours after induction [15]. In the presence of 1000 μM IPTG, the *psicA*01 promoter has a slightly stronger 200-fold induction and turns on earlier, with the half-activation at 3 hours. The cytometry distributions are similar between wild-type *psicA *and *psicA*01. The *psicA*01 promoter is strongly inducible with a 230-fold change between 0 and 1000 μM IPTG after 6 hours and it is half-activated at 22 μM (Figure 2B). However, the promoter is leaky and shows a 3-fold induction over time in the absence of IPTG.

A second lacI binding site was inserted upstream to reduce the induction in the absence of IPTG. The natural lac operon contains multiple binding sites that cause DNA looping when lacI tetramers are bound. When looping occurs, the entire complex sterically occludes the binding of RNA polymerase, thus preventing transcription initiation. To harness this effect, upstream O1 operators were placed in the *psicA *promoter to facilitate looping. The strength of repression is dependent on the distance between lac operator sites and this follows a periodicity of 11nt with an optimal distance of 71nt [25]. This periodicity occurs due to the energy penalty required to align operators on the same side of the DNA [36]. Following the periodicity suggested by Müller and co-workers, we made variants with operator sites inserted 71, 82, and 93 bp. In addition, a small library was generated that included deviations in the number of nucleotides around these points (68, 69, 70, 71, 72, 74, 90, 91, 92, 94, 95, 96). Each of these 15 promoters was screened for the lowest basal level of expression and the highest induction in the presence of 1000 μM IPTG (Additional File 1, Figure S2). The range of induction in the library was observed to be 1-172 fold with an optimum at 72nt. The promoter with 68nt separation between operators (*psicA*02) was chosen because it has extremely tight repression in the absence of IPTG and can be induced 27-fold.

The *psicA*02 promoter has a 2.9-fold lower basal level of activity when compared to the promoter containing a single operator (*psicA01*) (Figure 2A). Under SPI-1 inducing conditions, *psicA*02 shows 17-fold induction (Figure 2B). However, it is only slightly induced during the time course in the absence of IPTG. The half-activation levels are delayed both in inducer concentration (100 μM) and time (3.5 hours) when compared with the single operator construct. At intermediate levels of induction for both time (3 hours) and IPTG (100 μM), the cytometry data is bimodal (Figure 2). Thus, the *psicA*01 and *psicA*02 promoters enable expression to be controlled over a wide dynamic range while maintaining the timing encoded in the SPI-1 regulatory network.

### Temporal Dynamics of Secretion from the SPI-1 T3SS

The *psicA*01 and *psicA*02 promoters enable inducible expression while maintaining the temporal dynamics encoded in the SPI-1 regulatory network. Previous work indicated that the SPI-1 T3SS is induced for six hours in culture [17]. While the expression of the structural genes turn off, secretion could continue until the needles are diluted by cell division. To test the temporal dynamics of recombinant protein secretion, *psicA*02 was cloned into pCASP plasmid (Figure 1B) and was used to drive the expression of a 294 amino acid fragment of spider silk ADF-2. This protein was chosen because it demonstrates the highest titers (88 μmol/L-hr) and secretion efficiency (14.5%) of the silk monomers tested [6]. The plasmid containing *psicA*02/ADF-2 is co-transformed with the pREP-4 plasmid, from which the LacI protein is constitutively expressed.

**Figure 3:**
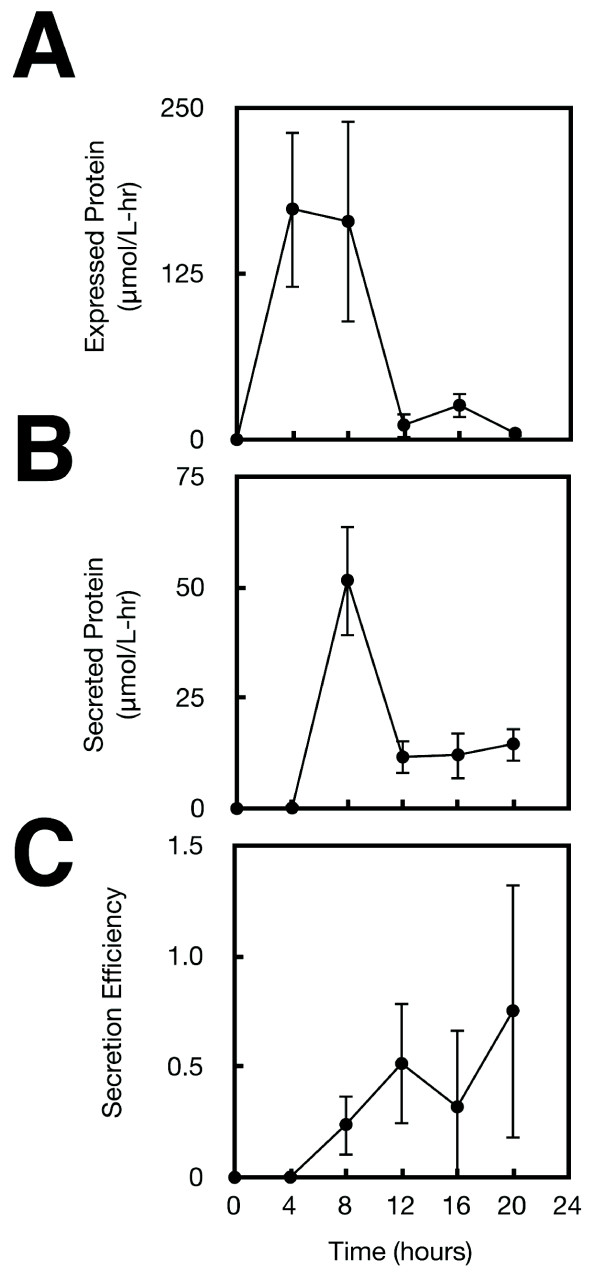
**The production and secretion of ADF-2 is shown as a function of time**. In these experiments, each data point represents the time at which IPTG is added to the media and the titer is the amount of protein observed after 4 hours. **A**. Protein expression initiates by 4 hours and maximum production is achieved between 4-12 hours. Expression rapidly declines after 8 hours, indicating that the *psicA *promoter in inactive. **B**. ADF2 silk protein secretion measured with 4-hour resolution for up to 20 hours. **C**. The secretion efficiency of Type III Secretion, defined as secreted ADF2 protein divided by total expressed, over 20 hours. Each point is the average of three independent colonies and error bars represent +/- one standard deviation.

Experiments were performed where cells are grown in SPI-1 inducing conditions and inducer is added at different time points. For each time point, an aliquot is collected 4 hours after adding 1000 μM IPTG and the amount of ADF-2 is determined in the supernatant and cytoplasm (Methods). The data for these experiments are shown in Figure 3. Each point in this figure represents the amount of protein collected after the 4-hour induction; for example, the point at 16 hours indicates that inducer was added at 12 hours and the sample was collected at 16 hours. The full time course spans 20 hours.

There is no expression when inducer is added at 0 hours, the point at which the cells are are shifted to SPI-1 inducing conditions (Figure 3A). This is consistent with the lack of activity from *psicA *before the secretion system is constructed [14,15]. When inducer is added at 0 hours, expression reaches its maximum level by 4 hours. However, no secretion is observed during this period (Figure 3B). Secretion peaks when inducer is added at 4 hours and then falls 10-fold and plateaus for the remainder of the time course. This indicates that there is only a short period where high secretion fluxes are maintained during a 20 hour induction.

Secretion efficiency is defined as the ratio of secreted protein collected from the supernatent to total protein expressed (supernatant + lysate). While the total protein that is expressed and secreted shows a pulse, the secretion efficiency increases steadily over the full time course (Figure 3C). Surprisingly, most of the protein that is expressed is secreted at late time points. Expression is significantly lower during this period and the relationship between secretion efficiency and expression level is explored in the next section. This data implies that, while secretion complexes are able to continue protein export, the *psicA *promoter is significantly less active in stationary phase.

### Protein Expression versus Secretion

The *psicA *hybrid promoters driving the secretion of ADF2 are used to determine the relationship between protein expression and secretion. It is possible that there is an optimum expression level for maximizing secretion efficiency, where high levels of expression could saturate secretion. The two IPTG-inducible sicA promoters (*psicA*01 and *psicA*02) enable a wide range of expression levels to be tested, while maintaining the same dynamics for each concentration of inducer. As above, the expression and secretion of ADF2 was measured.

**Figure 4:**
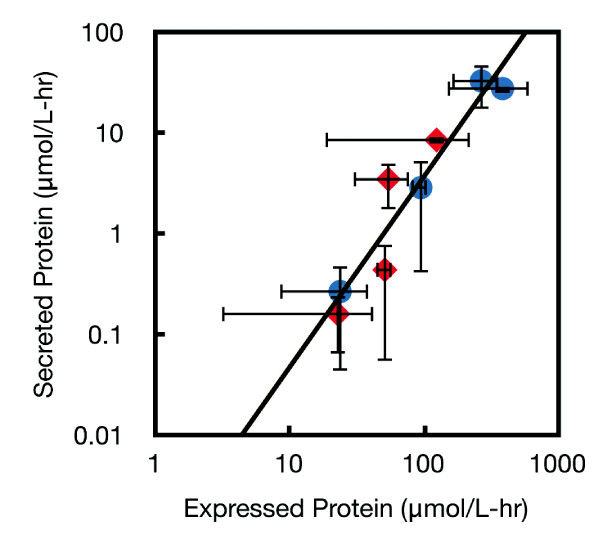
**Expression and secretion are measured for ADF2 silk protein under the control of *psicA*01 (blue circles) and *psicA*02 (red diamonds)**. A power law fit of the data is overlaid with a fitting equation of y = 0.0006×^1.8921^. Each point is the average of three independent colonies and error bars represent +/- one standard deviation.

A series of inducer (IPTG) titration experiments were performed with *psicA*01 and *psicA*02 to measure ADF2 in the cell lysate and in the culture supernatant after a 6-hour induction (Figure 4 and Additional File 1, Figure S3). IPTG was added to 0, 10, 100, and 1000 μM concentrations and the ADF2 concentration was measured using quantitative western blotting (Methods). The *psicA*01 promoter follows a monotonically increasing trend with increasing inducer concentration, with a maximum efficiency of 11% at 1000 μM IPTG (Additional File 1, Figure S3C). The *psicA*02 promoter reaches a maximum efficiency of 22% at 1000 μM IPTG. The relationship between expressed and secreted protein produces an average efficiency of 5.8%, which is conserved between the promoters over almost two orders of magnitude of expression. The lack of a plateau at higher expression levels may be due to the moderate expression of ADF-2 even at full induction.

### Secretion of Spider Silk Proteins with Diverse Physiochemical Properties

The spider silk family of proteins encompasses a wide range of amino acid compositions and physiochemical properties, which ultimately result in diverse mechanical properties. Using sequence information from Genbank, the amino acid sequences of several silk genes were used to generate computationally optimized DNA sequences for expression in gram negative bacteria [37]. Each optimized gene was synthesized using automated whole gene synthesis and cloned into the pCASP system (Figure 1B). The silk genes synthesized for this study include the full-length gene encoding *Latrodectus hesperus *MaSp1 (LHF1) as well as fragments incorporating all available sequence information for *Nephila clavipes *flagelliform (NCF1), *Araneus diadematus *MaSp2 (ADF4), and *Bombyx mori *heavy chain fibroin (BMF1) (full sequence information in additional file 1) [27,38-41]. The synthetic genes encode the natural amino acid sequence while reducing repetitiveness in the DNA sequence to prevent homologous recombination.

**Figure 5:**
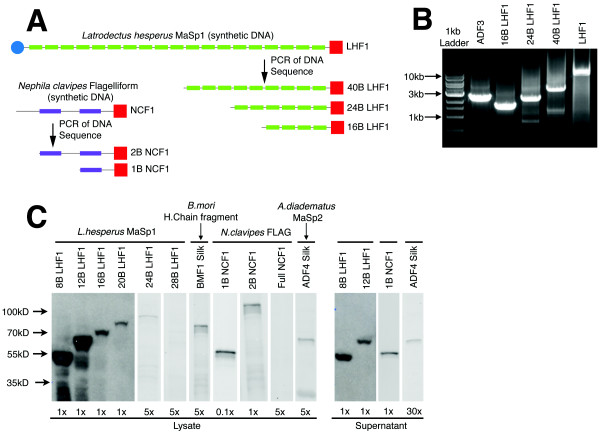
**The construction and testing of many size variants for different silk types**. Silk DNA sequence information was obtained from Genbank for multiple silk genes including *Latrodectus hesperus *Major Ampullate Spidroin 1 (LHF1), *Nephila clavipes *Flagelliform silk (NCF1), *Araneus diadematus *Major Ampullate Spidroin 2 (ADF4), and *Bombyx mori *Heavy Chain Fibroin (BMF1), all of which were optimized and synthesized using chemical gene synthesis. **A**. Long constructs such as full length LHF1 and the partial clone of NCF1 were further divided using PCR to create libraries of truncation mutants. Mutants include (protein: # amino acids): 8B: 329, 12B: 446, 16B: 568, 20B: 685, 24B: 802, 28B: 921, 32B: 1043, 36B: 1165, 40B: 1286, 1B: 252, 2B: 672. **B**. A DNA gel showing PCR products for various truncations. Representative LHF1 truncations show a number of size variants up to full length. A PCR product of the ADF3 gene is included to demonstrate the utility across multiple silk sequences. **C**. Western blots show the detection of silk protein expression and secretion. Each lane is labeled for the concentration of sample loaded and protein identity.

Length variants of LHF1 and NCF1 were constructed by PCR to further expand the library of physical properties tested for SPI-1 secretion (Figure 5A). LHF1 is identified in the literature to have four characteristic amino acid block repeat sequences which repeat in a regular order throughout the protein [27]. The full-length synthetic LHF1 construct was amplified into fragments using polymerase chain reaction (Figure 5B) to give products ranging from 40 blocks to 8 blocks in increments of 4 blocks. Each construct contains the C-terminal hydrophilic domain. The LHF1 truncation constructs range from 330 to 1250 amino acids. A similar process was carried out with NCF1; however, there are fewer block repeats in this silk type and only two truncation mutants were made.

**Figure 6:**
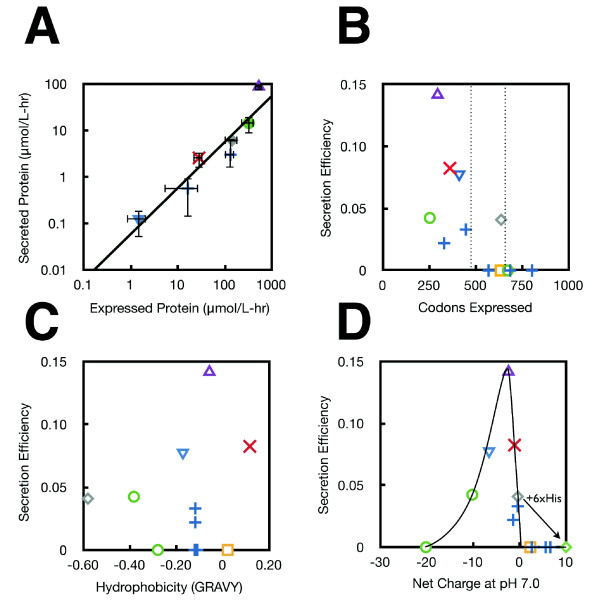
**The properties of secreted proteins**. Silk proteins measured in a previous publication are also plotted for reference [6]. Different spider species are shown by different symbols *Latrodectus hesperus *MaSp1 (blue +), *Nephila clavipes *Flagelliform (green circles), *Bombyx mori *cocoon silk (yellow square), *Araneus diadematus *ADF1 (red X) ADF2 (purple triangle) ADF3 (grey diamonds) and ADF4 (blue triangle). **A**. Secretion of each protein is plotted versus the expression level. Each point is the average of three independent colonies and error bars represent +/- one standard deviation. **B**. Secretion efficiency is plotted as a function of protein length. Parallel dotted lines are shown to guide the eye. The number of amino acids noted only corresponds to the length of the silk polypeptide and does not include the 178 amino acid SptP tag and TEV site. **C**. Secretion efficiency is plotted against hydrophobicity (GRAVY score). **D**. The secretion efficiency versus net charge for each secreted protein is determined at pH 7.0 and plotted. A guide for the eye is drawn over the data. For each silk protein, the charge was calculated including the SptP tag, TEV linker sequence, and 3× FLAG tag. The ADF3 protein has a native charge of -0.4 (grey diamond) and when a 6× histadine tag is added the net charge at pH 7.0 increases to +10 (green diamond). This modification to ADF3 eliminates secretion (identified by the arrow).

The library of silk genes was cloned into the pCASP vector and secretion assays run to determine secretion and expression titers. Samples of supernatant protein and lysate were measured by FLAG epitope tag and quantitative western blot (Figure 5C). The full gels are shown in additional file 1. While many of the silk proteins express, only a few secrete, including truncations of LHF1 and NCF1, as well as ADF-4. The data is combined with that presented previously (ADF1, ADF2, ADF3) to plot the amount of protein expressed versus secreted for a variety of silk proteins [6]. As with the induction of ADF2 (previous section), secretion efficiency is remarkably constant as a function of the expression level (Figure 6A). Of the proteins, ADF2 is an outlier and has a secretion level higher than would be expected. ADF-4 poorly expresses and is at the lower limit for the detection of secretion. A number of silks that express show no evidence for secretion, including 16B, 20B, 24B, BMF1, and 2B. The 28B, 32B, 36B, 40B, NCF1, and LHF1 proteins did not show any detectable expression. The LHF1 truncation sequences show secretion up to 12 repeat units, expression (with no secretion) up to 24 repeat units, and no expression from 28 to 95 repeat units.

For each silk that expressed, the secretion efficiency is calculated as the amount of protein secreted over the total expressed (Figure 6B). A trend is seen where the secretion efficiency declines for larger proteins. All proteins less than 450 amino acids that expressed also secreted, while only a fraction of the proteins up to 685 amino acids secreted, and no proteins over 685 amino acids secreted. Including the 178 amino acid SptP tag and TEV site, these thresholds are 628 and 863 amino acids, respectively. As a comparison, the size of known SPI-1 effectors are: SopE2 (240), AvrA (302), SopD (317), InvJ (336), SipD (343), SipC (409), SptP (543), SopB (561), SipB (593), SipA (685), and SopA (782). It may be that the threshold that we observe was a general limitation in the length of polypeptide that can be translocated through the T3SS.

The secretion efficiency was also compared to the hydrophobicity of the polypeptide. Silks have hydrophilic and hydrophobic domains that are responsible for assembly and the materials properties. There is diversity in the hydrophobicity of the silk proteins that we synthesized. For each protein, the GRAVY score was calculated, which captures the average hydrophobicity of the polypeptide based on the sum of the hydrophobicity of each amino acid [42]. There is no correlation between the GRAVY score and secretion efficiency (Figure 6C). The length of the hydrophobic regions also varies between silk proteins and we looked for correlations between the longest stretch of hydrophobic residues and the frequency of hydrophobic regions, but no correlation was found (not shown).

For each silk protein, the net charge at pH7 was calculated (Methods). In the case of globular proteins, this poorly reflects the total charge of the protein in the folded environment because the pKa's of amino acids will be influenced by the local protein environment. The silk proteins are unfolded and thus, this calculation is more accurate. When the secretion efficiency is plotted against charge, there is an optimum at ADF-2, which has a charge of -2.4 (Figure 6D). The efficiency declines rapidly for positively charged polypeptides and more slowly for negatively charged ones. Interestingly, we observed that the addition of a 3×His tag to ADF-3 (for the purposes of protein purification) eliminated secretion without affecting expression. In contrast, the more negatively charged 3×FLAG tag does not appear to cause a decline in secretion.

## Conclusions

In this manuscript, we have used a set of spider silk polypeptides to characterize the physiochemical properties that limit the use of the SPI-1 T3SS for the export of recombinant proteins. The most significant constraints are on protein length and net charge. Surprisingly, there was little correlation with hydrophobicity and the needle is able to export polypeptides with diverse and unusual amino acid contents. A synthetic circuit is used to characterize the impact of the magnitude and timing of gene expression. The secretion efficiency is constant with respect to expression level. However, the maximum secretion flux only occurs for a relatively short time when grown in culture. Accurate knowledge of the constraints of SPI-1 secretion is critical for selecting appropriate applications and guide the future optimization of the system.

## Methods

### Microorganisms and Media

*Salmonella typhimurium *SL1344 was used for all secretion and fluorescence experiments. All cloning was conducted using TOP10 *E. coli *(Invitrogen, #C664-11). Three medias were used: LB Miller (Difco, #244510), L Broth (Difco, #241210) and SPI-1 Inducing Media (IM) (LB, 0.3 M NaCl [43]). Media was supplemented with 25 μg/mL Kan, 30 μg/mL Cm or 100 μg/mL Amp as needed.

### Construction of IPTG-inducible *sicA Promoter*

The LacI protein is constitutively expressed on a second plasmid (p15a, Kan^R^) (partsregistry.org, pSB3K3 or pREP-4, Qiagen Cat#: 34210). Three constitutive promoters of different strengths were used to titrate the level of LacI protein in the cell. Promoters used include: J23150 [44], lacUV5, and lacIq. Promoters were cloned in front of the lacI open reading frame using inverse PCR mutagenesis and compared to the commercial pREP-4 plasmid. Plasmid maps of these three plasmids can be found in Additional File 1, Figure S4. After screening by GFP reporter assay, pREP-4 was determined to give the best repression and was used for most measurements made in this study. Experiments involving *psicA*01 use the J23150 plasmid variant.

A library of hybrid *psicA *promoters containing one or two lac operator binding sites was constructed. The *psicA *promoter was cloned from the *Salmonella *genome as previously described [6]. The first lac operator O1 [25] was introduced 3' to the -10 hexamer of the promoter (Additional File 1, Figure S1). The O1 palindrome was generated by asymmetric oligonucleotide primers designed to incorporate the 20 bp operator while deleting 20 bp of natural *psicA *DNA. Incorporation was done by inverse PCR using the TaKaRa DNA polymerase kit (Clontech Laboratories #TAKRR002M). Untemplated 3' TA nucleotide overhangs were removed using T4 DNA polymerase (New England Biosciences, #M0203S) to generate blunt ends. Linear blunt ended DNA was ligated using T4 DNA ligase (Invitrogen, #15224-017) in the rapid ligation reaction according to the manufacturer's protocol.

A second O1 operator binding site was introduced with spacings of 71, 82, and 93 bp 5' of the first lac operator using the same method (Additional File 1, Figure S1). For the 71 and 93 bp spacings, additional promoter mutants were generated using the described PCR protocol to insert or delete up to 3 bp at single base resolution immediately 5' to the InvF:SicA_2 _operator binding site. Selected promoters *psicA*01 and *psicA*02 were transferred into the pCASP plasmid (Figure 1B) using the method of Gibson for scarless cloning [45]

### Fluorescence Measurement of Promoter Activity

Cultures were grown as described in the secretion assay (below). Promoter activity was measured by direct fusion of a hybrid *psicA *promoter to green fluorescent protein [15]. In hourly intervals from 2-6 hours after dilution into IM media, the OD_600 _of the cultures was measured in a Cary 50 UV-Vis spectrophotometer (Varian) and a 500 μL aliquot of cells were collected for cytometry analysis. Cells were centrifuged at 5000 rpm for 3 minutes at room temperature in an Eppendorf 5415D micro centrifuge. The media was vacuum aspirated from each sample without disturbing the cell pellet. The pellet was resuspended in 300 μL of ice cold phosphate buffered saline supplemented with 2 mg/mL kanamycin. All samples were stored at 4°C for flow cytometry analysis.

Fluorescence was measured by flow cytometry using a LSR II with HTS attachment in 96-well plate mode. For each sample, 100,000 counts were collected and processed using FlowJo v7.2 (Tree Star, Inc). Samples were gated for forward and side scatter and an axis population on the FITC-A channel was gated out.

### DNA Synthesis and PCR of Silk Genes

The amino acid sequences of spider silk proteins were obtained from the NCBI database (Genbank ascension numbers; LHF1: EF595246, NCF1: AF027973, BMF1: S74439). DNA2.0 (Menlo Park, CA) used the amino acid sequence to backtranslate a DNA sequence that had optimized codon usage for expression in eubacteria using GeneDesigner software [37]. Fully sequenced synthetic DNA constructs were used for the work presented here and the DNA and amino acid sequences can be found in additional file 1.

Silk gene truncations were generated by PCR amplifications of fragments from the DNA of the synthesized silk. Fragments were designed to increase in size based upon the block structure of the silk [27] and the starting point was the 3' end of the construct to match other silk genes used in this study. This was performed by using the KOD polymerase with betaine (EMD4Biosciences Cat#71975) according to the manufacturer's instructions. Fragments were purified by agarose gel extraction using the Zymo DNA Gel Clean extraction kit (Zymo Research Cat#4001) and cloned into the pCASP vector between the HindIII and XbaI sites [6].

### Protein Secretion Assay

The SPI-1 T3SS is induced in IM media and uninduced in L-broth [43]. Cells were plated on L-broth agar plates from frozen stock and grown overnight. Single colonies were picked and grown 13 hours overnight in 5 ml liquid L-broth. The overnights were diluted 1:100 (50 μL) into 5 mL fresh L-broth and grown for 150 minutes at 37°C and 250 rpm. The cultures were diluted a second time 1:10 into 50 mL of inducing media in a non-baffled 250 mL glass flask supplemented with antibiotics and the appropriate IPTG concentration. The data generated in Figures 2, 3, 4 used IPTG supplementation during the dilution to 50 mL. The cultures were grown at 37°C for 6 hours at 160 rpm to correspond to the timing used for the promoter activity assay. The time point T = 0 is immediately after the second dilution step.

Supernatants were harvested by spinning cultures at 3500 g for 30 minutes followed by vacuum filtration through 0.45 μm cellulose acetate filter unit (Corning #430314). The supernatant samples of proteins fused to the SptP N-terminal tag were used for the Western blots without concentration. The ADF4 silk monomers could not be visualized in 1× supernatant, so these samples were concentrated 30× by centrifugal filter (Amicron, #UFC801024).

Cell pellets were collected to measure the amount of protein expressed, but not secreted. The cell pellets from these experiments were washed with 10 mL of PBS (pH 7.4) and pelleted for 30 minutes at 3500 g and resuspended in 10 mL PBS. The cells were lysed by addition of 0.2 mg/mL lysozyme (MP Biomedicals, #100834) and incubated for 15 minutes at room temperature followed by a 30 minute freeze/thaw cycle at -80°C. Finally, the lysate was sonicated for 2 minutes in 1 second pulses with 25% amplitude. The resulting mixture was spun to remove insoluble debris at 3500 g for 20 minutes. A 1 mL sample of the soluble fraction was collected and frozen for quantitative western blot analysis.

### Protein Quantification

The proteins to be detected were engineered to contain a C-terminal 3 × FLAG epitope tag. Samples were prepared in SDS loading buffer under reducing conditions and run on 10% or 12% polyacrylimide gels (Lonza Biosciences). The gels were transferred to PVDF membranes (BioRad, #162-0177) and blocked for 1-8 hours in TBST-1% Bovine Serum Albumin (Sigma, #A3294-50G) (TBS-Tween20) with shaking. The anti-FLAG antibody (Sigma, #F3165) was used as the primary antibody. The anti-FLAG antibody was mixed 1:7,500 in TBST and allowed to bind for 1 hour. After each antibody step, membranes were rinsed three times with deionized water followed by three 10-minute washes in TBST. The secondary antibody (Sheep anti-mouse HRP [Jackson ImmunoResearch, #515-035-003]) was added in TBST at 1:5,000 and allowed to bind for one hour. Development was done using an enhanced chemiluminescent substrate for HRP (Pierce, #32209) and captured on film (Kodak, #178-8207).

Quantitative western blots were run using an identical protocol as above until the secondary antibody step. Here, a Hybond low fluorescence PVDF membrane (Amersham Biosciences, #RPN303LFP) is used. A Cy-5 conjugated secondary antibody (GE Healthsciences, #PA45010) was diluted 1:5,000 in TBST and processed as above. Final washed blots were dried on 8 × 8 × 0.25 inch glass plates for 2 hours or complete dryness. Development was done using a Typhoon 9400 variable mode imager to generate gel images [46,47]. Gel densitometry was done using ImageJ 1.41 (NIH). Each sample was run with duplicate lanes loaded with 10 μL of 1× ADF2 supernatant or 0.1× ADF2 lysate [6] to enable an internal standard of signal across gels. Protein yields were determined using the average of these two bands and was used to determine a single point quantification standard based upon the previously published concentrations of these samples [6]. An exception was made for the full-length LHF1 Lysate gel, where a single standard band was run due to limitations of material.

### Calculation of Physiochemical Properties

Hydrophobicity was calculated by grand average of hydropahticity (GRAVY) score using the calculator application located at http://www.ebioinfogen.com/biotools/protein-gravy.htm. The calculator was run using the default settings and the amino acid sequence for each expressed silk protein and without the SptP amino acid sequence. Charge was calculated using the application located at http://www.scripps.edu/~cdputnam/protcalc.html using the default settings.

## List of Abbreviations

The following abbreviations were used in this work. T3SS: Type III Secretion System; SPI-1: *Salmonella *Pathogeneity Island 1; IPTG: isopropyl β-D-1-thiogalactopyranoside; L: LB Lennox Broth; IM: Inducing Medium (LB Miller 0.3 M NaCl).

## Competing interests

CV declares a competing financial interest. DW declares a competing financial interest.

## Authors' contributions

DW designed and conducted experiments, analyzed data, wrote, revised, and approved the final manuscript. CW supervised experiments, analyzed data, wrote, revised and approved the final manuscript.

## Supplementary Material

Additional file 1 **Supplementary Data and Methods**. Additional raw data, supporting figures, and methods used in this work. Click here for file
