# Supplementary material for: Quantification of the physiochemical constraints on the export of spider silk proteins by Salmonella type III secretion
Source: Microb Cell Fact. 2010 Oct 25;9:78. doi: 10.1186/1475-2859-9-78 (PMC2987917; doi:10.1186/1475-2859-9-78)
Supplement: Additional file 1 — Supplementary Data and Methods. Additional raw data, supporting figures, and methods used in this work. [file 1475-2859-9-78-S1.DOC]

**Supplementary Information**

**Quantification of the physiochemical constraints on the export of spider silk proteins by *Salmonella* type III secretion**

Daniel M Widmaier and Christopher A Voigt

**Table of Contents** **Page**

1. **Construction of Hybrid *psicA* promoters** 2

**Figure S1:** Sequences of hybrid *psicA* promoters with features 3

1. **Measurement of Hybrid Promoter Activity** 3

**Figure S2:** Fluorescent screening of hybrid promoters 4

1. **Secretion and Expression Titer Quantitation** 4

**Figure S3:** ADF2 secretion and expression titration with IPTG 5

1. **LacI Expressing Plasmids** 5

**Figure S4:** Maps for LacI expressing plasmids 6

1. **Quantitative Western Blots** 6

**Figure S5:** Blots for secreted protein IPTG titration 7

**Figure S6:** Blots for secreted protein timecourse 8

**Figure S7:** Blots for silk variant secretion and expression testing 9-10

1. **Silk DNA and Amino Acid Sequences** 11
2. **Supplementary References** 42
3. Construction of Hybrid *psicA* Promoters

The natural *psicA* promoter (Figure S1) was cloned by PCR from *Salmonella typhimurium* SL1344 genomic DNA and is the basis for LacO looping engineering. The lac operator DNA sequence was introduced by site directed mutagenesis (materials and methods) and replaced 20bp of DNA from the wild-type *psicA* promoter. The first variant constructed was *psicA*01 which was further modified to create 71bp, 82bp and 93bp variants based upon optimal spacing found in [1]. The optimal spacing found in this publication was 70.5bp, 81.5bp and 92.5 bp, so a series of single bp spacing mutants were constructed by site directed mutagenesis. Additional bases (up to 3bp) were added or deleted immediately 5’ to the invF:sicA operator site (Figure S1). Interestingly a different optimum spacing for the *psicA* promoter was found compared to Müller et al (Figure S2).


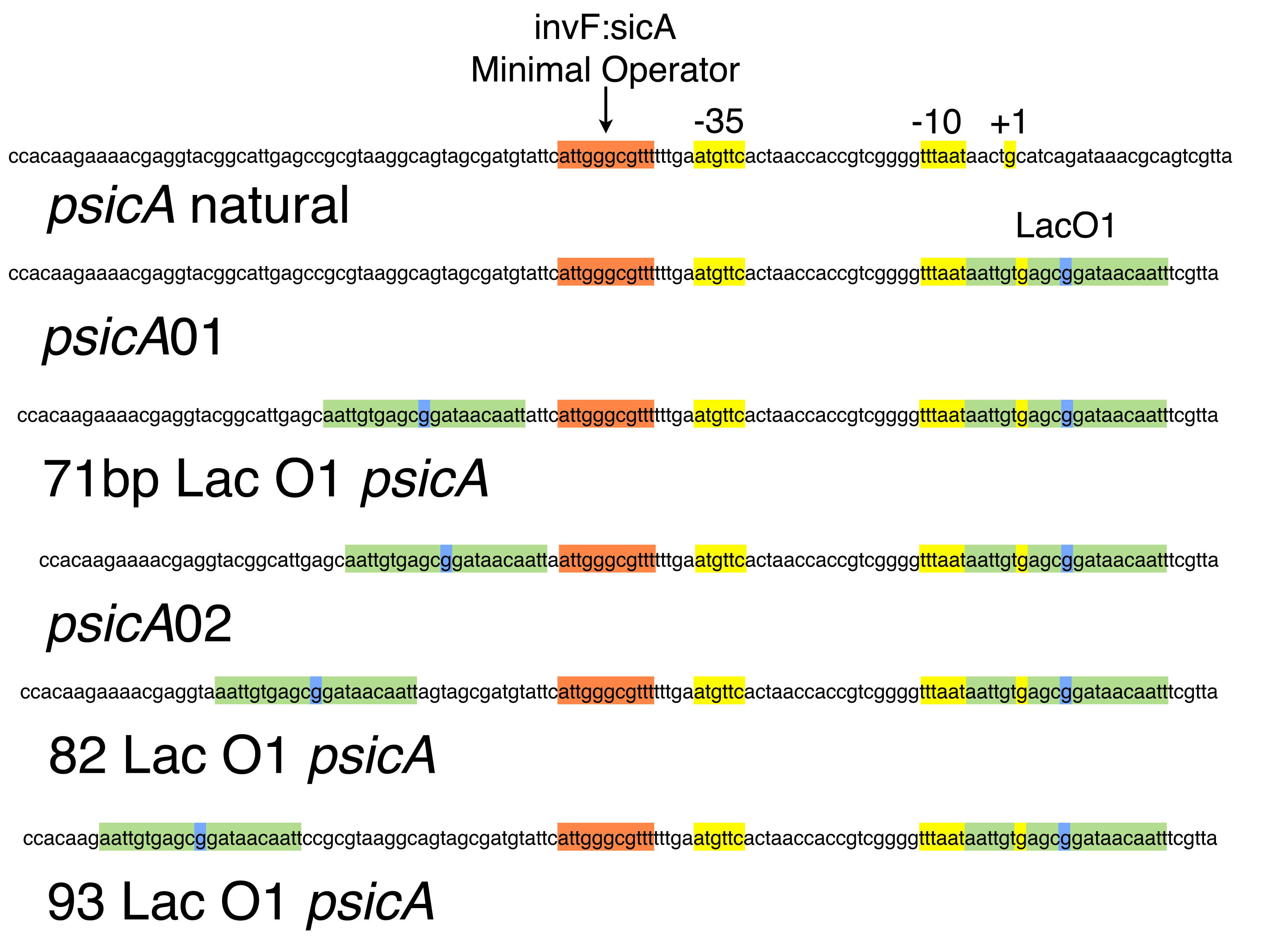


**Figure S1:** The sequence of the natural *psicA* promoter with critical features highlighted; +1, -10, -35 nucleotides (yellow boxes), invF:sicA operator binding site (orange box) [2]. Additional colored boxes (green with blue base) show the placement of the O1 operator DNA binding sites in the sequence to obtain additional system control. Various lac operator spacings were constructed as measured from center to center (blue guanine nucleotide) and are consistent with the literature for enhanced promoter control [1]. Small changes in spacing were achieved by adding or deleting single bases immediately 5’ of the invF:sicA minimal binding box. An example of the spacing changes is shown by comparing *psicA*02, a three base deletion, with 71bp LacO *psicA*.

**2. Measurement of All Hybrid Promoter Function**

The library of LacO spacing mutants were assayed for activity by expressing green fluorescent protein (GFP) in *Salmonella* and measured using flow cytometry (materials and methods). The library was measured twice, once with no IPTG in the culture media and a second time with 1000µM IPTG. The mean fluorescence data is shown in Figure S2A with and without IPTG. The ratio of induced (+ IPTG) and uninduced (-IPTG) is shown in Figure S2B. The sharp repression maxima as a function of operator spacing resemble the pattern found by Müller et al and are an indicator of DNA looping.

**
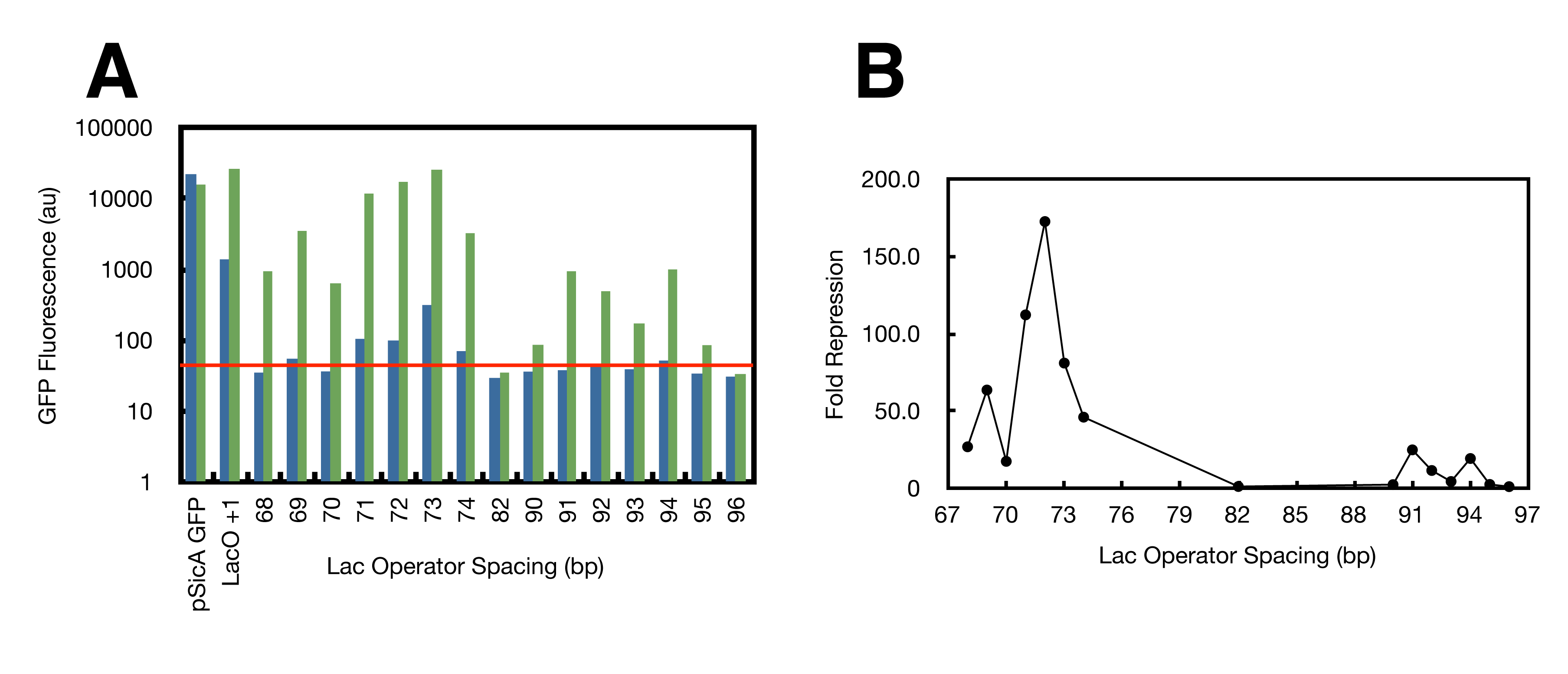
**

**Figure S2:** A measure of dynamic range for a series of *psicA* promoter hybrids. Numerous spacing variants of the *psicA* promoter were created (Figure S1) and tested in the presence or absence of 1000µM IPTG. **A.** All promoters in this study are measured with (green bars) and without IPTG (blue bars) using a GFP reporter and flow cytometry measurements (materials and methods). The mean fluorescence of 100,000 events is shown here. The red line is the average fluorescent value for non-GFP expressing cells (white cells) in the assay. **B.** A plot of the ratio of +/- IPTG vs lac operator spacing.

3. Secretion and Expression Titer Quantitation

The data to generate Figure 4 is a combination of the ADF2 expression and secretion data shown in Figure S3. This data was collected by densitometry analysis of quantitative western blots.
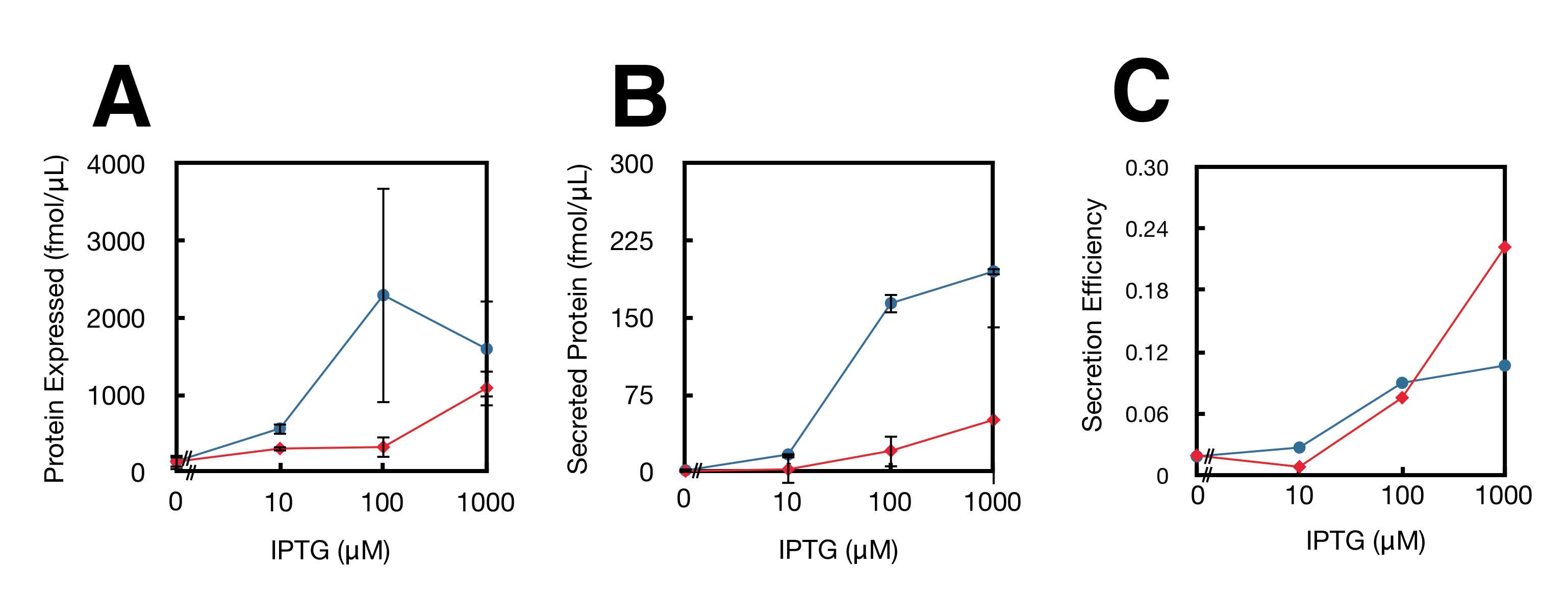


**Figure S3:** Expression **(A)** and secretion **(B)** of ADF2 protein under the control of the *psicA*01 (blue circles) and *psicA*02 (red diamonds) promoters in the presence of various concentrations of inducer (IPTG). Protein titers are measured by quantitative western blotting (materials and methods). Individual gels this data was process from are found in figure **S5**.

**4. LacI Expressing Plamsids**

Several LacI expressing plasmids were used in this study to titrate LacI tetramer concentration in the cell. All plasmids are designed around a core of the LacI open reading frame, Kanamycin resistance marker, and p15a (pACYC) origin of replication. Maps of the specific plasmids with size in kb are provided in figure S4.


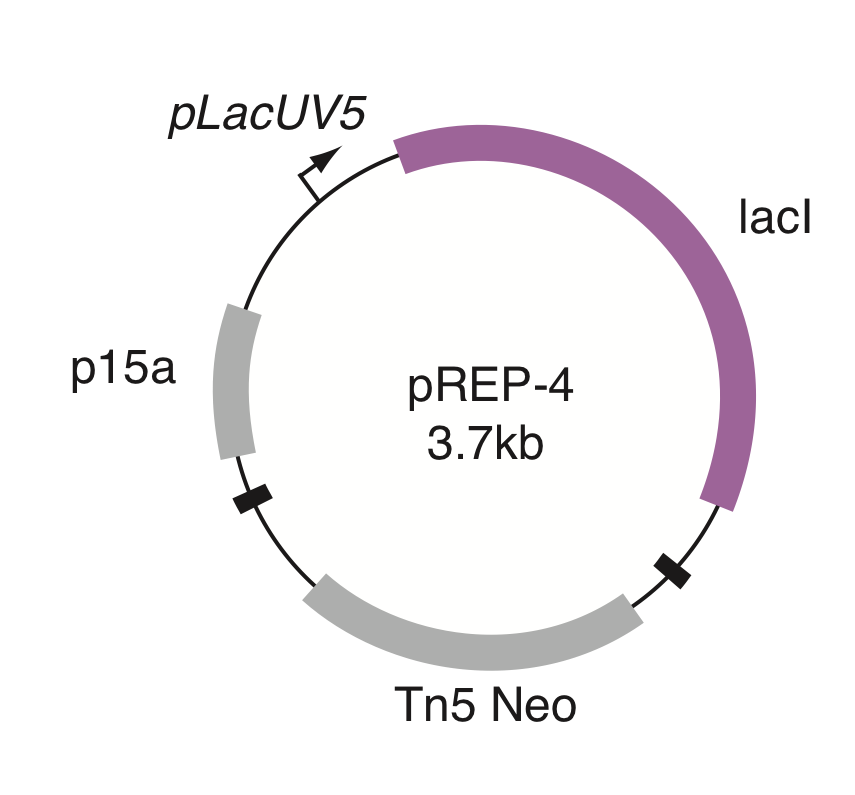

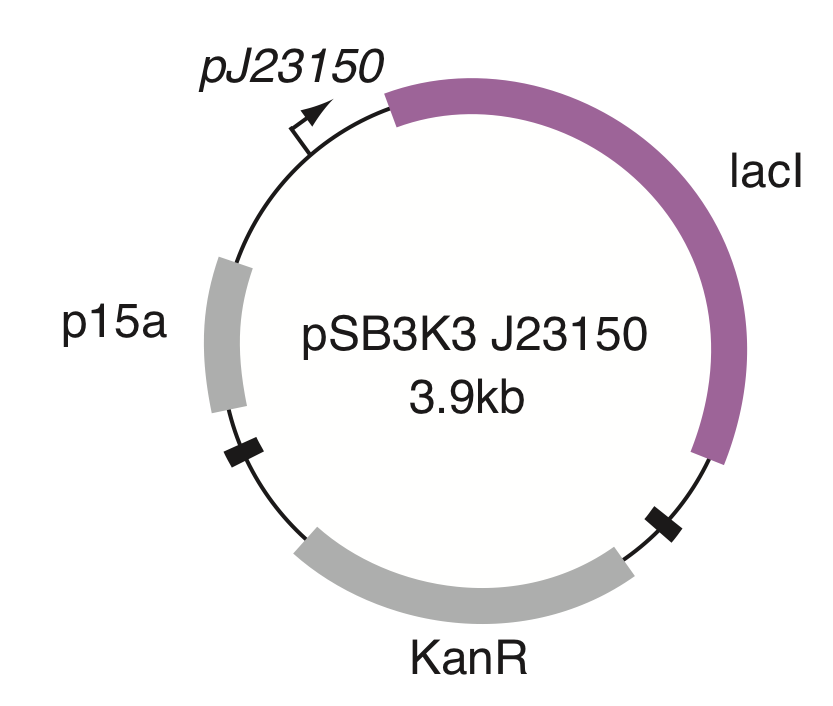

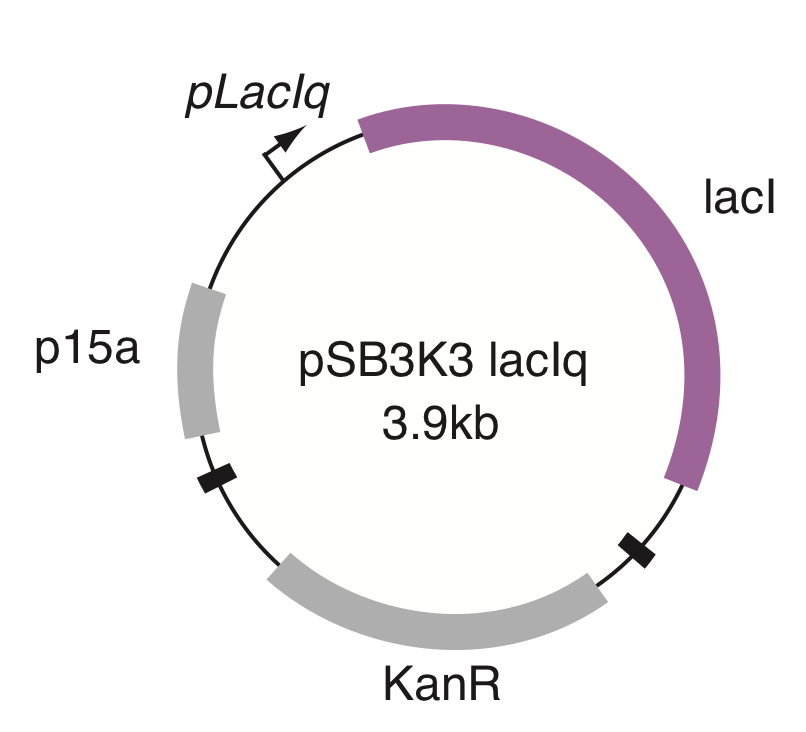


**Figure S4:** The plasmid maps for the lacI repressor expressing plasmids used in this study.

5. Quantitative Western Blots

All quantitative western blots used in this study are copied below. Images are the result of scans for the Cy-5 dye on a Typhoon variable mode imager. The photomultiplier tube sensitivity was adjusted to prevent detector oversaturation for each image.

**
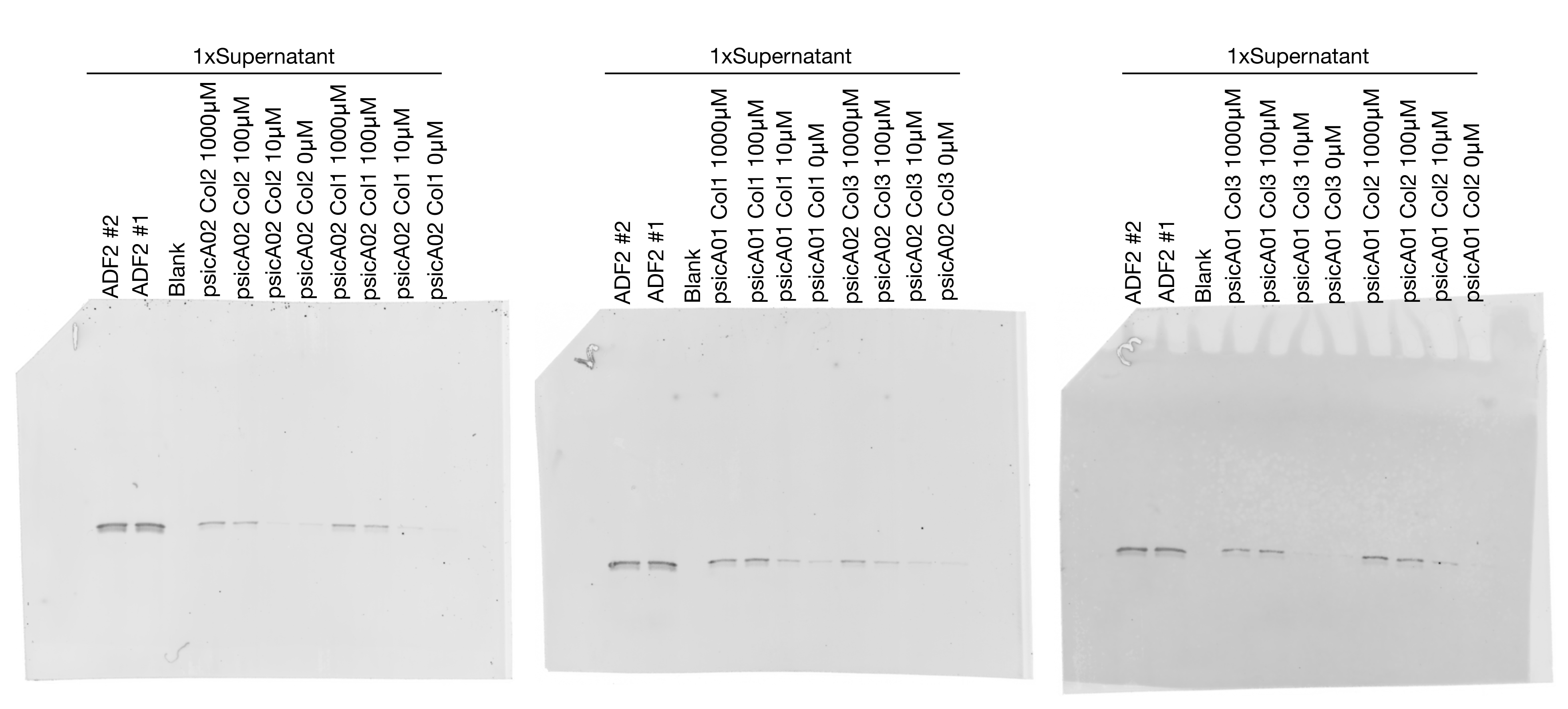
**

**
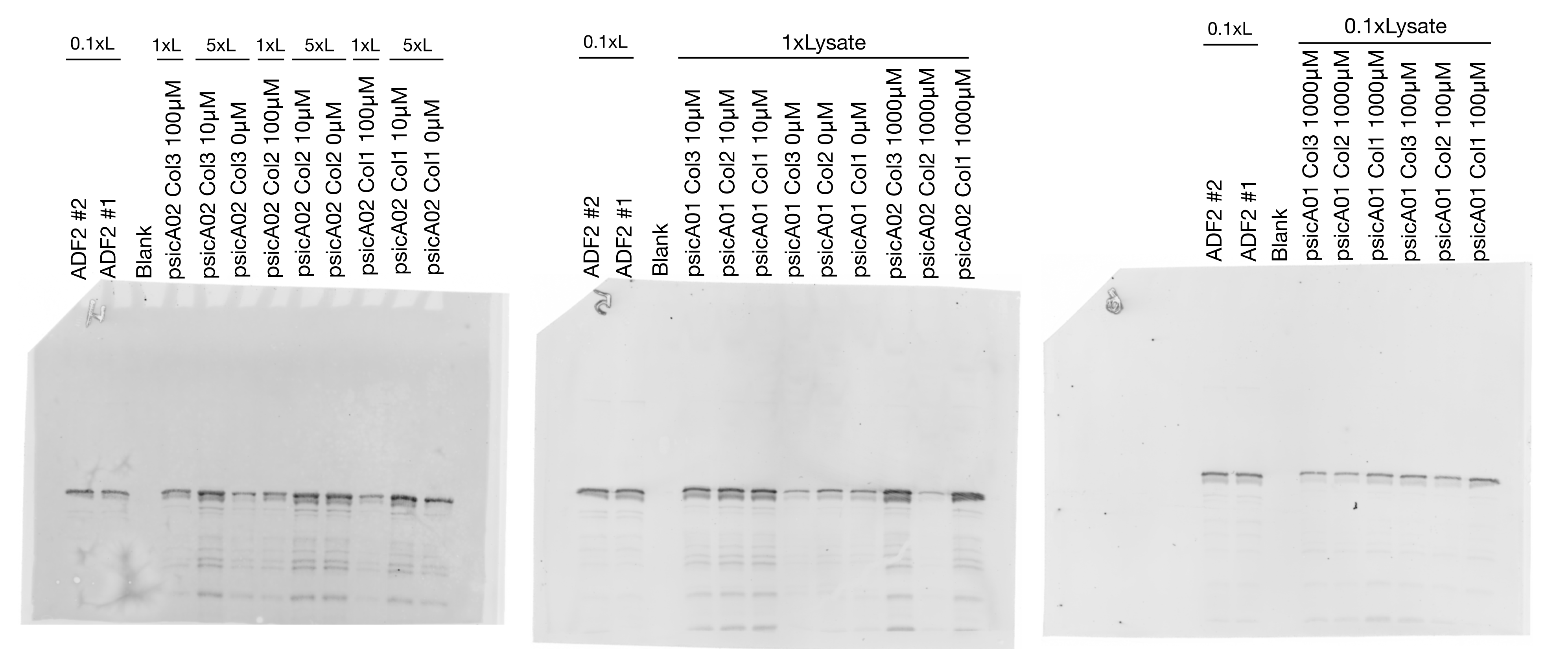
**

**Figure S5:** All gels used for quantitative western blotting analysis to determine the curves in Figure 4 and Figure S3. Each blot is labeled for the internal control bands (ADF2), the sample band with colony number and IPTG inducer concentration. The concentration of sample loaded is labeled above the lanes. Each gel has a 1x ADF2 supernatant or 0.1x ADF2 Lysate sample from to act as an internal standard [3]. Gels were processed according to materials and methods to determine signal intensity.

**
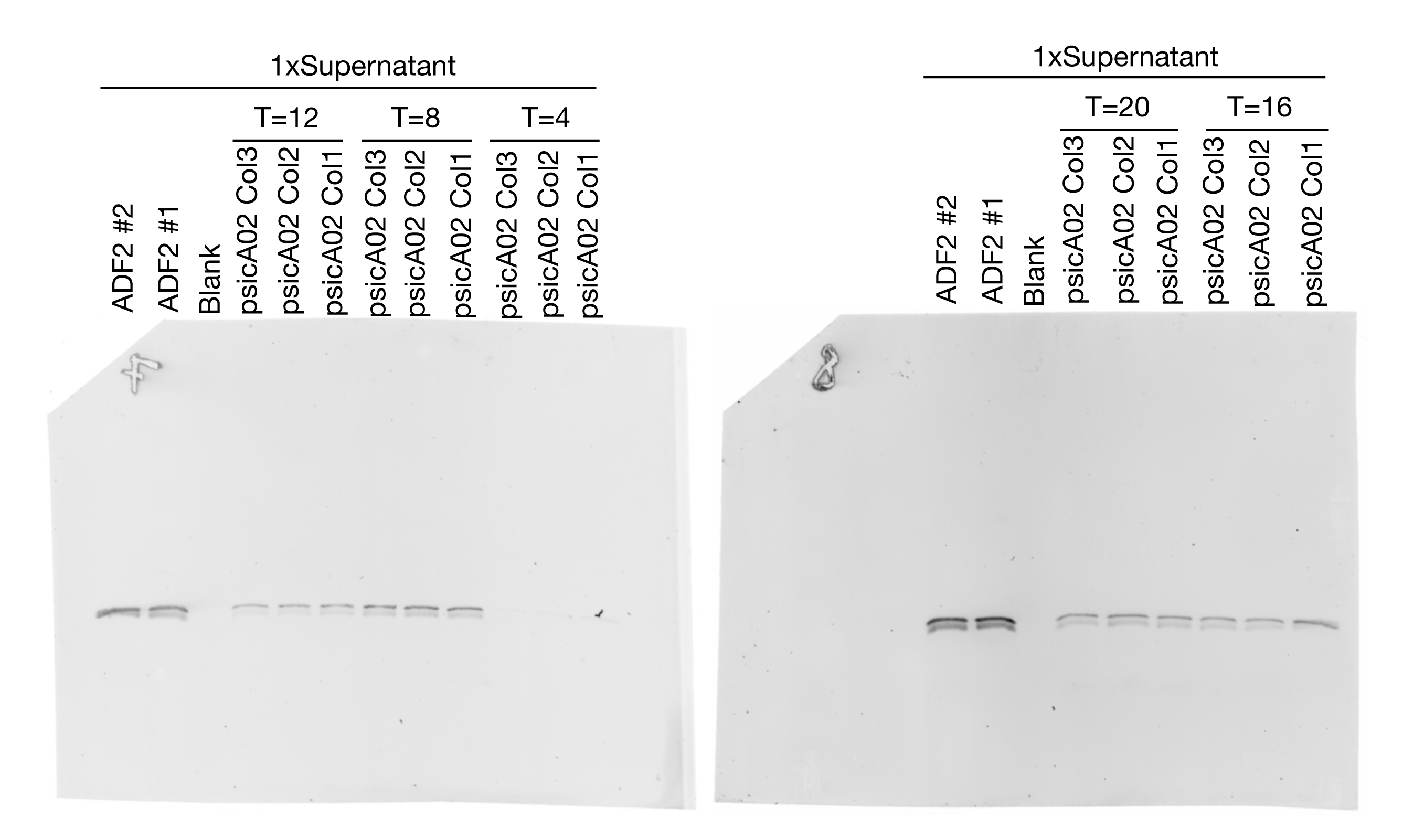
**

**
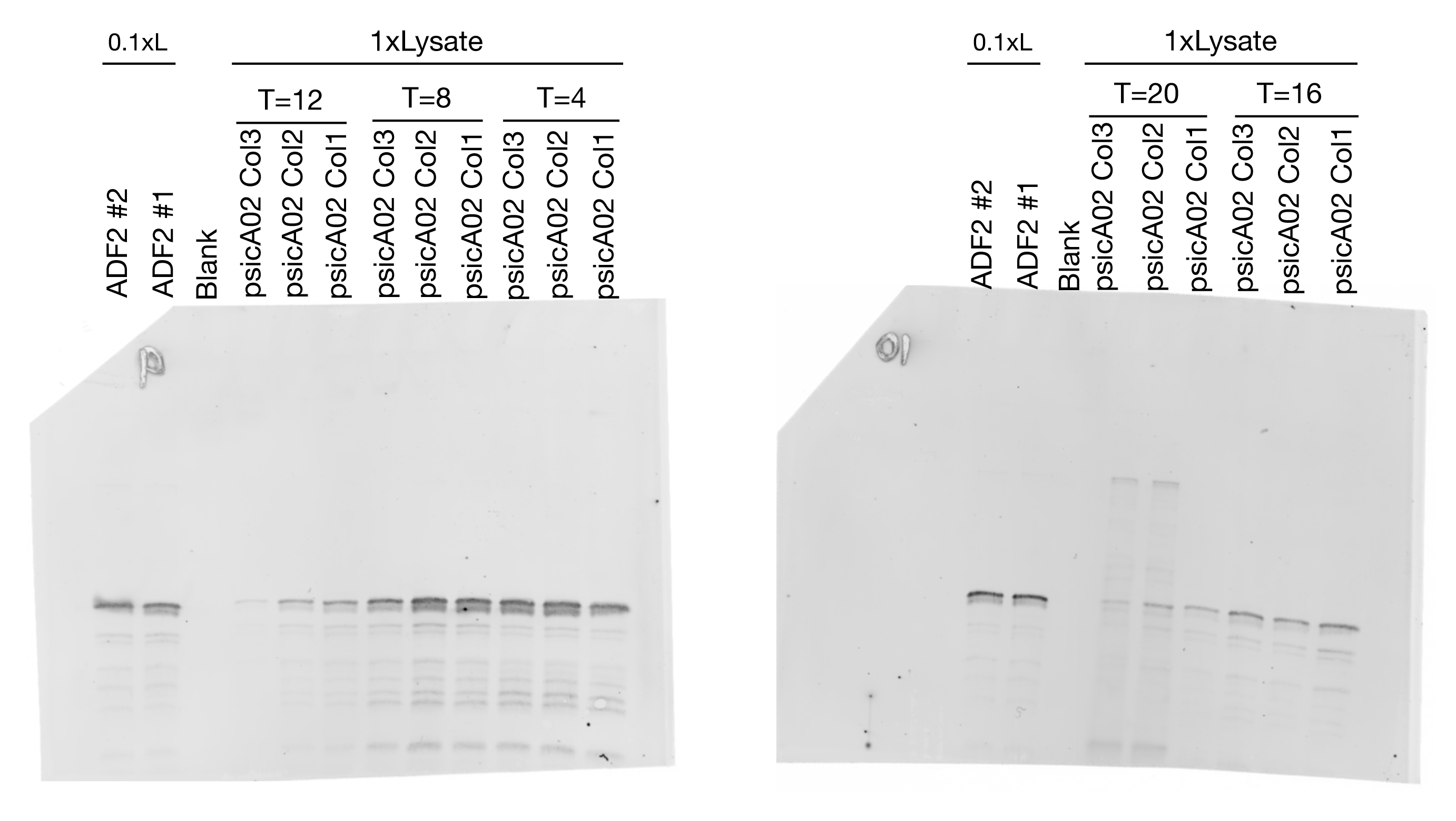
**

**Figure S6:** The blots to generate Figure 3 are shown. Each blot is labeled for the internal control bands (ADF2), the sample band with colony number and timepoint of harvest (in hours). The concentration of sample loaded is labeled above the lanes. Each gel has a 1x ADF2 supernatant or 0.1x ADF2 Lysate sample from to act as an internal standard [3]. Gels were processed according to materials and methods to determine signal intensity.

**
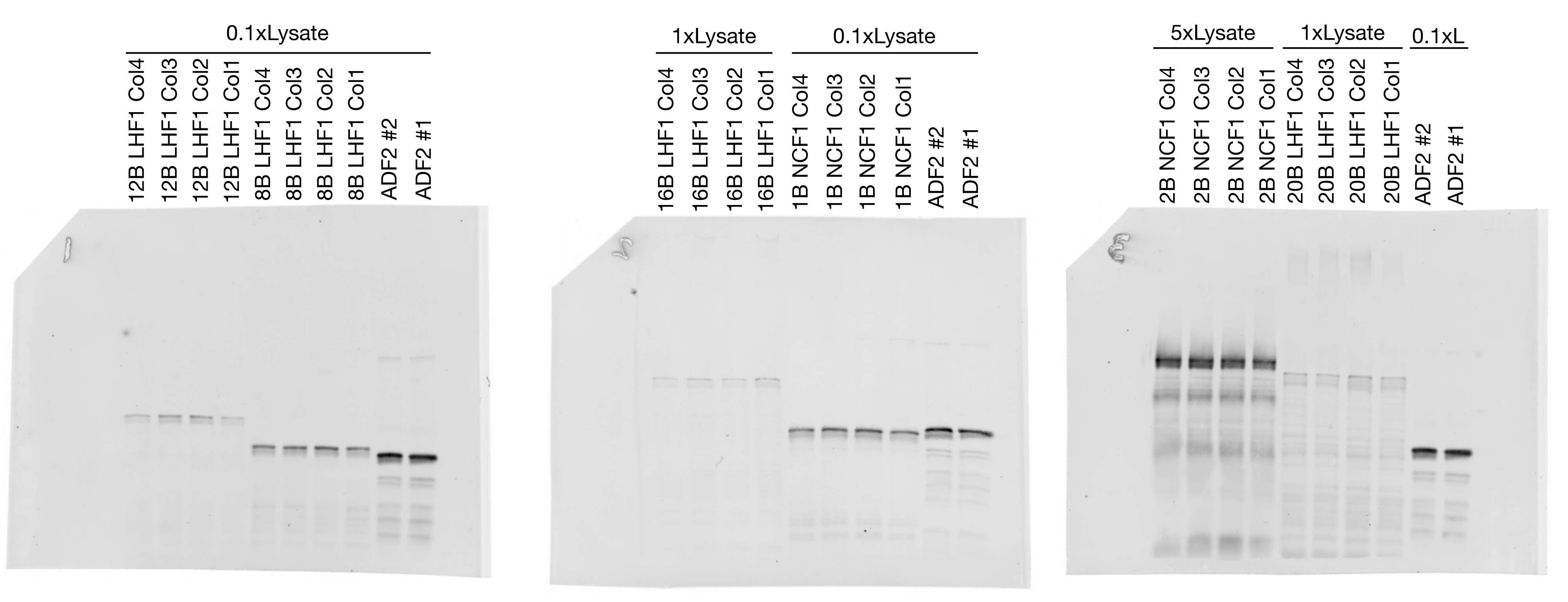
**

**
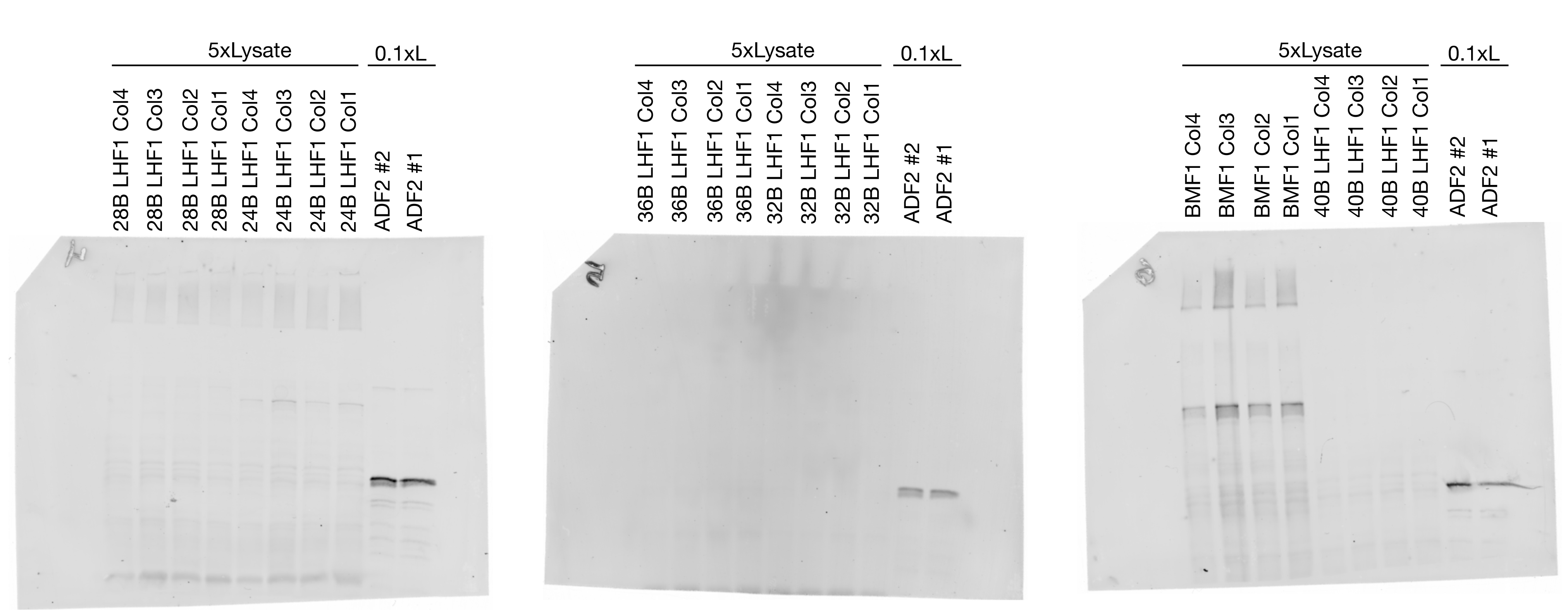
**

**
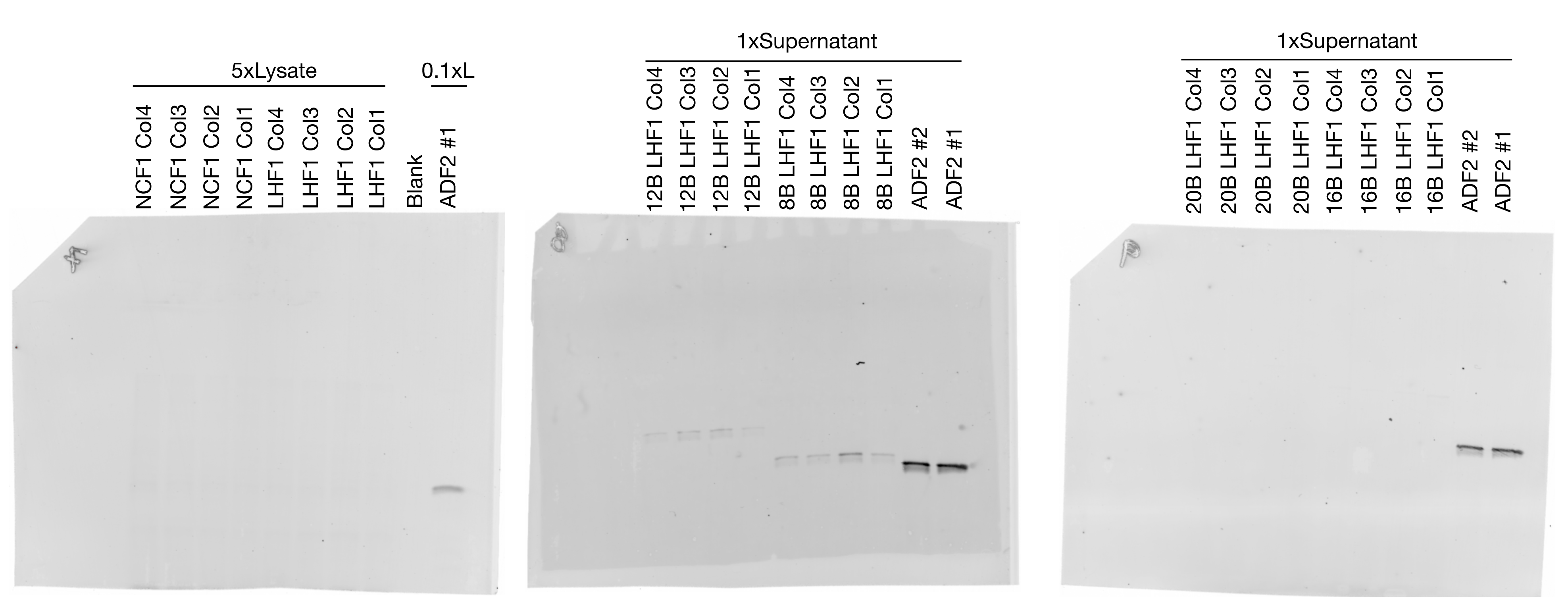
**

**
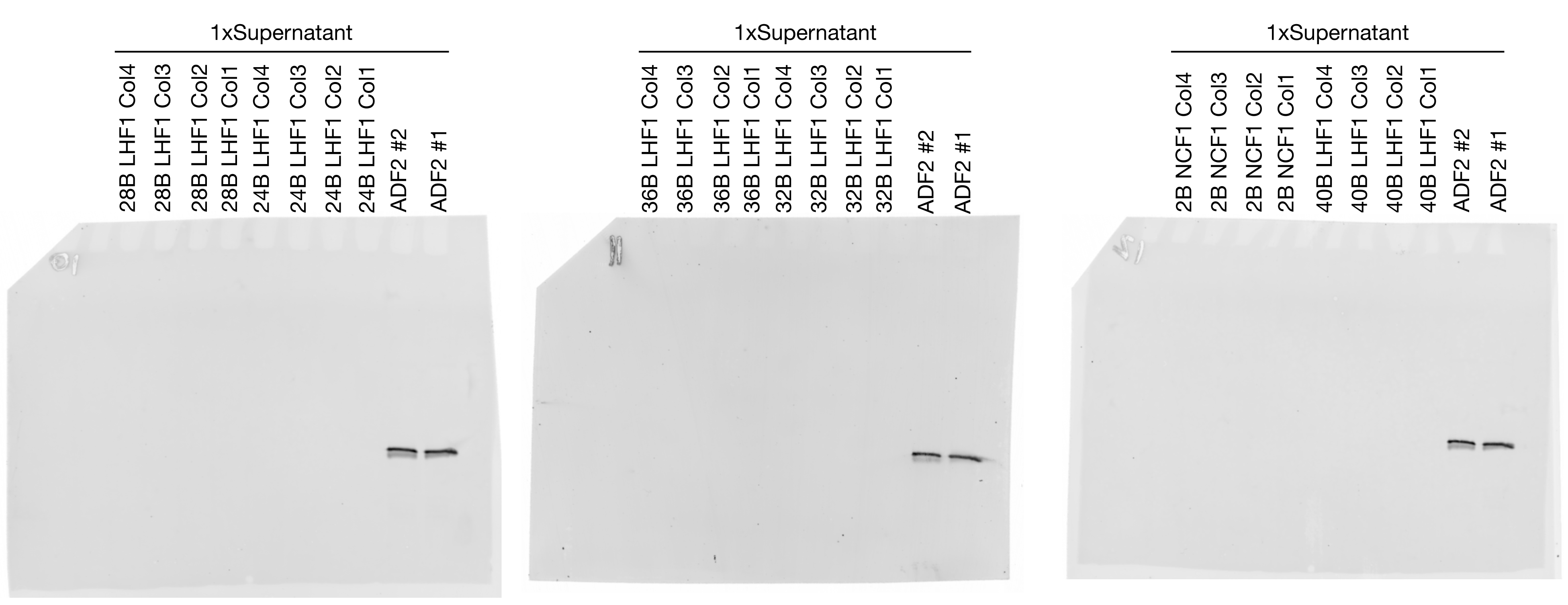
**

**
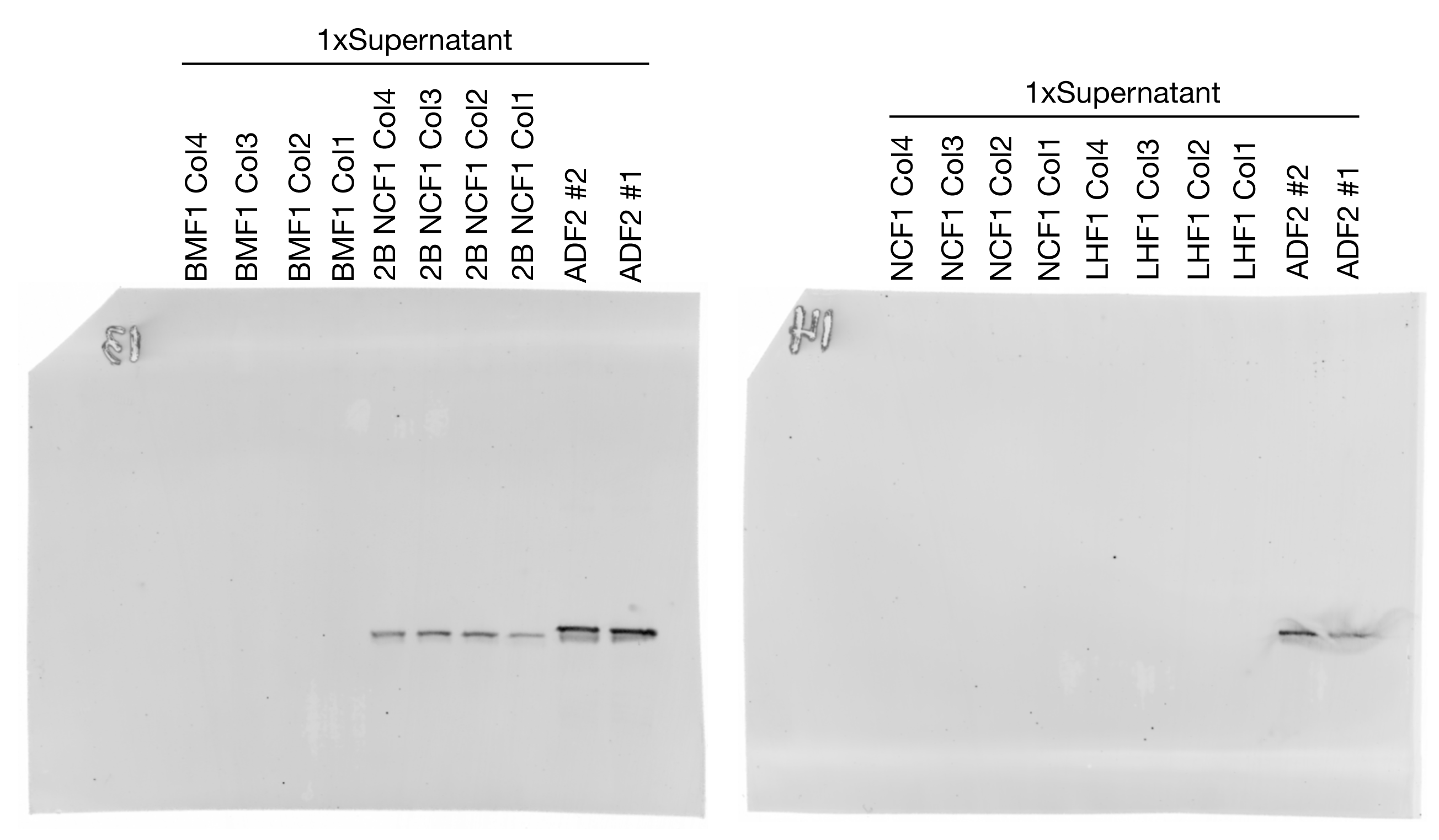
**

**
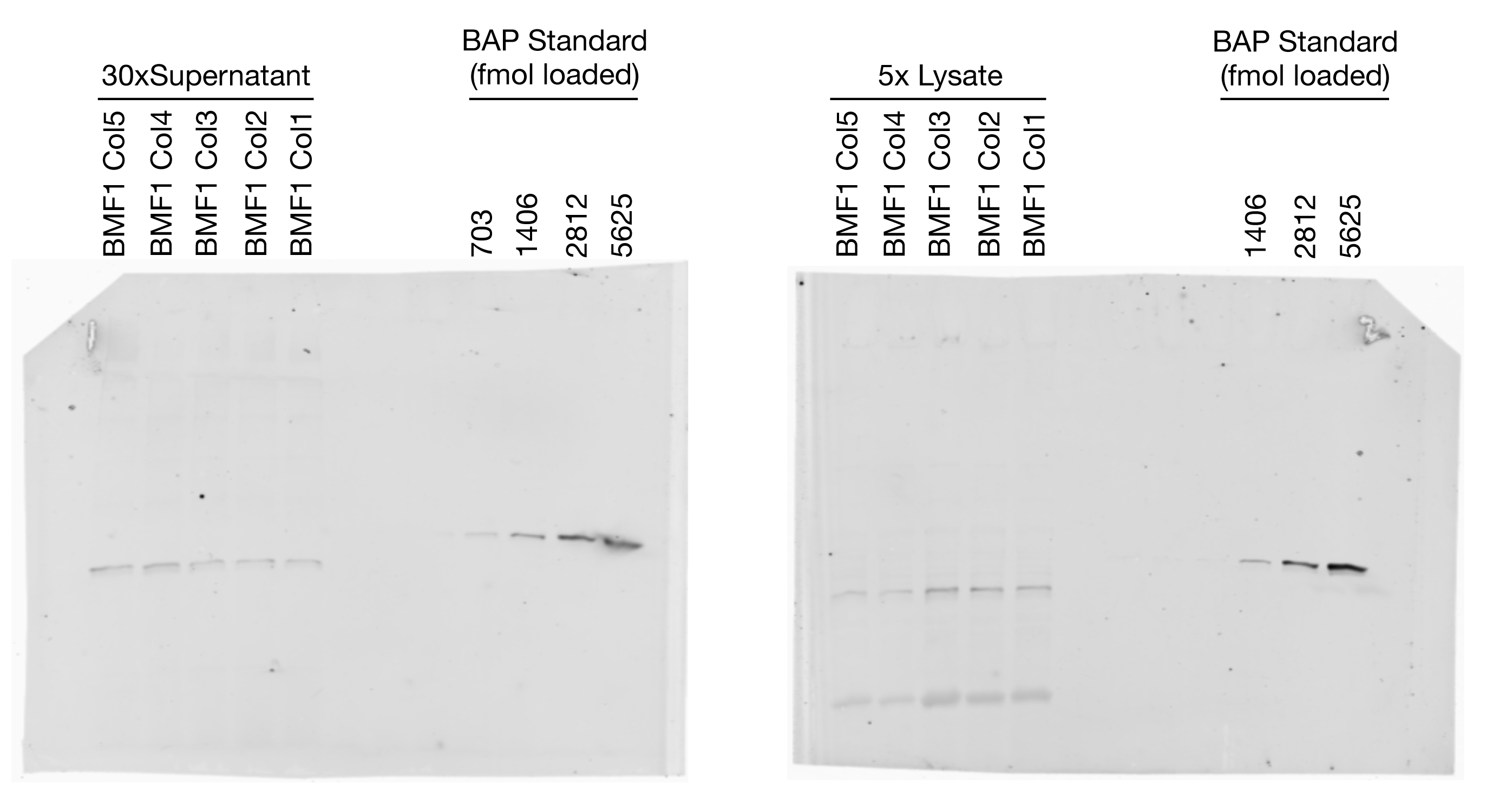
**

**Figure S7:** The blots used to generate Figure 5C and Figure 6 are shown. Each blot is labeled for the internal control bands (ADF2), the sample band with colony number and timepoint of harvest (in hours). The concentration of sample loaded is labeled above the lanes. Each gel has a 1x ADF2 supernatant or 0.1x ADF2 Lysate sample from to act as an internal standard [3]. Gels were processed according to materials and methods to determine signal intensity. The blots for ADF4 were run in the previously published method with an external bacterial alkaline phosphatase standard. Calculations were performed as published for the ADF4 bands [3].

6. Silk DNA and Amino Acid Sequences

All silk amino acid and synthetic DNA sequences used in this study. The originating organism, silk type, and ascension number (Genbank) are listed where appropriate.

***Latrodectus hesperus* Major Ampullate Spidroin 1 (LHF1), Genbank ascension EF595246** [4]

Synthetic DNA Sequence:

ATGACGTGGTCTACGCGCCTGGCACTGTCTTTCCTGTTTGTACTGTGCACTCAGTCCCTGTACGCACTCGCTCAGGCTAATACTCCGTGGTCTAGCAAGGCAAACGCAGATGCATTCATTAATTCCTTTATCTCCGCAGCCTCTAACACGGGCTCTTTTAGCCAAGACCAAATGGAGGACATGAGCCTGATCGGCAACACGCTGATGGCTGCCATGGATAACATGGGCGGTCGCATCACGCCGTCTAAGCTGCAAGCGCTGGACATGGCATTTGCATCCTCTGTGGCGGAGATCGCTGCCTCCGAGGGTGGTGATCTGGGTGTGACGACTAATGCAATCGCAGACGCTCTGACTTCCGCGTTCTACCAAACTACTGGTGTGGTGAACAGCCGTTTTATTTCCGAGATCCGCTCTCTGATCGGTATGTTCGCACAAGCCTCCGCCAATGATGTCTACGCTTCCGCAGGTTCTAGCGGTGGCGGCGGGTACGGGGCGTCTAGTGCTAGCGCAGCCTCAGCATCGGCCGCAGCCCCGTCGGGAGTTGCTTATCAAGCTCCGGCACAGGCTCAAATTAGCTTTACATTGCGTGGACAACAACCGGTATCTTACGGGCAAGGAGGCGCTGGGCCAGGCGGTGCCGGGGCAGCTGCAGCCGCAGCGGCTGCTGCGGGCGGGGCAGGACAGGGCGGGCAGGGAGGCTATGGGCAAGGCGGATACGGCCAGGGAGGTGCAGGCCAAGGAGGGTCAGGAGCTGCCGCCGCGGCCGCTGCTGCAGCAGGTGGAACGGGACAGGGTGGAGCAGGTCAGGGTGGCGCAGGTGCTGCCGCGGCTGCCGCTGCCGCAGCTGGAGGAGCCGGACAAGGAGGTCAGGGTGGTTACGGGCAGGGTGGTTATGGGCAGGGCGGAACAGGGCAGGGAGGTGCTGGCGCGGCCGCCGCAGCCGCAGCAGCAGGTGGAGCGGGTCAGGGAGGGCAAGGAGGCTACGGTCAGGGCGGTTATGGCCAGGGAGGGTATGGCCAGGGTGGATCCGGAGCTGCCGCAGCTGCAGCAGCGGCAGCCGGAGGCGCGGGCCAGGGCGGGCAGGGTGGGTATGGACAGGGAGGTTACGGCCAGGGAGGCGCCGGACAAGGCGGTGCAGGCGCTGCAGCCGCCGCTGCGGCCGCTGCAGGAGGAGCTGGACAAGGAGGCTACGGCAGGGGCGGCGCGGGACAGGGTGGCGCTGCCGCGGCGGCAGCAGCCGCTGCCGGTGCGGGCCAAGGCGGCTATGGCGGCCAGGGTGCAGGACAGGGTGGCAGTGGGGCAGCTGCGGCGGCCGCGGCAGCTGGTGGGGCAGGCCAAGGAGGGCAAGGCGGATACGGGCAAGGCGGGTATGGTCAAGGTGGCTCTGGTGCAGCGGCCGCGGCTGCTGCTGCAGGAGGGGCAGGCCAGGGAGGGCAGGGTGGGTATGGGCAGGGAGGATACGGCCAAGGAGGCGCGGGCCAGGGCGGAGCAGGGGCAGCTGCTGCGGCAGCGGCTGCAGGCGGCGCCGGCCAGGGAGGTCAGGGAGGATATGGACAGGGCGGGTACGGACAGGGTGGTGCGGGACAGGGCGGGGCGGGCGCGGCGGCAGCGGCGGCGGCTGCCGGAGGGGCTGGGCAGGGTGGACAAGGAGGTTATGGGCAGGGAGGCTATGGACAGGGAGGTGCTGGCCAGGGTGGCGCAGGCGCTGCTGCCGCTGCGGCCGCAGCAGGCGGAGCCGGACAGGGTGGTCAGGGTGGTTATGGACAAGGAGGCTACGGGCAGGGTGGAGCTGGCCAGGGCGGGGCGGCTGCTGCGGCCGCGGCAGCAGCAGGAGGAGCGGGCCAAGGAGGATACGGGAGGGGAGGGGCGGGACAGGGTGGTGCGGCGGCCGCTGCTGGTGCAGGACAAGGCGGGTACGGAGGCCAGGGTGCGGGTCAGGGCGGAGCAGGGGCTGCGGCTGCAGCAGCTGCGGCGGGAGGTGCAGGCCAAGGCGGACAAGGAGGGTATGGACGCGGAGGTTATGGGCAGGGCGGTGCTGGGCAAGGAGGGGCGGGCGCGGCTGCCGCCGCCGCTGCCGCAGGAGGCGCAGGGCAGGGTGGACAGGGCGGCTACGGTCAGGGCGGATATGGCCAAGGAGGGGCTGGACAAGGAGGAGCGGCAGCAGCCGCAGCAGCAGCGGGAGGTGCGGGGCAAGGAGGGTACGGCAGGGGCGGCGCAGGCCAGGGTGGAGCGGCGGCTGCCGCAGCCGCTGCGGCCGGAGCGGGTCAGGGTGGCTATGGAGGTCAGGGTGCCGGTCAGGGAGGGGCTGGTGCCGCGGCGGCAGCTGCAGCAGCTGGTGGCGCCGGGCAGGGCGGCCAAGGGGATTACGGTAGAGGCGGTTACGGGCAAGGCGGAGCCGGCCAAGGCGGTGCCGGAGCAGCGGCAGCTGCAGCCGCGGCCGGCGGAGCAGGCCAGGGCGGACAAGGCGGTTATGGCCAAGGAGGATACGGACAAGGAGGCGCAGGACAGGGTGGTGCGGCGGCGGCCGCAAGCGCAGCGGCAGCTGGCGGGGCCGGGCAGGGTGGGTATGGACGCGGAGGCGCGGGTCAGGGTGGCGCGGCGGCTGCGGCCGGTGCGGGGCAAGGAGGTTATGGAGGTCAGGGAGCGGGGCAAGGCGGAGCAGGAGCAGCAGCCGCAGCTGCTGCGGCGGGAGGAGCCGGCCAAGGAGGGCAGGGCGGATATGGTCGAGGAGGGTACGGTCAAGGAGGCGCAGGACAGGGAGGGGCGGGTGCTGCAGCCGCTGCAACCGCTGCGGGCGGGGCAGGCCAAGGCGGACAGGGAGGTTACGGACAGGGCGGGTATGGCCAGGGTGGTGCTGGGCAAGGCGGGGCGGCGGCAGCGGCCGCGGCTGCCGCAGGAGGTGCCGGCCAGGGCGGCTATGGACGGGGCGGTGCCGGACAGGGAGGAGCAGCGGCCGCGGCCGCTGCAGCAGCGGGTGCCGGCCAAGGAGGGTATGGCGGACAGGGAGCCGGTCAGGGTGGCGCAGGAGCCGCAGCCGCTGCCGCTGGTGGAGCTGGACAGGGTGGGCAGGGCGGATACGGCCGTGGCGGGTATGGTCAGGGAGGTGCTGGGCAAGGAGGAGCTGGAGCAGCCGCCGCCGCAGCAGCTGCAGGCGGCGCCGGCCAGGGTGGTCAAGGAGGTTACGGTCAAGGAGGATATGGCCAGGGTGGGGCAGGACAGGGCGGAGCTGCCGCGGCTGCCGCCGCAGCTGCCGGTGGTGCGGGTCAAGGAGGTTATGGACGAGGTGGCGCGGGGCAGGGAGGAGCAGCGGCGGCCGCAGGCGCGGGCCAGGGCGGGTACGGCGGGCAGGGCGCAGGCCAGGGCGGCGCGGGAGCTGCTGCTGCCGCTTCAAGAGGTGCAGGTCAAGGTGGTCAAGGAGGGTATGGCCGCGGAGGATATGGACAGGGTGGAGCTGGACAGGGAGGTGCGGGCGCGGCAGCCGCCGCGGCTGCGGCTGGTGGAGCTGGCCAAGGCGGTCAAGGCGGCTATGGTCAGGGCGGTTATGGGCAAGGAGGAGCCGGCCAGGGCGGGGCGGCGGCAGCGGCTGCTGCGGCGGGCGGCGCAGGCCAAGGAGGATACGGTCGTGGCGGTGCCGGCCAAGGAGGCGCAGCTGCCGCAGCGGGTGCGGGGCAGGGTGGCTACGGTGGGCAGGGTGCCGGTCAGGGTGGTGCCGGAGCGGCCGCCGCTGCTGCTGCAGCTGGTGGCGCGGGCCAGGGTGGACAGGGCGGATATGGCCGAGGAGGGTACGGACAGGGCGGCGCAGGGCAAGGCGGGGCAGGAGCCGCCGCCGCTGCTGCGGCGGCCGGAGGGGCTGGACAGGGTGGGCAGGGAGGCTATGGCCAGGGTGGTTATGGTCAAGGAGGAGCCGGACAGGGTGGTGCTGCCGCTGCCGCCGCGGCTGCTGCCGGTGGAGCCGGTCAGGGCGGCTACGGTCGAGGCGGTGCCGGCCAGGGCGGTGCGGCCGCTGCCGCAGCCGCTGCAGCAGGAAGCGGACAAGGTGGATATGGCGGTCAAGGCGCTGGGCAAGGAGGGGCCGGGGCGGCTGCAGCAGCAGCAGCTGCCGGAGGCGCGGGTCAAGGCGGCCAAGGCGGATACGGGCGAGGCGGATACGGTCAGGGAGGTGCGGGGCAAGGCGGAGCAGGCGCGGCAGCCGCTGCGGCCGCTGCGGGTGGGGCTGGTCAAGGAGGCCAGGGCGGATACGGCCAGGGTGGTTACGGACAGGGAGGAGCAGGGCAAGGCGGAGCTGCAGCCGCGGCAGCCGCCGCGGCCGCCGGAGGAGCGGGGCAGGGAGGGTACGGACGAGGAGGTGCTGGGCAGGGCGGCGCTGCCGCCGCCGCTGGCGCCGGCCAAGGAGGATACGGTGGTCAGGGTGCCGGACAGGGCGGAGCTGGAGCTGCCGCTGCAGCAGCCGCAGCAGGTGGGGCGGGACAGGGAGGTCAAGGCGGCTATGGACGGGGCGGATACGGGCAAGGAGGGGCTGGCCAGGGAGGGGCAGGTACTGCAGCTGCGGCAGCAGCGGCTGGCGGAGCAGGCCAAGGAGGACAGGGTGGGTACGGTCAAGGCGGGTATGGGCAGGGTGGGGCTGGACAGGGCGGTGCAGCCGCGGCAGCCGCTGCAGCCGCGGGCGGTGCGGGCCAAGGAGGTTACGGGCGAGGTGGTGCGGGTCAAGGCGGGGCCGCTGCCGCAGCTGCCGCAGCGGCAGGAGCTGGGCAGGGAGGATACGGAGGGCAAGGGGCGGGTCAAGGCGGGGCTGGCGCGGCAGCCGCTGCTGCAGCTGCTGGAGGAGCGGGGCAGGGTGGCCAAGGTGGTTATGGTCGCGGAGGTTACGGACAAGGAGGGGCCGGCCAGGGCGGCGCAGGAGCGGCGGCGGCTGCGGCTGCAGCGGGTGGCGCGTCCCAGGGTGGACAAGGTGGGTACGGTCAAGGGGATTATGGGCAAGGAGGAGCAGGTCAGGGTGGGGCTGCTGCTGCGGCCGCGGCGGCCGGTGGAGCAGGACAGGGAGGGTATGGACGTGGTGGCGCAGGCCAAGGCGGAGCTGCTGCCGCTGCCGGAGCAGGACAAGGAGGTTACGGAGGGCAGGGTGCTGGGCAGGGAGGAGCGGGTGCAGCGGCAGCTGCGGCGGCGGCTGGTGGTGCCGGAAGGGGTGGGCAAGGCGGGTATGGACGTGGCGGATACGGGCAGGGAGGGGCAGGCCAAGGTGGTGCGGGTGCTGCGGCCGCGGCGGCAGCGGCCGGTGGTGCTGGCCAAGGTGGCCAGGGTGGCTACGGACAAGGTGGGTATGGTCAAGGTGGAACTGGCCAAGGCGGTGCAGCTGCTGCTGCCGCGGCTGCTGCGGGCGGAGCTGGACAAGGAGGCTATGGTCGGGGTGGAGCAGGGCAAGGAGGGGCTGCAGCCGCTGCGGCTGCGGCAGCCGGGGCCGGTCAGGGTGGTTATGGAGGGCAGGGCGCGGGTCAAGGCGGGGCTGGGGCGGCTGCTGCTGCAGCAGCGGCCGGCGGCGCCGGGCAGGGTGGACAGGGTGGCTACGGAAGAGGAGGCTACGGTCAAGGAGGCGCAGGCCAAGGCGGGGCAGGAGCTGCTGCTGCCGCGGCGGCCGCGGGTGGTGCAGGGCAAGGAGGACAGGGAGGGTACGGCCAAGGAGGATATGGGCAAGGCGGTTACGGTCAGGGTGGAGCTGGACAAGGCGGAGCGGCTGCCGCTGCTGCAGCTGCTGGCGGCGCGGGACAAGGTGGTTACGGGAGGGGAGGTGCCGGACAGGGTGGCGCTGCAGCCGCCGCTGGTGCTGGCCAAGGAGGTTACGGTGGCCAAGGTGCTGGGCAGGGTGGGGCTGGTGCGGCTGCTGCGGCGGCAGCTGCGGGAGGAGCAGGGCAGGGCGGACAAGGTGGCTATGGCAGGGGCGGTTATGGACAGGGCGGCGCAGGACAAGGAGGAGCGGGCGCGGCGGCTGCAGCAGCCGCTGCCGGAGGCGCCGGTCAGGGTGGCCAGGGCGGGTACGGCCAAGGCGGATACGGTCAAGGCGGGGCAGGACAGGGTGGCGCTGCGGCTGCGGCAGCAGCAGCGGCAGGAGGGGCGGGCCAAGGCGGATATGGGCGGGGCGGAGCGGGACAAGGCGGAGCGGCAGCTGCAGCGGCCGCAGCGGCGGGTTCAGGCCAGGGAGGTTACGGCGGCCAAGGTGCCGGACAGGGAGGAGCAGGCGCCGCGGCCGCCGCAGCAGCTGCGGGAGGCGCCGGGCAGGGAGGGCAAGGAGGTTACGGGCGTGGCGGCTACGGACAAGGTGGCGCGGGCCAGGGTGGGGCTGGCGCAGCGGCGGCGGCTGCCGCCGCAGGCGGCGCTGGGCAAGGCGGGCAGGGTGGATACGGGCAGGGCGGATATGGCCAGGGTGGGTACGGTCAAGGAGGTGCCGGACAGGGAGGAGCAGCGGCAGCAGCTGCGGCGGCGGCAGCTGGAGGGGCAGGGCAGGGCGGTTATGGTCGTGGAGGGGCCGGACAAGGAGGTGCAGCTGCAGCAGCGGGAGCTGGGCAGGGTGGATACGGAGGTCAGGGCGCAGGACAGGGTGGTGCTGGCGCTGCGGCTGCGGCAGCCGCGGCGGGAGGCGCGGGGCAGGGAGGCCAAGGCGGGTATGGAAGAGGAGGCTATGGGCAAGGCGGTGCGGGACAAGGTGGGGCCGGTGCTGCGGCGGCTGCAGCGGCTGCTGGAGGTGCTGGCCAAGGAGGGCAAGGCGGATACGGTCAGGGTGGAAATGGGCAAGGCGGAGCGGGCCAGGGCGGCGCGGCCGCTGCCGCGGCCGCAGCAGGCGGCGCGGGCCAAGGCGGGTACGGTCGTGGCGGGGCCGGACAAGGCGGAGCTGCCGCCGCCGCTGCGGCCGCGGCTGGGGCTGGGCAGGGTGGATATGGAGGGCAAGGCGCGGGACAGGGTGGTGCAGGCGCGGCTGCCGCTGCTGCTGCGGCGGGCGGAGCAGGGCAGGGCGGCCAGGGTGGATACGGGAGAGGTGGCTACGGTCAAGGAGGGGCCGGCCAAGGTGGTGCGGGAGCAGCCGCTGCAGCTGCGGCCGCTGGTGGGGCTTCGCAGGGTGGCCAGGGTGGTTACGGGCAAGGTGATTACGGGCAGGGAGGAGCGGGACAAGGTGGGGCCGCCGCTGCGGCTGCGGCAGCAGGTGGCGCTGGACAAGGAGGGTATGGACGAGGAGGGGCAGGCCAAGGCGGGGCTGCCGCTGCTGCAGGGGCAGGTCAAGGCGGGTACGGCGGACAAGGTGCAGGGCAAGGAGGTGCTGGAGCTGCGGCTGCAGCAGCAGCTGCGGGTGGGGCAGGTAGAGGAGGCCAAGGAGGCTACGGAAGAGGCGGATACGGTCAGGGCGGTGCGGGCCAGGGTGGTGCTGGTGCTGCCGCAGCCGCCGCTGCTGCCGGTGGGGCTGGACAGGGTGGACAAGGTGGGTACGGACAAGGTGGCTACGGGCAGGGAGGCGCGGGTCAAGGAGGGGCGGCGGCAGCCGCTGCAGCTGCCGCTGGTGGCGCCGGACAAGGAGGCTATGGCCGTGGTGGGGCGGGGCAAGGCGGCGCGGCGGCCGCGGCGGGTGCGGGGCAGGGCGGCTATGGAGGTCAAGGAGCCGGCCAAGGTGGGGCAGGCGCAGCAGCCGCTGCGGCCGCTGCCGGAGGTGCTGGGCGAGGAGGGCAGGGTGGGTATGGTAGAGGCGGGTACGGGCAGGGAGGCGCCGGACAGGGAGGGGCTGGTGCCGCTGCGGCTGCGGCAGCCGCCGGAGGAGCGGGTCAGGGTGGCCAGGGCGGCTACGGTCAGGGCGGGTATGGGCAGGGCGGGGCTGGACAAGGTGGCGCCGCGGCAGCTGCAGCAGCTGCGGTGGGAGGGGCGGGTCAGGGTGGATACGGCCGAGGTGGGGCAGGTCAAGGCGGTGCTGCGGCCGCGGCGGCCGCTGCAGCCGCTGGTTCTGGTCAGGGAGGTTATGGAGGCCAAGGAGCAGGCCAAGGCGGCGCCGGGGCCGCTGCCGCCGCCGCTGCTGCTGGCGGAGCAGGGCAGGGTGGACAGGGCGGTTACGGTGGTGGAGGATACGGACAAGGAGGTGCCGGCCAAGGCGGAGCTGGAGCTGCTGCGGCAGCAGCAGCCGCGGGAGGCGCTGGACAGGGTGGGCAGGGCGGCTACGGTCAGGGTGGATACGGTCAGGGCGGGGCGGGGCAGGGTGGAGCCGCAGCGGCTGCAGCTGCGGCCGCGGGCGGAGCAGGGCAGGGAGGCTATGGTCGGGGTGGAGCCGGACAAGGAGGCGCGGCGGCCGCTACCGGTGCTGGACAAGGTGGCTATGGCGGTCAAGGAGCAGGACAAGGCGGAGCGGGCGCCGCTGCTGCAGCGGCAGCTGCTGGTGGGGCCGGTCAGGGTGGCCAAGGAGGATACGGCAGGGGTGGCTACGGCCAAGGCGGTGCAGGTCAAGGAGGTGCTGGGGCCGCTGCCGCCGCCGCAGCCGCCGGAGGAGCTGGGCAAGGCGGACAAGGAGGGTATGGCCAAGGTGGGTACGGGCAGGGGGGTGCCGGACAAGGCGGAGCGGCCGCAGCGGCTGCAGCCGCCGGTGGTGCTGGCCAGGGTGGCTATGGGAGAGGCGGGGCCGGTCAAGGAGGCGCCGCGGCAGCTGCGGCGGCCGCGGCAGGGGCGGGGCAGGGCGGTTATGGAGGACAAGGCGCGGGTCAGGGTGGAGCGGGTGCGGCGGCGGCAGCGGCAGCTGCGGGCGGCGCTGGGCAAGGAGGTCAAGGCGGGTACGGTCGAGGTGGCTACGGACAGGGAGGGGCTGGACAAGGTGGGGCGGGTGCCGCAGCGGCGGGAGGAGCCGGTCAGGGTGGGCAAGGCGGCTACGGTCAGGGTGGATATGGCCAAGGTGGTGCAGGGCAGGGTGGGGCTGCAGCTGCTGCCGCAGCCGCAGCCGCAGGCGGCGCGGGCCAAGGAGGATACGGCGGATATGGTCAGCAAGGCGGAGCTGGAGCGGCTGCTGCAGCGGCTTCAGGACCGGGCCAGATCTACTACGGTCCGCAGTCCGTTGCCGCCCCAGCTGCTGCTGCGGCGTCTGCGTTGGCAGCACCGGCCACTTCTGCACGCATTTCGTCGCACGCGTCCGCATTGCTTAGTAATGGGCCGACAAATCCTGCGTCCATCTCCAACGTAATCAGTAACGCAGTAAGCCAAATTTCTAGCTCTAACCCAGGCGCTTCTGCCTGTGATGTGCTAGTTCAGGCCCTACTTGAGCTCGTAACGGCGCTATTGACTATTATCGGCTCATCAAATATTGGTAGTGTGAACTATGACAGTTCGGGCCAGTACGCACAAGTCGTTACTCAGTCAGTTCAAAACGCCTTCGCC

Amino Acid Sequence:

MTWSTRLALSFLFVLCTQSLYALAQANTPWSSKANADAFINSFISAASNTGSFSQDQMEDMSLIGNTLMAAMDNMGGRITPSKLQALDMAFASSVAEIAASEGGDLGVTTNAIADALTSAFYQTTGVVNSRFISEIRSLIGMFAQASANDVYASAGSSGGGGYGASSASAASASAAAPSGVAYQAPAQAQISFTLRGQQPVSYGQGGAGPGGAGAAAAAAAAAGGAGQGGQGGYGQGGYGQGGAGQGGSGAAAAAAAAAGGTGQGGAGQGGAGAAAAAAAAAGGAGQGGQGGYGQGGYGQGGTGQGGAGAAAAAAAAGGAGQGGQGGYGQGGYGQGGYGQGGSGAAAAAAAAAGGAGQGGQGGYGQGGYGQGGAGQGGAGAAAAAAAAAGGAGQGGYGRGGAGQGGAAAAAAAAAGAGQGGYGGQGAGQGGSGAAAAAAAAGGAGQGGQGGYGQGGYGQGGSGAAAAAAAAGGAGQGGQGGYGQGGYGQGGAGQGGAGAAAAAAAAGGAGQGGQGGYGQGGYGQGGAGQGGAGAAAAAAAAGGAGQGGQGGYGQGGYGQGGAGQGGAGAAAAAAAAGGAGQGGQGGYGQGGYGQGGAGQGGAAAAAAAAAGGAGQGGYGRGGAGQGGAAAAAGAGQGGYGGQGAGQGGAGAAAAAAAAGGAGQGGQGGYGRGGYGQGGAGQGGAGAAAAAAAAGGAGQGGQGGYGQGGYGQGGAGQGGAAAAAAAAGGAGQGGYGRGGAGQGGAAAAAAAAAGAGQGGYGGQGAGQGGAGAAAAAAAAGGAGQGGQGDYGRGGYGQGGAGQGGAGAAAAAAAAGGAGQGGQGGYGQGGYGQGGAGQGGAAAAASAAAAGGAGQGGYGRGGAGQGGAAAAAGAGQGGYGGQGAGQGGAGAAAAAAAAGGAGQGGQGGYGRGGYGQGGAGQGGAGAAAAATAAGGAGQGGQGGYGQGGYGQGGAGQGGAAAAAAAAAGGAGQGGYGRGGAGQGGAAAAAAAAAGAGQGGYGGQGAGQGGAGAAAAAAGGAGQGGQGGYGRGGYGQGGAGQGGAGAAAAAAAAGGAGQGGQGGYGQGGYGQGGAGQGGAAAAAAAAAGGAGQGGYGRGGAGQGGAAAAAGAGQGGYGGQGAGQGGAGAAAAASRGAGQGGQGGYGRGGYGQGGAGQGGAGAAAAAAAAGGAGQGGQGGYGQGGYGQGGAGQGGAAAAAAAAGGAGQGGYGRGGAGQGGAAAAAGAGQGGYGGQGAGQGGAGAAAAAAAAGGAGQGGQGGYGRGGYGQGGAGQGGAGAAAAAAAAGGAGQGGQGGYGQGGYGQGGAGQGGAAAAAAAAAGGAGQGGYGRGGAGQGGAAAAAAAAAGSGQGGYGGQGAGQGGAGAAAAAAAAGGAGQGGQGGYGRGGYGQGGAGQGGAGAAAAAAAAGGAGQGGQGGYGQGGYGQGGAGQGGAAAAAAAAAAGGAGQGGYGRGGAGQGGAAAAAGAGQGGYGGQGAGQGGAGAAAAAAAAGGAGQGGQGGYGRGGYGQGGAGQGGAGTAAAAAAAGGAGQGGQGGYGQGGYGQGGAGQGGAAAAAAAAAGGAGQGGYGRGGAGQGGAAAAAAAAAGAGQGGYGGQGAGQGGAGAAAAAAAAGGAGQGGQGGYGRGGYGQGGAGQGGAGAAAAAAAAGGASQGGQGGYGQGDYGQGGAGQGGAAAAAAAAGGAGQGGYGRGGAGQGGAAAAAGAGQGGYGGQGAGQGGAGAAAAAAAAGGAGRGGQGGYGRGGYGQGGAGQGGAGAAAAAAAAGGAGQGGQGGYGQGGYGQGGTGQGGAAAAAAAAAGGAGQGGYGRGGAGQGGAAAAAAAAAGAGQGGYGGQGAGQGGAGAAAAAAAAGGAGQGGQGGYGRGGYGQGGAGQGGAGAAAAAAAAGGAGQGGQGGYGQGGYGQGGYGQGGAGQGGAAAAAAAAGGAGQGGYGRGGAGQGGAAAAAGAGQGGYGGQGAGQGGAGAAAAAAAAGGAGQGGQGGYGRGGYGQGGAGQGGAGAAAAAAAAGGAGQGGQGGYGQGGYGQGGAGQGGAAAAAAAAAGGAGQGGYGRGGAGQGGAAAAAAAAAGSGQGGYGGQGAGQGGAGAAAAAAAAGGAGQGGQGGYGRGGYGQGGAGQGGAGAAAAAAAAGGAGQGGQGGYGQGGYGQGGYGQGGAGQGGAAAAAAAAAAGGAGQGGYGRGGAGQGGAAAAAGAGQGGYGGQGAGQGGAGAAAAAAAAGGAGQGGQGGYGRGGYGQGGAGQGGAGAAAAAAAAGGAGQGGQGGYGQGGNGQGGAGQGGAAAAAAAAGGAGQGGYGRGGAGQGGAAAAAAAAAGAGQGGYGGQGAGQGGAGAAAAAAAAGGAGQGGQGGYGRGGYGQGGAGQGGAGAAAAAAAAGGASQGGQGGYGQGDYGQGGAGQGGAAAAAAAAGGAGQGGYGRGGAGQGGAAAAAGAGQGGYGGQGAGQGGAGAAAAAAAAGGAGRGGQGGYGRGGYGQGGAGQGGAGAAAAAAAAGGAGQGGQGGYGQGGYGQGGAGQGGAAAAAAAAAGGAGQGGYGRGGAGQGGAAAAAGAGQGGYGGQGAGQGGAGAAAAAAAAGGAGRGGQGGYGRGGYGQGGAGQGGAGAAAAAAAAGGAGQGGQGGYGQGGYGQGGAGQGGAAAAAAAAVGGAGQGGYGRGGAGQGGAAAAAAAAAAGSGQGGYGGQGAGQGGAGAAAAAAAAGGAGQGGQGGYGGGGYGQGGAGQGGAGAAAAAAAAGGAGQGGQGGYGQGGYGQGGAGQGGAAAAAAAAAGGAGQGGYGRGGAGQGGAAAATGAGQGGYGGQGAGQGGAGAAAAAAAAGGAGQGGQGGYGRGGYGQGGAGQGGAGAAAAAAAAGGAGQGGQGGYGQGGYGQGGAGQGGAAAAAAAAGGAGQGGYGRGGAGQGGAAAAAAAAAGAGQGGYGGQGAGQGGAGAAAAAAAAGGAGQGGQGGYGRGGYGQGGAGQGGAGAAAAGGAGQGGQGGYGQGGYGQGGAGQGGAAAAAAAAAAGGAGQGGYGGYGQQGGAGAAAAAASGPGQIYYGPQSVAAPAAAAASALAAPATSARISSHASALLSNGPTNPASISNVISNAVSQISSSNPGASACDVLVQALLELVTALLTIIGSSNIGSVNYDSSGQYAQVVTQSVQNAFA

**8B LHF1:**

Synthetic DNA Sequence:

GGTGGGGCCGGTCAGGGTGGCCAAGGAGGATACGGCAGGGGTGGCTACGGCCAAGGCGGTGCAGGTCAAGGAGGTGCTGGGGCCGCTGCCGCCGCCGCAGCCGCCGGAGGAGCTGGGCAAGGCGGACAAGGAGGGTATGGCCAAGGTGGGTACGGGCAGGGGGGTGCCGGACAAGGCGGAGCGGCCGCAGCGGCTGCAGCCGCCGGTGGTGCTGGCCAGGGTGGCTATGGGAGAGGCGGGGCCGGTCAAGGAGGCGCCGCGGCAGCTGCGGCGGCCGCGGCAGGGGCGGGGCAGGGCGGTTATGGAGGACAAGGCGCGGGTCAGGGTGGAGCGGGTGCGGCGGCGGCAGCGGCAGCTGCGGGCGGCGCTGGGCAAGGAGGTCAAGGCGGGTACGGTCGAGGTGGCTACGGACAGGGAGGGGCTGGACAAGGTGGGGCGGGTGCCGCAGCGGCGGGAGGAGCCGGTCAGGGTGGGCAAGGCGGCTACGGTCAGGGTGGATATGGCCAAGGTGGTGCAGGGCAGGGTGGGGCTGCAGCTGCTGCCGCAGCCGCAGCCGCAGGCGGCGCGGGCCAAGGAGGATACGGCGGATATGGTCAGCAAGGCGGAGCTGGAGCGGCTGCTGCAGCGGCTTCAGGACCGGGCCAGATCTACTACGGTCCGCAGTCCGTTGCCGCCCCAGCTGCTGCTGCGGCGTCTGCGTTGGCAGCACCGGCCACTTCTGCACGCATTTCGTCGCACGCGTCCGCATTGCTTAGTAATGGGCCGACAAATCCTGCGTCCATCTCCAACGTAATCAGTAACGCAGTAAGCCAAATTTCTAGCTCTAACCCAGGCGCTTCTGCCTGTGATGTGCTAGTTCAGGCCCTACTTGAGCTCGTAACGGCGCTATTGACTATTATCGGCTCATCAAATATTGGTAGTGTGAACTATGACAGTTCGGGCCAGTACGCACAAGTCGTTACTCAGTCAGTTCAAAACGCCTTCGCC

Amino Acid Sequence:

GGAGQGGQGGYGRGGYGQGGAGQGGAGAAAAAAAAGGAGQGGQGGYGQGGYGQGGAGQGGAAAAAAAAGGAGQGGYGRGGAGQGGAAAAAAAAAGAGQGGYGGQGAGQGGAGAAAAAAAAGGAGQGGQGGYGRGGYGQGGAGQGGAGAAAAGGAGQGGQGGYGQGGYGQGGAGQGGAAAAAAAAAAGGAGQGGYGGYGQQGGAGAAAAAASGPGQIYYGPQSVAAPAAAAASALAAPATSARISSHASALLSNGPTNPASISNVISNAVSQISSSNPGASACDVLVQALLELVTALLTIIGSSNIGSVNYDSSGQYAQVVTQSVQNAFA

**12B LHF1:**

Synthetic DNA Sequence:

GGCGGAGCAGGGCAGGGTGGACAGGGCGGTTACGGTGGTGGAGGATACGGACAAGGAGGTGCCGGCCAAGGCGGAGCTGGAGCTGCTGCGGCAGCAGCAGCCGCGGGAGGCGCTGGACAGGGTGGGCAGGGCGGCTACGGTCAGGGTGGATACGGTCAGGGCGGGGCGGGGCAGGGTGGAGCCGCAGCGGCTGCAGCTGCGGCCGCGGGCGGAGCAGGGCAGGGAGGCTATGGTCGGGGTGGAGCCGGACAAGGAGGCGCGGCGGCCGCTACCGGTGCTGGACAAGGTGGCTATGGCGGTCAAGGAGCAGGACAAGGCGGAGCGGGCGCCGCTGCTGCAGCGGCAGCTGCTGGTGGGGCCGGTCAGGGTGGCCAAGGAGGATACGGCAGGGGTGGCTACGGCCAAGGCGGTGCAGGTCAAGGAGGTGCTGGGGCCGCTGCCGCCGCCGCAGCCGCCGGAGGAGCTGGGCAAGGCGGACAAGGAGGGTATGGCCAAGGTGGGTACGGGCAGGGGGGTGCCGGACAAGGCGGAGCGGCCGCAGCGGCTGCAGCCGCCGGTGGTGCTGGCCAGGGTGGCTATGGGAGAGGCGGGGCCGGTCAAGGAGGCGCCGCGGCAGCTGCGGCGGCCGCGGCAGGGGCGGGGCAGGGCGGTTATGGAGGACAAGGCGCGGGTCAGGGTGGAGCGGGTGCGGCGGCGGCAGCGGCAGCTGCGGGCGGCGCTGGGCAAGGAGGTCAAGGCGGGTACGGTCGAGGTGGCTACGGACAGGGAGGGGCTGGACAAGGTGGGGCGGGTGCCGCAGCGGCGGGAGGAGCCGGTCAGGGTGGGCAAGGCGGCTACGGTCAGGGTGGATATGGCCAAGGTGGTGCAGGGCAGGGTGGGGCTGCAGCTGCTGCCGCAGCCGCAGCCGCAGGCGGCGCGGGCCAAGGAGGATACGGCGGATATGGTCAGCAAGGCGGAGCTGGAGCGGCTGCTGCAGCGGCTTCAGGACCGGGCCAGATCTACTACGGTCCGCAGTCCGTTGCCGCCCCAGCTGCTGCTGCGGCGTCTGCGTTGGCAGCACCGGCCACTTCTGCACGCATTTCGTCGCACGCGTCCGCATTGCTTAGTAATGGGCCGACAAATCCTGCGTCCATCTCCAACGTAATCAGTAACGCAGTAAGCCAAATTTCTAGCTCTAACCCAGGCGCTTCTGCCTGTGATGTGCTAGTTCAGGCCCTACTTGAGCTCGTAACGGCGCTATTGACTATTATCGGCTCATCAAATATTGGTAGTGTGAACTATGACAGTTCGGGCCAGTACGCACAAGTCGTTACTCAGTCAGTTCAAAACGCCTTCGCC

Amino Acid Sequence:

GGAGQGGQGGYGGGGYGQGGAGQGGAGAAAAAAAAGGAGQGGQGGYGQGGYGQGGAGQGGAAAAAAAAAGGAGQGGYGRGGAGQGGAAAATGAGQGGYGGQGAGQGGAGAAAAAAAAGGAGQGGQGGYGRGGYGQGGAGQGGAGAAAAAAAAGGAGQGGQGGYGQGGYGQGGAGQGGAAAAAAAAGGAGQGGYGRGGAGQGGAAAAAAAAAGAGQGGYGGQGAGQGGAGAAAAAAAAGGAGQGGQGGYGRGGYGQGGAGQGGAGAAAAGGAGQGGQGGYGQGGYGQGGAGQGGAAAAAAAAAAGGAGQGGYGGYGQQGGAGAAAAAASGPGQIYYGPQSVAAPAAAAASALAAPATSARISSHASALLSNGPTNPASISNVISNAVSQISSSNPGASACDVLVQALLELVTALLTIIGSSNIGSVNYDSSGQYAQVVTQSVQNAFA

**16B LHF1:**

Synthetic DNA Sequence:

GGAGGTGCTGGGCGAGGAGGGCAGGGTGGGTATGGTAGAGGCGGGTACGGGCAGGGAGGCGCCGGACAGGGAGGGGCTGGTGCCGCTGCGGCTGCGGCAGCCGCCGGAGGAGCGGGTCAGGGTGGCCAGGGCGGCTACGGTCAGGGCGGGTATGGGCAGGGCGGGGCTGGACAAGGTGGCGCCGCGGCAGCTGCAGCAGCTGCGGTGGGAGGGGCGGGTCAGGGTGGATACGGCCGAGGTGGGGCAGGTCAAGGCGGTGCTGCGGCCGCGGCGGCCGCTGCAGCCGCTGGTTCTGGTCAGGGAGGTTATGGAGGCCAAGGAGCAGGCCAAGGCGGCGCCGGGGCCGCTGCCGCCGCCGCTGCTGCTGGCGGAGCAGGGCAGGGTGGACAGGGCGGTTACGGTGGTGGAGGATACGGACAAGGAGGTGCCGGCCAAGGCGGAGCTGGAGCTGCTGCGGCAGCAGCAGCCGCGGGAGGCGCTGGACAGGGTGGGCAGGGCGGCTACGGTCAGGGTGGATACGGTCAGGGCGGGGCGGGGCAGGGTGGAGCCGCAGCGGCTGCAGCTGCGGCCGCGGGCGGAGCAGGGCAGGGAGGCTATGGTCGGGGTGGAGCCGGACAAGGAGGCGCGGCGGCCGCTACCGGTGCTGGACAAGGTGGCTATGGCGGTCAAGGAGCAGGACAAGGCGGAGCGGGCGCCGCTGCTGCAGCGGCAGCTGCTGGTGGGGCCGGTCAGGGTGGCCAAGGAGGATACGGCAGGGGTGGCTACGGCCAAGGCGGTGCAGGTCAAGGAGGTGCTGGGGCCGCTGCCGCCGCCGCAGCCGCCGGAGGAGCTGGGCAAGGCGGACAAGGAGGGTATGGCCAAGGTGGGTACGGGCAGGGGGGTGCCGGACAAGGCGGAGCGGCCGCAGCGGCTGCAGCCGCCGGTGGTGCTGGCCAGGGTGGCTATGGGAGAGGCGGGGCCGGTCAAGGAGGCGCCGCGGCAGCTGCGGCGGCCGCGGCAGGGGCGGGGCAGGGCGGTTATGGAGGACAAGGCGCGGGTCAGGGTGGAGCGGGTGCGGCGGCGGCAGCGGCAGCTGCGGGCGGCGCTGGGCAAGGAGGTCAAGGCGGGTACGGTCGAGGTGGCTACGGACAGGGAGGGGCTGGACAAGGTGGGGCGGGTGCCGCAGCGGCGGGAGGAGCCGGTCAGGGTGGGCAAGGCGGCTACGGTCAGGGTGGATATGGCCAAGGTGGTGCAGGGCAGGGTGGGGCTGCAGCTGCTGCCGCAGCCGCAGCCGCAGGCGGCGCGGGCCAAGGAGGATACGGCGGATATGGTCAGCAAGGCGGAGCTGGAGCGGCTGCTGCAGCGGCTTCAGGACCGGGCCAGATCTACTACGGTCCGCAGTCCGTTGCCGCCCCAGCTGCTGCTGCGGCGTCTGCGTTGGCAGCACCGGCCACTTCTGCACGCATTTCGTCGCACGCGTCCGCATTGCTTAGTAATGGGCCGACAAATCCTGCGTCCATCTCCAACGTAATCAGTAACGCAGTAAGCCAAATTTCTAGCTCTAACCCAGGCGCTTCTGCCTGTGATGTGCTAGTTCAGGCCCTACTTGAGCTCGTAACGGCGCTATTGACTATTATCGGCTCATCAAATATTGGTAGTGTGAACTATGACAGTTCGGGCCAGTACGCACAAGTCGTTACTCAGTCAGTTCAAAACGCCTTCGCC

Amino Acid Sequence:

GGAGRGGQGGYGRGGYGQGGAGQGGAGAAAAAAAAGGAGQGGQGGYGQGGYGQGGAGQGGAAAAAAAAVGGAGQGGYGRGGAGQGGAAAAAAAAAAGSGQGGYGGQGAGQGGAGAAAAAAAAGGAGQGGQGGYGGGGYGQGGAGQGGAGAAAAAAAAGGAGQGGQGGYGQGGYGQGGAGQGGAAAAAAAAAGGAGQGGYGRGGAGQGGAAAATGAGQGGYGGQGAGQGGAGAAAAAAAAGGAGQGGQGGYGRGGYGQGGAGQGGAGAAAAAAAAGGAGQGGQGGYGQGGYGQGGAGQGGAAAAAAAAGGAGQGGYGRGGAGQGGAAAAAAAAAGAGQGGYGGQGAGQGGAGAAAAAAAAGGAGQGGQGGYGRGGYGQGGAGQGGAGAAAAGGAGQGGQGGYGQGGYGQGGAGQGGAAAAAAAAAAGGAGQGGYGGYGQQGGAGAAAAAASGPGQIYYGPQSVAAPAAAAASALAAPATSARISSHASALLSNGPTNPASISNVISNAVSQISSSNPGASACDVLVQALLELVTALLTIIGSSNIGSVNYDSSGQYAQVVTQSVQNAFA

**20B LHF1:**

Synthetic DNA Sequence:

GGTGGGGCAGGTAGAGGAGGCCAAGGAGGCTACGGAAGAGGCGGATACGGTCAGGGCGGTGCGGGCCAGGGTGGTGCTGGTGCTGCCGCAGCCGCCGCTGCTGCCGGTGGGGCTGGACAGGGTGGACAAGGTGGGTACGGACAAGGTGGCTACGGGCAGGGAGGCGCGGGTCAAGGAGGGGCGGCGGCAGCCGCTGCAGCTGCCGCTGGTGGCGCCGGACAAGGAGGCTATGGCCGTGGTGGGGCGGGGCAAGGCGGCGCGGCGGCCGCGGCGGGTGCGGGGCAGGGCGGCTATGGAGGTCAAGGAGCCGGCCAAGGTGGGGCAGGCGCAGCAGCCGCTGCGGCCGCTGCCGGAGGTGCTGGGCGAGGAGGGCAGGGTGGGTATGGTAGAGGCGGGTACGGGCAGGGAGGCGCCGGACAGGGAGGGGCTGGTGCCGCTGCGGCTGCGGCAGCCGCCGGAGGAGCGGGTCAGGGTGGCCAGGGCGGCTACGGTCAGGGCGGGTATGGGCAGGGCGGGGCTGGACAAGGTGGCGCCGCGGCAGCTGCAGCAGCTGCGGTGGGAGGGGCGGGTCAGGGTGGATACGGCCGAGGTGGGGCAGGTCAAGGCGGTGCTGCGGCCGCGGCGGCCGCTGCAGCCGCTGGTTCTGGTCAGGGAGGTTATGGAGGCCAAGGAGCAGGCCAAGGCGGCGCCGGGGCCGCTGCCGCCGCCGCTGCTGCTGGCGGAGCAGGGCAGGGTGGACAGGGCGGTTACGGTGGTGGAGGATACGGACAAGGAGGTGCCGGCCAAGGCGGAGCTGGAGCTGCTGCGGCAGCAGCAGCCGCGGGAGGCGCTGGACAGGGTGGGCAGGGCGGCTACGGTCAGGGTGGATACGGTCAGGGCGGGGCGGGGCAGGGTGGAGCCGCAGCGGCTGCAGCTGCGGCCGCGGGCGGAGCAGGGCAGGGAGGCTATGGTCGGGGTGGAGCCGGACAAGGAGGCGCGGCGGCCGCTACCGGTGCTGGACAAGGTGGCTATGGCGGTCAAGGAGCAGGACAAGGCGGAGCGGGCGCCGCTGCTGCAGCGGCAGCTGCTGGTGGGGCCGGTCAGGGTGGCCAAGGAGGATACGGCAGGGGTGGCTACGGCCAAGGCGGTGCAGGTCAAGGAGGTGCTGGGGCCGCTGCCGCCGCCGCAGCCGCCGGAGGAGCTGGGCAAGGCGGACAAGGAGGGTATGGCCAAGGTGGGTACGGGCAGGGGGGTGCCGGACAAGGCGGAGCGGCCGCAGCGGCTGCAGCCGCCGGTGGTGCTGGCCAGGGTGGCTATGGGAGAGGCGGGGCCGGTCAAGGAGGCGCCGCGGCAGCTGCGGCGGCCGCGGCAGGGGCGGGGCAGGGCGGTTATGGAGGACAAGGCGCGGGTCAGGGTGGAGCGGGTGCGGCGGCGGCAGCGGCAGCTGCGGGCGGCGCTGGGCAAGGAGGTCAAGGCGGGTACGGTCGAGGTGGCTACGGACAGGGAGGGGCTGGACAAGGTGGGGCGGGTGCCGCAGCGGCGGGAGGAGCCGGTCAGGGTGGGCAAGGCGGCTACGGTCAGGGTGGATATGGCCAAGGTGGTGCAGGGCAGGGTGGGGCTGCAGCTGCTGCCGCAGCCGCAGCCGCAGGCGGCGCGGGCCAAGGAGGATACGGCGGATATGGTCAGCAAGGCGGAGCTGGAGCGGCTGCTGCAGCGGCTTCAGGACCGGGCCAGATCTACTACGGTCCGCAGTCCGTTGCCGCCCCAGCTGCTGCTGCGGCGTCTGCGTTGGCAGCACCGGCCACTTCTGCACGCATTTCGTCGCACGCGTCCGCATTGCTTAGTAATGGGCCGACAAATCCTGCGTCCATCTCCAACGTAATCAGTAACGCAGTAAGCCAAATTTCTAGCTCTAACCCAGGCGCTTCTGCCTGTGATGTGCTAGTTCAGGCCCTACTTGAGCTCGTAACGGCGCTATTGACTATTATCGGCTCATCAAATATTGGTAGTGTGAACTATGACAGTTCGGGCCAGTACGCACAAGTCGTTACTCAGTCAGTTCAAAACGCCTTCGCC

Amino Acid Sequence:

GGAGRGGQGGYGRGGYGQGGAGQGGAGAAAAAAAAGGAGQGGQGGYGQGGYGQGGAGQGGAAAAAAAAAGGAGQGGYGRGGAGQGGAAAAAGAGQGGYGGQGAGQGGAGAAAAAAAAGGAGRGGQGGYGRGGYGQGGAGQGGAGAAAAAAAAGGAGQGGQGGYGQGGYGQGGAGQGGAAAAAAAAVGGAGQGGYGRGGAGQGGAAAAAAAAAAGSGQGGYGGQGAGQGGAGAAAAAAAAGGAGQGGQGGYGGGGYGQGGAGQGGAGAAAAAAAAGGAGQGGQGGYGQGGYGQGGAGQGGAAAAAAAAAGGAGQGGYGRGGAGQGGAAAATGAGQGGYGGQGAGQGGAGAAAAAAAAGGAGQGGQGGYGRGGYGQGGAGQGGAGAAAAAAAAGGAGQGGQGGYGQGGYGQGGAGQGGAAAAAAAAGGAGQGGYGRGGAGQGGAAAAAAAAAGAGQGGYGGQGAGQGGAGAAAAAAAAGGAGQGGQGGYGRGGYGQGGAGQGGAGAAAAGGAGQGGQGGYGQGGYGQGGAGQGGAAAAAAAAAAGGAGQGGYGGYGQQGGAGAAAAAASGPGQIYYGPQSVAAPAAAAASALAAPATSARISSHASALLSNGPTNPASISNVISNAVSQISSSNPGASACDVLVQALLELVTALLTIIGSSNIGSVNYDSSGQYAQVVTQSVQNAFA

**24B LHF1:**

Synthetic DNA Sequence:

GCGGGCGGAGCAGGGCAGGGCGGCCAGGGTGGATACGGGAGAGGTGGCTACGGTCAAGGAGGGGCCGGCCAAGGTGGTGCGGGAGCAGCCGCTGCAGCTGCGGCCGCTGGTGGGGCTTCGCAGGGTGGCCAGGGTGGTTACGGGCAAGGTGATTACGGGCAGGGAGGAGCGGGACAAGGTGGGGCCGCCGCTGCGGCTGCGGCAGCAGGTGGCGCTGGACAAGGAGGGTATGGACGAGGAGGGGCAGGCCAAGGCGGGGCTGCCGCTGCTGCAGGGGCAGGTCAAGGCGGGTACGGCGGACAAGGTGCAGGGCAAGGAGGTGCTGGAGCTGCGGCTGCAGCAGCAGCTGCGGGTGGGGCAGGTAGAGGAGGCCAAGGAGGCTACGGAAGAGGCGGATACGGTCAGGGCGGTGCGGGCCAGGGTGGTGCTGGTGCTGCCGCAGCCGCCGCTGCTGCCGGTGGGGCTGGACAGGGTGGACAAGGTGGGTACGGACAAGGTGGCTACGGGCAGGGAGGCGCGGGTCAAGGAGGGGCGGCGGCAGCCGCTGCAGCTGCCGCTGGTGGCGCCGGACAAGGAGGCTATGGCCGTGGTGGGGCGGGGCAAGGCGGCGCGGCGGCCGCGGCGGGTGCGGGGCAGGGCGGCTATGGAGGTCAAGGAGCCGGCCAAGGTGGGGCAGGCGCAGCAGCCGCTGCGGCCGCTGCCGGAGGTGCTGGGCGAGGAGGGCAGGGTGGGTATGGTAGAGGCGGGTACGGGCAGGGAGGCGCCGGACAGGGAGGGGCTGGTGCCGCTGCGGCTGCGGCAGCCGCCGGAGGAGCGGGTCAGGGTGGCCAGGGCGGCTACGGTCAGGGCGGGTATGGGCAGGGCGGGGCTGGACAAGGTGGCGCCGCGGCAGCTGCAGCAGCTGCGGTGGGAGGGGCGGGTCAGGGTGGATACGGCCGAGGTGGGGCAGGTCAAGGCGGTGCTGCGGCCGCGGCGGCCGCTGCAGCCGCTGGTTCTGGTCAGGGAGGTTATGGAGGCCAAGGAGCAGGCCAAGGCGGCGCCGGGGCCGCTGCCGCCGCCGCTGCTGCTGGCGGAGCAGGGCAGGGTGGACAGGGCGGTTACGGTGGTGGAGGATACGGACAAGGAGGTGCCGGCCAAGGCGGAGCTGGAGCTGCTGCGGCAGCAGCAGCCGCGGGAGGCGCTGGACAGGGTGGGCAGGGCGGCTACGGTCAGGGTGGATACGGTCAGGGCGGGGCGGGGCAGGGTGGAGCCGCAGCGGCTGCAGCTGCGGCCGCGGGCGGAGCAGGGCAGGGAGGCTATGGTCGGGGTGGAGCCGGACAAGGAGGCGCGGCGGCCGCTACCGGTGCTGGACAAGGTGGCTATGGCGGTCAAGGAGCAGGACAAGGCGGAGCGGGCGCCGCTGCTGCAGCGGCAGCTGCTGGTGGGGCCGGTCAGGGTGGCCAAGGAGGATACGGCAGGGGTGGCTACGGCCAAGGCGGTGCAGGTCAAGGAGGTGCTGGGGCCGCTGCCGCCGCCGCAGCCGCCGGAGGAGCTGGGCAAGGCGGACAAGGAGGGTATGGCCAAGGTGGGTACGGGCAGGGGGGTGCCGGACAAGGCGGAGCGGCCGCAGCGGCTGCAGCCGCCGGTGGTGCTGGCCAGGGTGGCTATGGGAGAGGCGGGGCCGGTCAAGGAGGCGCCGCGGCAGCTGCGGCGGCCGCGGCAGGGGCGGGGCAGGGCGGTTATGGAGGACAAGGCGCGGGTCAGGGTGGAGCGGGTGCGGCGGCGGCAGCGGCAGCTGCGGGCGGCGCTGGGCAAGGAGGTCAAGGCGGGTACGGTCGAGGTGGCTACGGACAGGGAGGGGCTGGACAAGGTGGGGCGGGTGCCGCAGCGGCGGGAGGAGCCGGTCAGGGTGGGCAAGGCGGCTACGGTCAGGGTGGATATGGCCAAGGTGGTGCAGGGCAGGGTGGGGCTGCAGCTGCTGCCGCAGCCGCAGCCGCAGGCGGCGCGGGCCAAGGAGGATACGGCGGATATGGTCAGCAAGGCGGAGCTGGAGCGGCTGCTGCAGCGGCTTCAGGACCGGGCCAGATCTACTACGGTCCGCAGTCCGTTGCCGCCCCAGCTGCTGCTGCGGCGTCTGCGTTGGCAGCACCGGCCACTTCTGCACGCATTTCGTCGCACGCGTCCGCATTGCTTAGTAATGGGCCGACAAATCCTGCGTCCATCTCCAACGTAATCAGTAACGCAGTAAGCCAAATTTCTAGCTCTAACCCAGGCGCTTCTGCCTGTGATGTGCTAGTTCAGGCCCTACTTGAGCTCGTAACGGCGCTATTGACTATTATCGGCTCATCAAATATTGGTAGTGTGAACTATGACAGTTCGGGCCAGTACGCACAAGTCGTTACTCAGTCAGTTCAAAACGCCTTCGCC

Amino Acid Sequence:

AGGAGQGGQGGYGRGGYGQGGAGQGGAGAAAAAAAAGGASQGGQGGYGQGDYGQGGAGQGGAAAAAAAAGGAGQGGYGRGGAGQGGAAAAAGAGQGGYGGQGAGQGGAGAAAAAAAAGGAGRGGQGGYGRGGYGQGGAGQGGAGAAAAAAAAGGAGQGGQGGYGQGGYGQGGAGQGGAAAAAAAAAGGAGQGGYGRGGAGQGGAAAAAGAGQGGYGGQGAGQGGAGAAAAAAAAGGAGRGGQGGYGRGGYGQGGAGQGGAGAAAAAAAAGGAGQGGQGGYGQGGYGQGGAGQGGAAAAAAAAVGGAGQGGYGRGGAGQGGAAAAAAAAAAGSGQGGYGGQGAGQGGAGAAAAAAAAGGAGQGGQGGYGGGGYGQGGAGQGGAGAAAAAAAAGGAGQGGQGGYGQGGYGQGGAGQGGAAAAAAAAAGGAGQGGYGRGGAGQGGAAAATGAGQGGYGGQGAGQGGAGAAAAAAAAGGAGQGGQGGYGRGGYGQGGAGQGGAGAAAAAAAAGGAGQGGQGGYGQGGYGQGGAGQGGAAAAAAAAGGAGQGGYGRGGAGQGGAAAAAAAAAGAGQGGYGGQGAGQGGAGAAAAAAAAGGAGQGGQGGYGRGGYGQGGAGQGGAGAAAAGGAGQGGQGGYGQGGYGQGGAGQGGAAAAAAAAAAGGAGQGGYGGYGQQGGAGAAAAAASGPGQIYYGPQSVAAPAAAAASALAAPATSARISSHASALLSNGPTNPASISNVISNAVSQISSSNPGASACDVLVQALLELVTALLTIIGSSNIGSVNYDSSGQYAQVVTQSVQNAFA

**28B LHF1:**

Synthetic DNA Sequence:

GGAGGCGCGGGGCAGGGAGGCCAAGGCGGGTATGGAAGAGGAGGCTATGGGCAAGGCGGTGCGGGACAAGGTGGGGCCGGTGCTGCGGCGGCTGCAGCGGCTGCTGGAGGTGCTGGCCAAGGAGGGCAAGGCGGATACGGTCAGGGTGGAAATGGGCAAGGCGGAGCGGGCCAGGGCGGCGCGGCCGCTGCCGCGGCCGCAGCAGGCGGCGCGGGCCAAGGCGGGTACGGTCGTGGCGGGGCCGGACAAGGCGGAGCTGCCGCCGCCGCTGCGGCCGCGGCTGGGGCTGGGCAGGGTGGATATGGAGGGCAAGGCGCGGGACAGGGTGGTGCAGGCGCGGCTGCCGCTGCTGCTGCGGCGGGCGGAGCAGGGCAGGGCGGCCAGGGTGGATACGGGAGAGGTGGCTACGGTCAAGGAGGGGCCGGCCAAGGTGGTGCGGGAGCAGCCGCTGCAGCTGCGGCCGCTGGTGGGGCTTCGCAGGGTGGCCAGGGTGGTTACGGGCAAGGTGATTACGGGCAGGGAGGAGCGGGACAAGGTGGGGCCGCCGCTGCGGCTGCGGCAGCAGGTGGCGCTGGACAAGGAGGGTATGGACGAGGAGGGGCAGGCCAAGGCGGGGCTGCCGCTGCTGCAGGGGCAGGTCAAGGCGGGTACGGCGGACAAGGTGCAGGGCAAGGAGGTGCTGGAGCTGCGGCTGCAGCAGCAGCTGCGGGTGGGGCAGGTAGAGGAGGCCAAGGAGGCTACGGAAGAGGCGGATACGGTCAGGGCGGTGCGGGCCAGGGTGGTGCTGGTGCTGCCGCAGCCGCCGCTGCTGCCGGTGGGGCTGGACAGGGTGGACAAGGTGGGTACGGACAAGGTGGCTACGGGCAGGGAGGCGCGGGTCAAGGAGGGGCGGCGGCAGCCGCTGCAGCTGCCGCTGGTGGCGCCGGACAAGGAGGCTATGGCCGTGGTGGGGCGGGGCAAGGCGGCGCGGCGGCCGCGGCGGGTGCGGGGCAGGGCGGCTATGGAGGTCAAGGAGCCGGCCAAGGTGGGGCAGGCGCAGCAGCCGCTGCGGCCGCTGCCGGAGGTGCTGGGCGAGGAGGGCAGGGTGGGTATGGTAGAGGCGGGTACGGGCAGGGAGGCGCCGGACAGGGAGGGGCTGGTGCCGCTGCGGCTGCGGCAGCCGCCGGAGGAGCGGGTCAGGGTGGCCAGGGCGGCTACGGTCAGGGCGGGTATGGGCAGGGCGGGGCTGGACAAGGTGGCGCCGCGGCAGCTGCAGCAGCTGCGGTGGGAGGGGCGGGTCAGGGTGGATACGGCCGAGGTGGGGCAGGTCAAGGCGGTGCTGCGGCCGCGGCGGCCGCTGCAGCCGCTGGTTCTGGTCAGGGAGGTTATGGAGGCCAAGGAGCAGGCCAAGGCGGCGCCGGGGCCGCTGCCGCCGCCGCTGCTGCTGGCGGAGCAGGGCAGGGTGGACAGGGCGGTTACGGTGGTGGAGGATACGGACAAGGAGGTGCCGGCCAAGGCGGAGCTGGAGCTGCTGCGGCAGCAGCAGCCGCGGGAGGCGCTGGACAGGGTGGGCAGGGCGGCTACGGTCAGGGTGGATACGGTCAGGGCGGGGCGGGGCAGGGTGGAGCCGCAGCGGCTGCAGCTGCGGCCGCGGGCGGAGCAGGGCAGGGAGGCTATGGTCGGGGTGGAGCCGGACAAGGAGGCGCGGCGGCCGCTACCGGTGCTGGACAAGGTGGCTATGGCGGTCAAGGAGCAGGACAAGGCGGAGCGGGCGCCGCTGCTGCAGCGGCAGCTGCTGGTGGGGCCGGTCAGGGTGGCCAAGGAGGATACGGCAGGGGTGGCTACGGCCAAGGCGGTGCAGGTCAAGGAGGTGCTGGGGCCGCTGCCGCCGCCGCAGCCGCCGGAGGAGCTGGGCAAGGCGGACAAGGAGGGTATGGCCAAGGTGGGTACGGGCAGGGGGGTGCCGGACAAGGCGGAGCGGCCGCAGCGGCTGCAGCCGCCGGTGGTGCTGGCCAGGGTGGCTATGGGAGAGGCGGGGCCGGTCAAGGAGGCGCCGCGGCAGCTGCGGCGGCCGCGGCAGGGGCGGGGCAGGGCGGTTATGGAGGACAAGGCGCGGGTCAGGGTGGAGCGGGTGCGGCGGCGGCAGCGGCAGCTGCGGGCGGCGCTGGGCAAGGAGGTCAAGGCGGGTACGGTCGAGGTGGCTACGGACAGGGAGGGGCTGGACAAGGTGGGGCGGGTGCCGCAGCGGCGGGAGGAGCCGGTCAGGGTGGGCAAGGCGGCTACGGTCAGGGTGGATATGGCCAAGGTGGTGCAGGGCAGGGTGGGGCTGCAGCTGCTGCCGCAGCCGCAGCCGCAGGCGGCGCGGGCCAAGGAGGATACGGCGGATATGGTCAGCAAGGCGGAGCTGGAGCGGCTGCTGCAGCGGCTTCAGGACCGGGCCAGATCTACTACGGTCCGCAGTCCGTTGCCGCCCCAGCTGCTGCTGCGGCGTCTGCGTTGGCAGCACCGGCCACTTCTGCACGCATTTCGTCGCACGCGTCCGCATTGCTTAGTAATGGGCCGACAAATCCTGCGTCCATCTCCAACGTAATCAGTAACGCAGTAAGCCAAATTTCTAGCTCTAACCCAGGCGCTTCTGCCTGTGATGTGCTAGTTCAGGCCCTACTTGAGCTCGTAACGGCGCTATTGACTATTATCGGCTCATCAAATATTGGTAGTGTGAACTATGACAGTTCGGGCCAGTACGCACAAGTCGTTACTCAGTCAGTTCAAAACGCCTTCGCC

Amino Acid Sequence:

GGAGQGGQGGYGRGGYGQGGAGQGGAGAAAAAAAAGGAGQGGQGGYGQGGNGQGGAGQGGAAAAAAAAGGAGQGGYGRGGAGQGGAAAAAAAAAGAGQGGYGGQGAGQGGAGAAAAAAAAGGAGQGGQGGYGRGGYGQGGAGQGGAGAAAAAAAAGGASQGGQGGYGQGDYGQGGAGQGGAAAAAAAAGGAGQGGYGRGGAGQGGAAAAAGAGQGGYGGQGAGQGGAGAAAAAAAAGGAGRGGQGGYGRGGYGQGGAGQGGAGAAAAAAAAGGAGQGGQGGYGQGGYGQGGAGQGGAAAAAAAAAGGAGQGGYGRGGAGQGGAAAAAGAGQGGYGGQGAGQGGAGAAAAAAAAGGAGRGGQGGYGRGGYGQGGAGQGGAGAAAAAAAAGGAGQGGQGGYGQGGYGQGGAGQGGAAAAAAAAVGGAGQGGYGRGGAGQGGAAAAAAAAAAGSGQGGYGGQGAGQGGAGAAAAAAAAGGAGQGGQGGYGGGGYGQGGAGQGGAGAAAAAAAAGGAGQGGQGGYGQGGYGQGGAGQGGAAAAAAAAAGGAGQGGYGRGGAGQGGAAAATGAGQGGYGGQGAGQGGAGAAAAAAAAGGAGQGGQGGYGRGGYGQGGAGQGGAGAAAAAAAAGGAGQGGQGGYGQGGYGQGGAGQGGAAAAAAAAGGAGQGGYGRGGAGQGGAAAAAAAAAGAGQGGYGGQGAGQGGAGAAAAAAAAGGAGQGGQGGYGRGGYGQGGAGQGGAGAAAAGGAGQGGQGGYGQGGYGQGGAGQGGAAAAAAAAAAGGAGQGGYGGYGQQGGAGAAAAAASGPGQIYYGPQSVAAPAAAAASALAAPATSARISSHASALLSNGPTNPASISNVISNAVSQISSSNPGASACDVLVQALLELVTALLTIIGSSNIGSVNYDSSGQYAQVVTQSVQNAFA

**32B LHF1:**

Synthetic DNA Sequence:

GGAGGCGCCGGGCAGGGAGGGCAAGGAGGTTACGGGCGTGGCGGCTACGGACAAGGTGGCGCGGGCCAGGGTGGGGCTGGCGCAGCGGCGGCGGCTGCCGCCGCAGGCGGCGCTGGGCAAGGCGGGCAGGGTGGATACGGGCAGGGCGGATATGGCCAGGGTGGGTACGGTCAAGGAGGTGCCGGACAGGGAGGAGCAGCGGCAGCAGCTGCGGCGGCGGCAGCTGGAGGGGCAGGGCAGGGCGGTTATGGTCGTGGAGGGGCCGGACAAGGAGGTGCAGCTGCAGCAGCGGGAGCTGGGCAGGGTGGATACGGAGGTCAGGGCGCAGGACAGGGTGGTGCTGGCGCTGCGGCTGCGGCAGCCGCGGCGGGAGGCGCGGGGCAGGGAGGCCAAGGCGGGTATGGAAGAGGAGGCTATGGGCAAGGCGGTGCGGGACAAGGTGGGGCCGGTGCTGCGGCGGCTGCAGCGGCTGCTGGAGGTGCTGGCCAAGGAGGGCAAGGCGGATACGGTCAGGGTGGAAATGGGCAAGGCGGAGCGGGCCAGGGCGGCGCGGCCGCTGCCGCGGCCGCAGCAGGCGGCGCGGGCCAAGGCGGGTACGGTCGTGGCGGGGCCGGACAAGGCGGAGCTGCCGCCGCCGCTGCGGCCGCGGCTGGGGCTGGGCAGGGTGGATATGGAGGGCAAGGCGCGGGACAGGGTGGTGCAGGCGCGGCTGCCGCTGCTGCTGCGGCGGGCGGAGCAGGGCAGGGCGGCCAGGGTGGATACGGGAGAGGTGGCTACGGTCAAGGAGGGGCCGGCCAAGGTGGTGCGGGAGCAGCCGCTGCAGCTGCGGCCGCTGGTGGGGCTTCGCAGGGTGGCCAGGGTGGTTACGGGCAAGGTGATTACGGGCAGGGAGGAGCGGGACAAGGTGGGGCCGCCGCTGCGGCTGCGGCAGCAGGTGGCGCTGGACAAGGAGGGTATGGACGAGGAGGGGCAGGCCAAGGCGGGGCTGCCGCTGCTGCAGGGGCAGGTCAAGGCGGGTACGGCGGACAAGGTGCAGGGCAAGGAGGTGCTGGAGCTGCGGCTGCAGCAGCAGCTGCGGGTGGGGCAGGTAGAGGAGGCCAAGGAGGCTACGGAAGAGGCGGATACGGTCAGGGCGGTGCGGGCCAGGGTGGTGCTGGTGCTGCCGCAGCCGCCGCTGCTGCCGGTGGGGCTGGACAGGGTGGACAAGGTGGGTACGGACAAGGTGGCTACGGGCAGGGAGGCGCGGGTCAAGGAGGGGCGGCGGCAGCCGCTGCAGCTGCCGCTGGTGGCGCCGGACAAGGAGGCTATGGCCGTGGTGGGGCGGGGCAAGGCGGCGCGGCGGCCGCGGCGGGTGCGGGGCAGGGCGGCTATGGAGGTCAAGGAGCCGGCCAAGGTGGGGCAGGCGCAGCAGCCGCTGCGGCCGCTGCCGGAGGTGCTGGGCGAGGAGGGCAGGGTGGGTATGGTAGAGGCGGGTACGGGCAGGGAGGCGCCGGACAGGGAGGGGCTGGTGCCGCTGCGGCTGCGGCAGCCGCCGGAGGAGCGGGTCAGGGTGGCCAGGGCGGCTACGGTCAGGGCGGGTATGGGCAGGGCGGGGCTGGACAAGGTGGCGCCGCGGCAGCTGCAGCAGCTGCGGTGGGAGGGGCGGGTCAGGGTGGATACGGCCGAGGTGGGGCAGGTCAAGGCGGTGCTGCGGCCGCGGCGGCCGCTGCAGCCGCTGGTTCTGGTCAGGGAGGTTATGGAGGCCAAGGAGCAGGCCAAGGCGGCGCCGGGGCCGCTGCCGCCGCCGCTGCTGCTGGCGGAGCAGGGCAGGGTGGACAGGGCGGTTACGGTGGTGGAGGATACGGACAAGGAGGTGCCGGCCAAGGCGGAGCTGGAGCTGCTGCGGCAGCAGCAGCCGCGGGAGGCGCTGGACAGGGTGGGCAGGGCGGCTACGGTCAGGGTGGATACGGTCAGGGCGGGGCGGGGCAGGGTGGAGCCGCAGCGGCTGCAGCTGCGGCCGCGGGCGGAGCAGGGCAGGGAGGCTATGGTCGGGGTGGAGCCGGACAAGGAGGCGCGGCGGCCGCTACCGGTGCTGGACAAGGTGGCTATGGCGGTCAAGGAGCAGGACAAGGCGGAGCGGGCGCCGCTGCTGCAGCGGCAGCTGCTGGTGGGGCCGGTCAGGGTGGCCAAGGAGGATACGGCAGGGGTGGCTACGGCCAAGGCGGTGCAGGTCAAGGAGGTGCTGGGGCCGCTGCCGCCGCCGCAGCCGCCGGAGGAGCTGGGCAAGGCGGACAAGGAGGGTATGGCCAAGGTGGGTACGGGCAGGGGGGTGCCGGACAAGGCGGAGCGGCCGCAGCGGCTGCAGCCGCCGGTGGTGCTGGCCAGGGTGGCTATGGGAGAGGCGGGGCCGGTCAAGGAGGCGCCGCGGCAGCTGCGGCGGCCGCGGCAGGGGCGGGGCAGGGCGGTTATGGAGGACAAGGCGCGGGTCAGGGTGGAGCGGGTGCGGCGGCGGCAGCGGCAGCTGCGGGCGGCGCTGGGCAAGGAGGTCAAGGCGGGTACGGTCGAGGTGGCTACGGACAGGGAGGGGCTGGACAAGGTGGGGCGGGTGCCGCAGCGGCGGGAGGAGCCGGTCAGGGTGGGCAAGGCGGCTACGGTCAGGGTGGATATGGCCAAGGTGGTGCAGGGCAGGGTGGGGCTGCAGCTGCTGCCGCAGCCGCAGCCGCAGGCGGCGCGGGCCAAGGAGGATACGGCGGATATGGTCAGCAAGGCGGAGCTGGAGCGGCTGCTGCAGCGGCTTCAGGACCGGGCCAGATCTACTACGGTCCGCAGTCCGTTGCCGCCCCAGCTGCTGCTGCGGCGTCTGCGTTGGCAGCACCGGCCACTTCTGCACGCATTTCGTCGCACGCGTCCGCATTGCTTAGTAATGGGCCGACAAATCCTGCGTCCATCTCCAACGTAATCAGTAACGCAGTAAGCCAAATTTCTAGCTCTAACCCAGGCGCTTCTGCCTGTGATGTGCTAGTTCAGGCCCTACTTGAGCTCGTAACGGCGCTATTGACTATTATCGGCTCATCAAATATTGGTAGTGTGAACTATGACAGTTCGGGCCAGTACGCACAAGTCGTTACTCAGTCAGTTCAAAACGCCTTCGCC

Amino Acid Sequence:

GGAGQGGQGGYGRGGYGQGGAGQGGAGAAAAAAAAGGAGQGGQGGYGQGGYGQGGYGQGGAGQGGAAAAAAAAAAGGAGQGGYGRGGAGQGGAAAAAGAGQGGYGGQGAGQGGAGAAAAAAAAGGAGQGGQGGYGRGGYGQGGAGQGGAGAAAAAAAAGGAGQGGQGGYGQGGNGQGGAGQGGAAAAAAAAGGAGQGGYGRGGAGQGGAAAAAAAAAGAGQGGYGGQGAGQGGAGAAAAAAAAGGAGQGGQGGYGRGGYGQGGAGQGGAGAAAAAAAAGGASQGGQGGYGQGDYGQGGAGQGGAAAAAAAAGGAGQGGYGRGGAGQGGAAAAAGAGQGGYGGQGAGQGGAGAAAAAAAAGGAGRGGQGGYGRGGYGQGGAGQGGAGAAAAAAAAGGAGQGGQGGYGQGGYGQGGAGQGGAAAAAAAAAGGAGQGGYGRGGAGQGGAAAAAGAGQGGYGGQGAGQGGAGAAAAAAAAGGAGRGGQGGYGRGGYGQGGAGQGGAGAAAAAAAAGGAGQGGQGGYGQGGYGQGGAGQGGAAAAAAAAVGGAGQGGYGRGGAGQGGAAAAAAAAAAGSGQGGYGGQGAGQGGAGAAAAAAAAGGAGQGGQGGYGGGGYGQGGAGQGGAGAAAAAAAAGGAGQGGQGGYGQGGYGQGGAGQGGAAAAAAAAAGGAGQGGYGRGGAGQGGAAAATGAGQGGYGGQGAGQGGAGAAAAAAAAGGAGQGGQGGYGRGGYGQGGAGQGGAGAAAAAAAAGGAGQGGQGGYGQGGYGQGGAGQGGAAAAAAAAGGAGQGGYGRGGAGQGGAAAAAAAAAGAGQGGYGGQGAGQGGAGAAAAAAAAGGAGQGGQGGYGRGGYGQGGAGQGGAGAAAAGGAGQGGQGGYGQGGYGQGGAGQGGAAAAAAAAAAGGAGQGGYGGYGQQGGAGAAAAAASGPGQIYYGPQSVAAPAAAAASALAAPATSARISSHASALLSNGPTNPASISNVISNAVSQISSSNPGASACDVLVQALLELVTALLTIIGSSNIGSVNYDSSGQYAQVVTQSVQNAFA

**36B LHF1:**

Synthetic DNA Sequence:

GGAGGAGCAGGGCAGGGCGGACAAGGTGGCTATGGCAGGGGCGGTTATGGACAGGGCGGCGCAGGACAAGGAGGAGCGGGCGCGGCGGCTGCAGCAGCCGCTGCCGGAGGCGCCGGTCAGGGTGGCCAGGGCGGGTACGGCCAAGGCGGATACGGTCAAGGCGGGGCAGGACAGGGTGGCGCTGCGGCTGCGGCAGCAGCAGCGGCAGGAGGGGCGGGCCAAGGCGGATATGGGCGGGGCGGAGCGGGACAAGGCGGAGCGGCAGCTGCAGCGGCCGCAGCGGCGGGTTCAGGCCAGGGAGGTTACGGCGGCCAAGGTGCCGGACAGGGAGGAGCAGGCGCCGCGGCCGCCGCAGCAGCTGCGGGAGGCGCCGGGCAGGGAGGGCAAGGAGGTTACGGGCGTGGCGGCTACGGACAAGGTGGCGCGGGCCAGGGTGGGGCTGGCGCAGCGGCGGCGGCTGCCGCCGCAGGCGGCGCTGGGCAAGGCGGGCAGGGTGGATACGGGCAGGGCGGATATGGCCAGGGTGGGTACGGTCAAGGAGGTGCCGGACAGGGAGGAGCAGCGGCAGCAGCTGCGGCGGCGGCAGCTGGAGGGGCAGGGCAGGGCGGTTATGGTCGTGGAGGGGCCGGACAAGGAGGTGCAGCTGCAGCAGCGGGAGCTGGGCAGGGTGGATACGGAGGTCAGGGCGCAGGACAGGGTGGTGCTGGCGCTGCGGCTGCGGCAGCCGCGGCGGGAGGCGCGGGGCAGGGAGGCCAAGGCGGGTATGGAAGAGGAGGCTATGGGCAAGGCGGTGCGGGACAAGGTGGGGCCGGTGCTGCGGCGGCTGCAGCGGCTGCTGGAGGTGCTGGCCAAGGAGGGCAAGGCGGATACGGTCAGGGTGGAAATGGGCAAGGCGGAGCGGGCCAGGGCGGCGCGGCCGCTGCCGCGGCCGCAGCAGGCGGCGCGGGCCAAGGCGGGTACGGTCGTGGCGGGGCCGGACAAGGCGGAGCTGCCGCCGCCGCTGCGGCCGCGGCTGGGGCTGGGCAGGGTGGATATGGAGGGCAAGGCGCGGGACAGGGTGGTGCAGGCGCGGCTGCCGCTGCTGCTGCGGCGGGCGGAGCAGGGCAGGGCGGCCAGGGTGGATACGGGAGAGGTGGCTACGGTCAAGGAGGGGCCGGCCAAGGTGGTGCGGGAGCAGCCGCTGCAGCTGCGGCCGCTGGTGGGGCTTCGCAGGGTGGCCAGGGTGGTTACGGGCAAGGTGATTACGGGCAGGGAGGAGCGGGACAAGGTGGGGCCGCCGCTGCGGCTGCGGCAGCAGGTGGCGCTGGACAAGGAGGGTATGGACGAGGAGGGGCAGGCCAAGGCGGGGCTGCCGCTGCTGCAGGGGCAGGTCAAGGCGGGTACGGCGGACAAGGTGCAGGGCAAGGAGGTGCTGGAGCTGCGGCTGCAGCAGCAGCTGCGGGTGGGGCAGGTAGAGGAGGCCAAGGAGGCTACGGAAGAGGCGGATACGGTCAGGGCGGTGCGGGCCAGGGTGGTGCTGGTGCTGCCGCAGCCGCCGCTGCTGCCGGTGGGGCTGGACAGGGTGGACAAGGTGGGTACGGACAAGGTGGCTACGGGCAGGGAGGCGCGGGTCAAGGAGGGGCGGCGGCAGCCGCTGCAGCTGCCGCTGGTGGCGCCGGACAAGGAGGCTATGGCCGTGGTGGGGCGGGGCAAGGCGGCGCGGCGGCCGCGGCGGGTGCGGGGCAGGGCGGCTATGGAGGTCAAGGAGCCGGCCAAGGTGGGGCAGGCGCAGCAGCCGCTGCGGCCGCTGCCGGAGGTGCTGGGCGAGGAGGGCAGGGTGGGTATGGTAGAGGCGGGTACGGGCAGGGAGGCGCCGGACAGGGAGGGGCTGGTGCCGCTGCGGCTGCGGCAGCCGCCGGAGGAGCGGGTCAGGGTGGCCAGGGCGGCTACGGTCAGGGCGGGTATGGGCAGGGCGGGGCTGGACAAGGTGGCGCCGCGGCAGCTGCAGCAGCTGCGGTGGGAGGGGCGGGTCAGGGTGGATACGGCCGAGGTGGGGCAGGTCAAGGCGGTGCTGCGGCCGCGGCGGCCGCTGCAGCCGCTGGTTCTGGTCAGGGAGGTTATGGAGGCCAAGGAGCAGGCCAAGGCGGCGCCGGGGCCGCTGCCGCCGCCGCTGCTGCTGGCGGAGCAGGGCAGGGTGGACAGGGCGGTTACGGTGGTGGAGGATACGGACAAGGAGGTGCCGGCCAAGGCGGAGCTGGAGCTGCTGCGGCAGCAGCAGCCGCGGGAGGCGCTGGACAGGGTGGGCAGGGCGGCTACGGTCAGGGTGGATACGGTCAGGGCGGGGCGGGGCAGGGTGGAGCCGCAGCGGCTGCAGCTGCGGCCGCGGGCGGAGCAGGGCAGGGAGGCTATGGTCGGGGTGGAGCCGGACAAGGAGGCGCGGCGGCCGCTACCGGTGCTGGACAAGGTGGCTATGGCGGTCAAGGAGCAGGACAAGGCGGAGCGGGCGCCGCTGCTGCAGCGGCAGCTGCTGGTGGGGCCGGTCAGGGTGGCCAAGGAGGATACGGCAGGGGTGGCTACGGCCAAGGCGGTGCAGGTCAAGGAGGTGCTGGGGCCGCTGCCGCCGCCGCAGCCGCCGGAGGAGCTGGGCAAGGCGGACAAGGAGGGTATGGCCAAGGTGGGTACGGGCAGGGGGGTGCCGGACAAGGCGGAGCGGCCGCAGCGGCTGCAGCCGCCGGTGGTGCTGGCCAGGGTGGCTATGGGAGAGGCGGGGCCGGTCAAGGAGGCGCCGCGGCAGCTGCGGCGGCCGCGGCAGGGGCGGGGCAGGGCGGTTATGGAGGACAAGGCGCGGGTCAGGGTGGAGCGGGTGCGGCGGCGGCAGCGGCAGCTGCGGGCGGCGCTGGGCAAGGAGGTCAAGGCGGGTACGGTCGAGGTGGCTACGGACAGGGAGGGGCTGGACAAGGTGGGGCGGGTGCCGCAGCGGCGGGAGGAGCCGGTCAGGGTGGGCAAGGCGGCTACGGTCAGGGTGGATATGGCCAAGGTGGTGCAGGGCAGGGTGGGGCTGCAGCTGCTGCCGCAGCCGCAGCCGCAGGCGGCGCGGGCCAAGGAGGATACGGCGGATATGGTCAGCAAGGCGGAGCTGGAGCGGCTGCTGCAGCGGCTTCAGGACCGGGCCAGATCTACTACGGTCCGCAGTCCGTTGCCGCCCCAGCTGCTGCTGCGGCGTCTGCGTTGGCAGCACCGGCCACTTCTGCACGCATTTCGTCGCACGCGTCCGCATTGCTTAGTAATGGGCCGACAAATCCTGCGTCCATCTCCAACGTAATCAGTAACGCAGTAAGCCAAATTTCTAGCTCTAACCCAGGCGCTTCTGCCTGTGATGTGCTAGTTCAGGCCCTACTTGAGCTCGTAACGGCGCTATTGACTATTATCGGCTCATCAAATATTGGTAGTGTGAACTATGACAGTTCGGGCCAGTACGCACAAGTCGTTACTCAGTCAGTTCAAAACGCCTTCGCC

Amino Acid Sequence:

GGAGQGGQGGYGRGGYGQGGAGQGGAGAAAAAAAAGGAGQGGQGGYGQGGYGQGGAGQGGAAAAAAAAAGGAGQGGYGRGGAGQGGAAAAAAAAAGSGQGGYGGQGAGQGGAGAAAAAAAAGGAGQGGQGGYGRGGYGQGGAGQGGAGAAAAAAAAGGAGQGGQGGYGQGGYGQGGYGQGGAGQGGAAAAAAAAAAGGAGQGGYGRGGAGQGGAAAAAGAGQGGYGGQGAGQGGAGAAAAAAAAGGAGQGGQGGYGRGGYGQGGAGQGGAGAAAAAAAAGGAGQGGQGGYGQGGNGQGGAGQGGAAAAAAAAGGAGQGGYGRGGAGQGGAAAAAAAAAGAGQGGYGGQGAGQGGAGAAAAAAAAGGAGQGGQGGYGRGGYGQGGAGQGGAGAAAAAAAAGGASQGGQGGYGQGDYGQGGAGQGGAAAAAAAAGGAGQGGYGRGGAGQGGAAAAAGAGQGGYGGQGAGQGGAGAAAAAAAAGGAGRGGQGGYGRGGYGQGGAGQGGAGAAAAAAAAGGAGQGGQGGYGQGGYGQGGAGQGGAAAAAAAAAGGAGQGGYGRGGAGQGGAAAAAGAGQGGYGGQGAGQGGAGAAAAAAAAGGAGRGGQGGYGRGGYGQGGAGQGGAGAAAAAAAAGGAGQGGQGGYGQGGYGQGGAGQGGAAAAAAAAVGGAGQGGYGRGGAGQGGAAAAAAAAAAGSGQGGYGGQGAGQGGAGAAAAAAAAGGAGQGGQGGYGGGGYGQGGAGQGGAGAAAAAAAAGGAGQGGQGGYGQGGYGQGGAGQGGAAAAAAAAAGGAGQGGYGRGGAGQGGAAAATGAGQGGYGGQGAGQGGAGAAAAAAAAGGAGQGGQGGYGRGGYGQGGAGQGGAGAAAAAAAAGGAGQGGQGGYGQGGYGQGGAGQGGAAAAAAAAGGAGQGGYGRGGAGQGGAAAAAAAAAGAGQGGYGGQGAGQGGAGAAAAAAAAGGAGQGGQGGYGRGGYGQGGAGQGGAGAAAAGGAGQGGQGGYGQGGYGQGGAGQGGAAAAAAAAAAGGAGQGGYGGYGQQGGAGAAAAAASGPGQIYYGPQSVAAPAAAAASALAAPATSARISSHASALLSNGPTNPASISNVISNAVSQISSSNPGASACDVLVQALLELVTALLTIIGSSNIGSVNYDSSGQYAQVVTQSVQNAFA

**40B LHF1:**

Synthetic DNA Sequence:

GGCGGCGCCGGGCAGGGTGGACAGGGTGGCTACGGAAGAGGAGGCTACGGTCAAGGAGGCGCAGGCCAAGGCGGGGCAGGAGCTGCTGCTGCCGCGGCGGCCGCGGGTGGTGCAGGGCAAGGAGGACAGGGAGGGTACGGCCAAGGAGGATATGGGCAAGGCGGTTACGGTCAGGGTGGAGCTGGACAAGGCGGAGCGGCTGCCGCTGCTGCAGCTGCTGGCGGCGCGGGACAAGGTGGTTACGGGAGGGGAGGTGCCGGACAGGGTGGCGCTGCAGCCGCCGCTGGTGCTGGCCAAGGAGGTTACGGTGGCCAAGGTGCTGGGCAGGGTGGGGCTGGTGCGGCTGCTGCGGCGGCAGCTGCGGGAGGAGCAGGGCAGGGCGGACAAGGTGGCTATGGCAGGGGCGGTTATGGACAGGGCGGCGCAGGACAAGGAGGAGCGGGCGCGGCGGCTGCAGCAGCCGCTGCCGGAGGCGCCGGTCAGGGTGGCCAGGGCGGGTACGGCCAAGGCGGATACGGTCAAGGCGGGGCAGGACAGGGTGGCGCTGCGGCTGCGGCAGCAGCAGCGGCAGGAGGGGCGGGCCAAGGCGGATATGGGCGGGGCGGAGCGGGACAAGGCGGAGCGGCAGCTGCAGCGGCCGCAGCGGCGGGTTCAGGCCAGGGAGGTTACGGCGGCCAAGGTGCCGGACAGGGAGGAGCAGGCGCCGCGGCCGCCGCAGCAGCTGCGGGAGGCGCCGGGCAGGGAGGGCAAGGAGGTTACGGGCGTGGCGGCTACGGACAAGGTGGCGCGGGCCAGGGTGGGGCTGGCGCAGCGGCGGCGGCTGCCGCCGCAGGCGGCGCTGGGCAAGGCGGGCAGGGTGGATACGGGCAGGGCGGATATGGCCAGGGTGGGTACGGTCAAGGAGGTGCCGGACAGGGAGGAGCAGCGGCAGCAGCTGCGGCGGCGGCAGCTGGAGGGGCAGGGCAGGGCGGTTATGGTCGTGGAGGGGCCGGACAAGGAGGTGCAGCTGCAGCAGCGGGAGCTGGGCAGGGTGGATACGGAGGTCAGGGCGCAGGACAGGGTGGTGCTGGCGCTGCGGCTGCGGCAGCCGCGGCGGGAGGCGCGGGGCAGGGAGGCCAAGGCGGGTATGGAAGAGGAGGCTATGGGCAAGGCGGTGCGGGACAAGGTGGGGCCGGTGCTGCGGCGGCTGCAGCGGCTGCTGGAGGTGCTGGCCAAGGAGGGCAAGGCGGATACGGTCAGGGTGGAAATGGGCAAGGCGGAGCGGGCCAGGGCGGCGCGGCCGCTGCCGCGGCCGCAGCAGGCGGCGCGGGCCAAGGCGGGTACGGTCGTGGCGGGGCCGGACAAGGCGGAGCTGCCGCCGCCGCTGCGGCCGCGGCTGGGGCTGGGCAGGGTGGATATGGAGGGCAAGGCGCGGGACAGGGTGGTGCAGGCGCGGCTGCCGCTGCTGCTGCGGCGGGCGGAGCAGGGCAGGGCGGCCAGGGTGGATACGGGAGAGGTGGCTACGGTCAAGGAGGGGCCGGCCAAGGTGGTGCGGGAGCAGCCGCTGCAGCTGCGGCCGCTGGTGGGGCTTCGCAGGGTGGCCAGGGTGGTTACGGGCAAGGTGATTACGGGCAGGGAGGAGCGGGACAAGGTGGGGCCGCCGCTGCGGCTGCGGCAGCAGGTGGCGCTGGACAAGGAGGGTATGGACGAGGAGGGGCAGGCCAAGGCGGGGCTGCCGCTGCTGCAGGGGCAGGTCAAGGCGGGTACGGCGGACAAGGTGCAGGGCAAGGAGGTGCTGGAGCTGCGGCTGCAGCAGCAGCTGCGGGTGGGGCAGGTAGAGGAGGCCAAGGAGGCTACGGAAGAGGCGGATACGGTCAGGGCGGTGCGGGCCAGGGTGGTGCTGGTGCTGCCGCAGCCGCCGCTGCTGCCGGTGGGGCTGGACAGGGTGGACAAGGTGGGTACGGACAAGGTGGCTACGGGCAGGGAGGCGCGGGTCAAGGAGGGGCGGCGGCAGCCGCTGCAGCTGCCGCTGGTGGCGCCGGACAAGGAGGCTATGGCCGTGGTGGGGCGGGGCAAGGCGGCGCGGCGGCCGCGGCGGGTGCGGGGCAGGGCGGCTATGGAGGTCAAGGAGCCGGCCAAGGTGGGGCAGGCGCAGCAGCCGCTGCGGCCGCTGCCGGAGGTGCTGGGCGAGGAGGGCAGGGTGGGTATGGTAGAGGCGGGTACGGGCAGGGAGGCGCCGGACAGGGAGGGGCTGGTGCCGCTGCGGCTGCGGCAGCCGCCGGAGGAGCGGGTCAGGGTGGCCAGGGCGGCTACGGTCAGGGCGGGTATGGGCAGGGCGGGGCTGGACAAGGTGGCGCCGCGGCAGCTGCAGCAGCTGCGGTGGGAGGGGCGGGTCAGGGTGGATACGGCCGAGGTGGGGCAGGTCAAGGCGGTGCTGCGGCCGCGGCGGCCGCTGCAGCCGCTGGTTCTGGTCAGGGAGGTTATGGAGGCCAAGGAGCAGGCCAAGGCGGCGCCGGGGCCGCTGCCGCCGCCGCTGCTGCTGGCGGAGCAGGGCAGGGTGGACAGGGCGGTTACGGTGGTGGAGGATACGGACAAGGAGGTGCCGGCCAAGGCGGAGCTGGAGCTGCTGCGGCAGCAGCAGCCGCGGGAGGCGCTGGACAGGGTGGGCAGGGCGGCTACGGTCAGGGTGGATACGGTCAGGGCGGGGCGGGGCAGGGTGGAGCCGCAGCGGCTGCAGCTGCGGCCGCGGGCGGAGCAGGGCAGGGAGGCTATGGTCGGGGTGGAGCCGGACAAGGAGGCGCGGCGGCCGCTACCGGTGCTGGACAAGGTGGCTATGGCGGTCAAGGAGCAGGACAAGGCGGAGCGGGCGCCGCTGCTGCAGCGGCAGCTGCTGGTGGGGCCGGTCAGGGTGGCCAAGGAGGATACGGCAGGGGTGGCTACGGCCAAGGCGGTGCAGGTCAAGGAGGTGCTGGGGCCGCTGCCGCCGCCGCAGCCGCCGGAGGAGCTGGGCAAGGCGGACAAGGAGGGTATGGCCAAGGTGGGTACGGGCAGGGGGGTGCCGGACAAGGCGGAGCGGCCGCAGCGGCTGCAGCCGCCGGTGGTGCTGGCCAGGGTGGCTATGGGAGAGGCGGGGCCGGTCAAGGAGGCGCCGCGGCAGCTGCGGCGGCCGCGGCAGGGGCGGGGCAGGGCGGTTATGGAGGACAAGGCGCGGGTCAGGGTGGAGCGGGTGCGGCGGCGGCAGCGGCAGCTGCGGGCGGCGCTGGGCAAGGAGGTCAAGGCGGGTACGGTCGAGGTGGCTACGGACAGGGAGGGGCTGGACAAGGTGGGGCGGGTGCCGCAGCGGCGGGAGGAGCCGGTCAGGGTGGGCAAGGCGGCTACGGTCAGGGTGGATATGGCCAAGGTGGTGCAGGGCAGGGTGGGGCTGCAGCTGCTGCCGCAGCCGCAGCCGCAGGCGGCGCGGGCCAAGGAGGATACGGCGGATATGGTCAGCAAGGCGGAGCTGGAGCGGCTGCTGCAGCGGCTTCAGGACCGGGCCAGATCTACTACGGTCCGCAGTCCGTTGCCGCCCCAGCTGCTGCTGCGGCGTCTGCGTTGGCAGCACCGGCCACTTCTGCACGCATTTCGTCGCACGCGTCCGCATTGCTTAGTAATGGGCCGACAAATCCTGCGTCCATCTCCAACGTAATCAGTAACGCAGTAAGCCAAATTTCTAGCTCTAACCCAGGCGCTTCTGCCTGTGATGTGCTAGTTCAGGCCCTACTTGAGCTCGTAACGGCGCTATTGACTATTATCGGCTCATCAAATATTGGTAGTGTGAACTATGACAGTTCGGGCCAGTACGCACAAGTCGTTACTCAGTCAGTTCAAAACGCCTTCGCC

Amino Acid Sequence:

GGAGQGGQGGYGRGGYGQGGAGQGGAGAAAAAAAAGGAGQGGQGGYGQGGYGQGGYGQGGAGQGGAAAAAAAAGGAGQGGYGRGGAGQGGAAAAAGAGQGGYGGQGAGQGGAGAAAAAAAAGGAGQGGQGGYGRGGYGQGGAGQGGAGAAAAAAAAGGAGQGGQGGYGQGGYGQGGAGQGGAAAAAAAAAGGAGQGGYGRGGAGQGGAAAAAAAAAGSGQGGYGGQGAGQGGAGAAAAAAAAGGAGQGGQGGYGRGGYGQGGAGQGGAGAAAAAAAAGGAGQGGQGGYGQGGYGQGGYGQGGAGQGGAAAAAAAAAAGGAGQGGYGRGGAGQGGAAAAAGAGQGGYGGQGAGQGGAGAAAAAAAAGGAGQGGQGGYGRGGYGQGGAGQGGAGAAAAAAAAGGAGQGGQGGYGQGGNGQGGAGQGGAAAAAAAAGGAGQGGYGRGGAGQGGAAAAAAAAAGAGQGGYGGQGAGQGGAGAAAAAAAAGGAGQGGQGGYGRGGYGQGGAGQGGAGAAAAAAAAGGASQGGQGGYGQGDYGQGGAGQGGAAAAAAAAGGAGQGGYGRGGAGQGGAAAAAGAGQGGYGGQGAGQGGAGAAAAAAAAGGAGRGGQGGYGRGGYGQGGAGQGGAGAAAAAAAAGGAGQGGQGGYGQGGYGQGGAGQGGAAAAAAAAAGGAGQGGYGRGGAGQGGAAAAAGAGQGGYGGQGAGQGGAGAAAAAAAAGGAGRGGQGGYGRGGYGQGGAGQGGAGAAAAAAAAGGAGQGGQGGYGQGGYGQGGAGQGGAAAAAAAAVGGAGQGGYGRGGAGQGGAAAAAAAAAAGSGQGGYGGQGAGQGGAGAAAAAAAAGGAGQGGQGGYGGGGYGQGGAGQGGAGAAAAAAAAGGAGQGGQGGYGQGGYGQGGAGQGGAAAAAAAAAGGAGQGGYGRGGAGQGGAAAATGAGQGGYGGQGAGQGGAGAAAAAAAAGGAGQGGQGGYGRGGYGQGGAGQGGAGAAAAAAAAGGAGQGGQGGYGQGGYGQGGAGQGGAAAAAAAAGGAGQGGYGRGGAGQGGAAAAAAAAAGAGQGGYGGQGAGQGGAGAAAAAAAAGGAGQGGQGGYGRGGYGQGGAGQGGAGAAAAGGAGQGGQGGYGQGGYGQGGAGQGGAAAAAAAAAAGGAGQGGYGGYGQQGGAGAAAAAASGPGQIYYGPQSVAAPAAAAASALAAPATSARISSHASALLSNGPTNPASISNVISNAVSQISSSNPGASACDVLVQALLELVTALLTIIGSSNIGSVNYDSSGQYAQVVTQSVQNAFA

***Nephila clavipes* Flagelliform Silk (NCF1), Genbank Ascension AF027973**

Synthetic DNA Sequence:

GGCCCTGGTGGGGTTGGCCCAGGAGGCAGCGGACCAGGAGGGTATGGGCCAGGTGGTGCCGGTCCAGGCGGCTATGGTCCAGGAGGTTCGGGACCTGGAGGGTACGGTCCTGGAGGTAGCGGGCCGGGAGGATATGGACCTGGAGGCTCTGGACCCGGTGGGTACGGACCGGGAGGTTCAGGCCCCGGTGGCTATGGGCCGGGTGGGAGCGGTCCCGGAGGTTATGGTCCGGGTGGATACGGTCCGGGAGGGTCAGGCCCAGGTGGATATGGGCCCGGCGGCACGGGCCCCGGAGGGTCGGGACCAGGTGGGTATGGCCCCGGCGGATCGGGTCCTGGCGGCTCGGGACCCGGCGGGTACGGCCCGGGCGGAAGTGGCCCTGGAGGGTTCGGCCCTGGTGGCTCGGGCCCTGGCGGATACGGTCCAGGCGGCTCAGGACCAGGAGGAGCCGGTCCTGGTGGAGTGGGACCTGGAGGCTTTGGACCGGGTGGCGCAGGACCTGGTGGTGCTGGCCCCGGTGGCGCTGGGCCTGGCGGGGCGGGTCCTGGTGGGGCAGGCCCCGGCGGGGCCGGACCGGGCGGAGCAGGTCCGGGTGGGGCTGGTCCAGGTGGTGCGGGACCAGGCGGAGCGGGACCCGGAGGGGCCGGCGGCGCAGGCGGTGCGGGAGGCGCTGGAGGATCGGGTGGTGCAGGAGGGTCTGGAGGGACTACTATTATTGAAGATCTGGACATTACTATTGATGGGGCGGATGGCCCAATCACTATCTCAGAAGAGCTTACCATCTCCGGCGCGGGTGGCTCGGGGCCAGGAGGTGCAGGTCCGGGAGGGGTCGGTCCAGGAGGTAGTGGACCTGGCGGGGTAGGGCCGGGAGGTAGCGGCCCAGGAGGAGTGGGTCCTGGAGGTTCTGGTCCCGGCGGAGTCGGTCCCGGAGGGGCCGGTGGGCCCTATGGGCCCGGAGGTTCGGGGCCCGGCGGGGCGGGAGGTGCCGGCGGTCCGGGTGGAGCTTATGGGCCAGGCGGTAGCTACGGGCCAGGTGGTTCGGGCGGCCCTGGAGGCGCCGGTGGTCCATACGGGCCTGGCGGCGAAGGCCCCGGTGGCGCAGGCGGTCCATATGGCCCTGGTGGCGCTGGTGGTCCCTACGGACCCGGTGGTGCAGGTGGGCCATACGGCCCGGGTGGAGAAGGAGGTCCTTACGGTCCAGGAGGTTCCTATGGTCCAGGTGGAGCCGGCGGGCCTTATGGCCCCGGAGGCCCCTATGGCCCGGGCGGGGAAGGTCCTGGCGGGGCTGGTGGACCATATGGACCCGGCGGTGTAGGCCCTGGAGGTTCCGGACCAGGTGGTTATGGGCCTGGAGGGTCGGGTCCTGGAGGATACGGACCGGGCGGTGCGGGCCCAGGTGGGTATGGTCCGGGTGGGAGCGGTCCCGGCGGGTACGGACCTGGTGGCTCGGGGCCTGGCGGTTACGGCCCTGGCGGAAGCGGTCCGGGAGGGTACGGGCCCGGCGGATCAGGTCCAGGCGGCTATGGCAGTGGCGGAGCGGGTCCGGGCGGATATGGGCCAGGAGGATCGGGACCTGGAGGGTACGGTCCTGGTGGCTCAGGCCCTGGCGGCTATGGGCCAGGAGGCACGGGGCCTGGAGGGACCGGCCCGGGTGGTAGTGGACCTGGAGGGTATGGTCCAGGAGGAAGTGGCCCAGGAGGCTCGGGCCCCGGCGGCTCGGGACCTGGCGGATACGGACCCTCAGGCTCTGGTCCTGGAGGCTACGGTCCGAGTGGATCGGGCCCTGGAGGTTACGGGCCTGGTGGATCAGGACCTGGTGGTTACGGACCGGGAGGAAGCGGTGCGGGAGGGACGGGGCCGGGTGGAGCAGGAGGCGCTGGTGGTGCCGGCGGCAGCGGAGGGGCAGGTGGTTCTGGTGGCGCAGGCGGTTCGGGTGGCGCCGGAGGATCAGGCGGTGTTGGTGGGTCCGGTGGAACGACAATCACGGAGGACTTGGATATCACGATCGATGGTGCCGACGGTCCCATTACCATCAGTGAGGAACTGACAATCAGTGGTGCTGGAGGGTCTGGGCCGGGCGGCGCAGGACCCGGCGGCGTCGGACCTGGAGGGAGTGGACCGGGCGGAGTAGGCCCGGGCGTCAGTGGACCTGGCGGTGTAGGGCCTGGTGGGTCGGGGCCGGGAGGGGTCGGTTCTGGCGGAAGCGGGCCAGGAGGCGTGGGACCGGGAGGCTATGGGCCAGGTGGCTCCGGTAGCGGTGGCGTTGGACCCGGAGGTTACGGTCCGGGCGGGTCGGGTGGGTTTTACGGGCCCGGCGGTTCTGAAGGGCCATACGGACCCAGCGGAACGTACGGCTCTGGAGGCGGCTATGGTCCCGGTGGGGCAGGAGGTCCCTACGGTCCCGGGAGCCCTGGCGGGGCATACGGTCCTGGTTCTCCCGGCGGTGCTTATTATCCCAGTTCTCGGGTGCCGGACATGGTGAATGGTATCATGTCCGCTATGCAAGGTAGTGGTTTTAACTACCAGATGTTCGGCAACATGCTATCGCAGTATAGCTCCGGGAGCGGCACATGCAATCCTAATAATGTCAATGTGCTGATGGACGCTCTACTCGCCGCCTTACATTGCCTATCAAATCATGGCTCATCCTCATTTGCGCCTAGCCCCACTCCCGCTGCGATGTCGGCCTACAGCAATAGCGTAGGACGGATGTTCGCCTAT

Amino Acid Sequence:

GPGGVGPGGSGPGGYGPGGAGPGGYGPGGSGPGGYGPGGSGPGGYGPGGSGPGGYGPGGSGPGGYGPGGSGPGGYGPGGYGPGGSGPGGYGPGGTGPGGSGPGGYGPGGSGPGGSGPGGYGPGGSGPGGFGPGGSGPGGYGPGGSGPGGAGPGGVGPGGFGPGGAGPGGAGPGGAGPGGAGPGGAGPGGAGPGGAGPGGAGPGGAGPGGAGPGGAGGAGGAGGAGGSGGAGGSGGTTIIEDLDITIDGADGPITISEELTISGAGGSGPGGAGPGGVGPGGSGPGGVGPGGSGPGGVGPGGSGPGGVGPGGAGGPYGPGGSGPGGAGGAGGPGGAYGPGGSYGPGGSGGPGGAGGPYGPGGEGPGGAGGPYGPGGAGGPYGPGGAGGPYGPGGEGGPYGPGGSYGPGGAGGPYGPGGPYGPGGEGPGGAGGPYGPGGVGPGGSGPGGYGPGGSGPGGYGPGGAGPGGYGPGGSGPGGYGPGGSGPGGYGPGGSGPGGYGPGGSGPGGYGSGGAGPGGYGPGGSGPGGYGPGGSGPGGYGPGGTGPGGTGPGGSGPGGYGPGGSGPGGSGPGGSGPGGYGPSGSGPGGYGPSGSGPGGYGPGGSGPGGYGPGGSGAGGTGPGGAGGAGGAGGSGGAGGSGGAGGSGGAGGSGGVGGSGGTTITEDLDITIDGADGPITISEELTISGAGGSGPGGAGPGGVGPGGSGPGGVGPGVSGPGGVGPGGSGPGGVGSGGSGPGGVGPGGYGPGGSGSGGVGPGGYGPGGSGGFYGPGGSEGPYGPSGTYGSGGGYGPGGAGGPYGPGSPGGAYGPGSPGGAYYPSSRVPDMVNGIMSAMQGSGFNYQMFGNMLSQYSSGSGTCNPNNVNVLMDALLAALHCLSNHGSSSFAPSPTPAAMSAYSNSVGRMFAY

**1B NCF1:**

Synthetic DNA Sequence:

TCCGGTGGAACGACAATCACGGAGGACTTGGATATCACGATCGATGGTGCCGACGGTCCCATTACCATCAGTGAGGAACTGACAATCAGTGGTGCTGGAGGGTCTGGGCCGGGCGGCGCAGGACCCGGCGGCGTCGGACCTGGAGGGAGTGGACCGGGCGGAGTAGGCCCGGGCGTCAGTGGACCTGGCGGTGTAGGGCCTGGTGGGTCGGGGCCGGGAGGGGTCGGTTCTGGCGGAAGCGGGCCAGGAGGCGTGGGACCGGGAGGCTATGGGCCAGGTGGCTCCGGTAGCGGTGGCGTTGGACCCGGAGGTTACGGTCCGGGCGGGTCGGGTGGGTTTTACGGGCCCGGCGGTTCTGAAGGGCCATACGGACCCAGCGGAACGTACGGCTCTGGAGGCGGCTATGGTCCCGGTGGGGCAGGAGGTCCCTACGGTCCCGGGAGCCCTGGCGGGGCATACGGTCCTGGTTCTCCCGGCGGTGCTTATTATCCCAGTTCTCGGGTGCCGGACATGGTGAATGGTATCATGTCCGCTATGCAAGGTAGTGGTTTTAACTACCAGATGTTCGGCAACATGCTATCGCAGTATAGCTCCGGGAGCGGCACATGCAATCCTAATAATGTCAATGTGCTGATGGACGCTCTACTCGCCGCCTTACATTGCCTATCAAATCATGGCTCATCCTCATTTGCGCCTAGCCCCACTCCCGCTGCGATGTCGGCCTACAGCAATAGCGTAGGACGGATGTTCGCCTAT

Amino Acid Sequence:

SGGTTITEDLDITIDGADGPITISEELTISGAGGSGPGGAGPGGVGPGGSGPGGVGPGVSGPGGVGPGGSGPGGVGSGGSGPGGVGPGGYGPGGSGSGGVGPGGYGPGGSGGFYGPGGSEGPYGPSGTYGSGGGYGPGGAGGPYGPGSPGGAYGPGSPGGAYYPSSRVPDMVNGIMSAMQGSGFNYQMFGNMLSQYSSGSGTCNPNNVNVLMDALLAALHCLSNHGSSSFAPSPTPAAMSAYSNSVGRMFAY

**2B NCF1:**

Synthetic DNA Sequence:

ACTACTATTATTGAAGATCTGGACATTACTATTGATGGGGCGGATGGCCCAATCACTATCTCAGAAGAGCTTACCATCTCCGGCGCGGGTGGCTCGGGGCCAGGAGGTGCAGGTCCGGGAGGGGTCGGTCCAGGAGGTAGTGGACCTGGCGGGGTAGGGCCGGGAGGTAGCGGCCCAGGAGGAGTGGGTCCTGGAGGTTCTGGTCCCGGCGGAGTCGGTCCCGGAGGGGCCGGTGGGCCCTATGGGCCCGGAGGTTCGGGGCCCGGCGGGGCGGGAGGTGCCGGCGGTCCGGGTGGAGCTTATGGGCCAGGCGGTAGCTACGGGCCAGGTGGTTCGGGCGGCCCTGGAGGCGCCGGTGGTCCATACGGGCCTGGCGGCGAAGGCCCCGGTGGCGCAGGCGGTCCATATGGCCCTGGTGGCGCTGGTGGTCCCTACGGACCCGGTGGTGCAGGTGGGCCATACGGCCCGGGTGGAGAAGGAGGTCCTTACGGTCCAGGAGGTTCCTATGGTCCAGGTGGAGCCGGCGGGCCTTATGGCCCCGGAGGCCCCTATGGCCCGGGCGGGGAAGGTCCTGGCGGGGCTGGTGGACCATATGGACCCGGCGGTGTAGGCCCTGGAGGTTCCGGACCAGGTGGTTATGGGCCTGGAGGGTCGGGTCCTGGAGGATACGGACCGGGCGGTGCGGGCCCAGGTGGGTATGGTCCGGGTGGGAGCGGTCCCGGCGGGTACGGACCTGGTGGCTCGGGGCCTGGCGGTTACGGCCCTGGCGGAAGCGGTCCGGGAGGGTACGGGCCCGGCGGATCAGGTCCAGGCGGCTATGGCAGTGGCGGAGCGGGTCCGGGCGGATATGGGCCAGGAGGATCGGGACCTGGAGGGTACGGTCCTGGTGGCTCAGGCCCTGGCGGCTATGGGCCAGGAGGCACGGGGCCTGGAGGGACCGGCCCGGGTGGTAGTGGACCTGGAGGGTATGGTCCAGGAGGAAGTGGCCCAGGAGGCTCGGGCCCCGGCGGCTCGGGACCTGGCGGATACGGACCCTCAGGCTCTGGTCCTGGAGGCTACGGTCCGAGTGGATCGGGCCCTGGAGGTTACGGGCCTGGTGGATCAGGACCTGGTGGTTACGGACCGGGAGGAAGCGGTGCGGGAGGGACGGGGCCGGGTGGAGCAGGAGGCGCTGGTGGTGCCGGCGGCAGCGGAGGGGCAGGTGGTTCTGGTGGCGCAGGCGGTTCGGGTGGCGCCGGAGGATCAGGCGGTGTTGGTGGGTCCGGTGGAACGACAATCACGGAGGACTTGGATATCACGATCGATGGTGCCGACGGTCCCATTACCATCAGTGAGGAACTGACAATCAGTGGTGCTGGAGGGTCTGGGCCGGGCGGCGCAGGACCCGGCGGCGTCGGACCTGGAGGGAGTGGACCGGGCGGAGTAGGCCCGGGCGTCAGTGGACCTGGCGGTGTAGGGCCTGGTGGGTCGGGGCCGGGAGGGGTCGGTTCTGGCGGAAGCGGGCCAGGAGGCGTGGGACCGGGAGGCTATGGGCCAGGTGGCTCCGGTAGCGGTGGCGTTGGACCCGGAGGTTACGGTCCGGGCGGGTCGGGTGGGTTTTACGGGCCCGGCGGTTCTGAAGGGCCATACGGACCCAGCGGAACGTACGGCTCTGGAGGCGGCTATGGTCCCGGTGGGGCAGGAGGTCCCTACGGTCCCGGGAGCCCTGGCGGGGCATACGGTCCTGGTTCTCCCGGCGGTGCTTATTATCCCAGTTCTCGGGTGCCGGACATGGTGAATGGTATCATGTCCGCTATGCAAGGTAGTGGTTTTAACTACCAGATGTTCGGCAACATGCTATCGCAGTATAGCTCCGGGAGCGGCACATGCAATCCTAATAATGTCAATGTGCTGATGGACGCTCTACTCGCCGCCTTACATTGCCTATCAAATCATGGCTCATCCTCATTTGCGCCTAGCCCCACTCCCGCTGCGATGTCGGCCTACAGCAATAGCGTAGGACGGATGTTCGCCTAT

Amino Acid Sequence:

TTIIEDLDITIDGADGPITISEELTISGAGGSGPGGAGPGGVGPGGSGPGGVGPGGSGPGGVGPGGSGPGGVGPGGAGGPYGPGGSGPGGAGGAGGPGGAYGPGGSYGPGGSGGPGGAGGPYGPGGEGPGGAGGPYGPGGAGGPYGPGGAGGPYGPGGEGGPYGPGGSYGPGGAGGPYGPGGPYGPGGEGPGGAGGPYGPGGVGPGGSGPGGYGPGGSGPGGYGPGGAGPGGYGPGGSGPGGYGPGGSGPGGYGPGGSGPGGYGPGGSGPGGYGSGGAGPGGYGPGGSGPGGYGPGGSGPGGYGPGGTGPGGTGPGGSGPGGYGPGGSGPGGSGPGGSGPGGYGPSGSGPGGYGPSGSGPGGYGPGGSGPGGYGPGGSGAGGTGPGGAGGAGGAGGSGGAGGSGGAGGSGGAGGSGGVGGSGGTTITEDLDITIDGADGPITISEELTISGAGGSGPGGAGPGGVGPGGSGPGGVGPGVSGPGGVGPGGSGPGGVGSGGSGPGGVGPGGYGPGGSGSGGVGPGGYGPGGSGGFYGPGGSEGPYGPSGTYGSGGGYGPGGAGGPYGPGSPGGAYGPGSPGGAYYPSSRVPDMVNGIMSAMQGSGFNYQMFGNMLSQYSSGSGTCNPNNVNVLMDALLAALHCLSNHGSSSFAPSPTPAAMSAYSNSVGRMFAY

***Bombyx mori* Heavy Chain Fibroin 3’ partial (BMF1), Genbank Ascension S74439**

Synthetic DNA Sequence:

GCGGGAACGGGGTCATCAGGTTTTGGCCCTTATGTAGCGAACGGTGGCTATTCTGGCTATGAGTACGCTTGGTCTAGTGAAAGTGATTTTGGTACCGGTAGTGGGGCAGGCGCAGGCAGCGGCGCTGGGGCCGGTAGTGGGGCGGGAGCTGGATATGGTGCCGGTGTTGGCGCAGGCTATGGGGCCGGATATGGTGCAGGTGCGGGAGCAGGGTACGGGGCAGGCGCTGGAAGTGGGGTCGCTTCGGGAGCCGGTGCCGGCGCGGGGTCTGGAGCCGGCGCGGGAAGCGGGGCGGGTGCCGGGTCAGGAGCGGGTGCTGGTTCAGGCGCAGGAGCTGGCTCCGGGGCGGGTGCAGGGTCCGGCGCAGGGGCTGGTTATGGCGCTGGCGCTGGTTATGGCGCTGGCGCAGGTTATGGGGCGGGTGCTGGAGTCGGGTATGGTGCTGGCGCAGGTGTAGGATACGGTGCGGGTGCGGGATATGGGGCGGGAGCCGGTGTGGGGTATGGTGCCGGGGCAGGGAGTGGCGCGGCCTCAGGCGCCGGGGCCGGCTCAGGAGCAGGCGCGGGAAGCGGGGCTGGCGCAGGTTCGGGTGCAGGAGCAGGCTCGGGAGCTGGTGCAGGATCGGGAGCTGGGGCAGGCAGTGGGGCCGGAGCAGGTTCCGGTGCTGGCGCAGGTAGCGGAGCCGGTGCGGGTTATGGCGCGGGAGCTGGAGTAGGCTATGGTGCAGGTGCGGGTTCAGGAGCTGCTAGTGGCGCGGGCGCTGGTTCCGGAGCAGGTGCTGGCTCGGGCGCCGGAGCCGGGAGTGGCGCAGGCGCTGGATCAGGTGCCGGAGCAGGATCTGGGGCTGGTGCCGGATCGGGAGCCGGTGCCGGGTCAGGGGCCGGCGCTGGGTCGGGCGCTGGAAGTGGTGCAGGGGCAGGTTCAGGCGCAGGAGCGGGTTATGGTGCGGGTGCCGGAGCAGGCGTCGGGTACGGAGCTGGGGCCGGTGCAGGTTATGGAGCGGGCTACGGTTATGGCGCTGGTGCCGGGGTGGGTTACGGTGCGGGCGCAGGTTCTGGCGCCGCCAGTGGGGCCGGCGCAGGCTCAGGTGCAGGAGCCGGTAGTGGGGCGGGCGCCGGGAGTGGGGCCGGCGCAGGCAGCGGAGCCGGGGCTGGTTCAGGAGCGGGGTCAGGCGCGGGAGCTGGAAGTGGGGCCGGGGCGGGTTACGGTGCCGGATACGGAGCCGGTGTTGGGGCAGGGTACGGAGCCGGCGCAGGCGTAGGGTATGGGGCAGGATATGGTGTAGGAGCTGGAGCAGGTTACGGCGCAGGTGCTGGGTCTGGCGCCGCATCAGGAGCCGGGGCCGGCAGTGGTGCGGGCGCTGGGTCTGGGGCCGGTGCCGGTTCCGGGGCGGGAGCAGGCAGCGGTGCAGGGGCGGGATCGGGCGCTGGCTCAGGCGCCGGAGCTGGGTATGGGGCGGGTGCTGGAAGTGGAGCGGCGTCAGGAGCCGGAGCAGGTGCCGGGGCTGGTACAGGTAGCAGCGGATTCGGACCATACGTTGCCAATGGTGGATATTCCCGACGCGAAGGGTACGAATATGCCTGGTCTAGTAAATCGGATTTTGAGACAGGATCAGGTGCGGCTTCGGGAGCTGGGGCAGGCGCCGGGTCAGGGGCCGGTGCAGGTTCGGGCGCGGGTGCTGGATCTGGGGCTGGCGCAGGCAGTGGTGCGGGGGCGGGAGGTAGCGTTTCTTACGGTGCCGGTAGAGGGTATGGGCAGGGCGCTGGCTCGGCCGCGAGCTCCGTGTCCAGTGCTTCGAGTAGAAGTTACGATTACTCGCGCCGTAATGTCAGGAAGAATTGTGGTATCCCACGACGCCAGCTCGTAGTAAAATTTCGGGCCCTCCCGTGCGTAAACTGC

Amino Acid Sequence:

AGTGSSGFGPYVANGGYSGYEYAWSSESDFGTGSGAGAGSGAGAGSGAGAGYGAGVGAGYGAGYGAGAGAGYGAGAGSGVASGAGAGAGSGAGAGSGAGAGSGAGAGSGAGAGSGAGAGSGAGAGYGAGAGYGAGAGYGAGAGVGYGAGAGVGYGAGAGYGAGAGVGYGAGAGSGAASGAGAGSGAGAGSGAGAGSGAGAGSGAGAGSGAGAGSGAGAGSGAGAGSGAGAGYGAGAGVGYGAGAGSGAASGAGAGSGAGAGSGAGAGSGAGAGSGAGAGSGAGAGSGAGAGSGAGAGSGAGSGAGAGSGAGAGYGAGAGAGVGYGAGAGAGYGAGYGYGAGAGVGYGAGAGSGAASGAGAGSGAGAGSGAGAGSGAGAGSGAGAGSGAGSGAGAGSGAGAGYGAGYGAGVGAGYGAGAGVGYGAGYGVGAGAGYGAGAGSGAASGAGAGSGAGAGSGAGAGSGAGAGSGAGAGSGAGSGAGAGYGAGAGSGAASGAGAGAGAGTGSSGFGPYVANGGYSRREGYEYAWSSKSDFETGSGAASGAGAGAGSGAGAGSGAGAGSGAGAGSGAGAGGSVSYGAGRGYGQGAGSAASSVSSASSRSYDYSRRNVRKNCGIPRRQLVVKFRALPCVNC

***Araneus diadematus* ADF1, Genbank Ascension U47853**

Synthetic DNA Sequence:

CATGAATCTTCCTATGCTGCTGCAATGGCTGCTTCTACTCGTAATTCTGATTTTATCCGTAACATGAGCTACCAGATGGGTCGTCTGCTGAGCAACGCCGGTGCCATTACCGAATCTACTGCAAGCAGCGCGGCTTCCAGCGCGTCCTCCACCGTTACCGAGTCTATTCGCACGTATGGCCCGGCTGCGATCTTTTCTGGTGCGGGCGCTGGCGCAGGCGTGGGTGTAGGTGGTGCCGGTGGTTACGGCCAGGGCTACGGTGCAGGCGCAGGTGCTGGTGCGGGCGCCGGTGCGGGTGCTGGTGGCGCGGGTGGCTACGGTCAGGGCTACGGTGCGGGTGCTGCCGCGGCGGCTGGCGCAGGCGCAGGTGCAGCGGGCGGTTACGGCGGTGGTTCTGGTGCTGGCGCTGGTGGTGCTGGTGGTTATGGCCAGGGTTACGGTGCAGGTTCTGGCGCGGGTGCGGGCGCTGCTGCGGCAGCTGGCGCATCCGCTGGTGCTGCTGGCGGCTATGGCGGTGGCGCAGGTGTTGGTGCAGGTGCGGGCGCGGGTGCGGCTGGTGGCTATGGCCAGAGCTATGGCAGCGGTGCTGGCGCAGGTGCGGGTGCTGGTGCGGCGGCTGCAGCTGGCGCTGGCGCACGTGCAGCGGGTGGCTACGGTGGTGGTTACGGCGCAGGCGCGGGCGCCGGTGCTGGCGCCGCTGCTTCCGCTGGTGCCTCCGGTGGCTACGGTGGCGGTTACGGCGGTGGCGCGGGTGCAGGCGCCGTAGCTGGTGCGTCCGCGGGTTCTTACGGCGGTGCGGTTAACCGTCTGTCTAGCGCAGGCGCGGCATCTCGTGTTTCCAGCAACGTGGCTGCCATCGCGTCTGCGGGTGCGGCTGCGCTGCCGAACGTAATCTCTAACATTTATTCTGGTGTGCTGTCCTCTGGTGTGTCTTCTTCTGAGGCGCTGATCCAGGCTCTGCTGGAAGTCATCTCTGCACTGATCCACGTGCTGGGTTCTGCCTCCATCGGTAACGTGTCTTCCGTTGGCGTTAACAGCGCACTGAATGCAGTGCAGAACGCCGTCGGCGCGTACGCTGGT

Amino Acid Sequence:

HESSYAAAMAASTRNSDFIRNMSYQMGRLLSNAGAITESTASSAASSASSTVTESIRTYGPAAIFSGAGAGAGVGVGGAGGYGQGYGAGAGAGAGAGAGAGGAGGYGQGYGAGAAAAAGAGAGAAGGYGGGSGAGAGGAGGYGQGYGAGSGAGAGAAAAAGASAGAAGGYGGGAGVGAGAGAGAAGGYGQSYGSGAGAGAGAGAAAAAGAGARAAGGYGGGYGAGAGAGAGAAASAGASGGYGGGYGGGAGAGAVAGASAGSYGGAVNRLSSAGAASRVSSNVAAIASAGAAALPNVISNIYSGVLSSGVSSSEALIQALLEVISALIHVLGSASIGNVSSVGVNSALNAVQNAVGAYAG

***Araneus diadematus* ADF2, Genbank Ascension U47854**

Synthetic DNA Sequence:

GGTAGCCAAGGCGCAGGTGGTGCAGGTCAAGGTGGTTATGGTGCAGGCGGCGGTGGCGCTGCGGCAGCTGCTGCTGCAGCGGTAGGCGCGGGTGGCGGTGGTCAGGGCGGCCTGGGTTCCGGCGGTGCGGGCCAGGGTTACGGCGCAGGCCTGGGCGGTCAAGGTGGCGCATCTGCGGCGGCTGCTGCGGCTGGTGGCCAGGGCGGTCAGGGTGGCCAAGGTGGCTATGGCGGTCTGGGTTCTCAGGGCGCAGGCGGTGCTGGTCAGCTGGGCTATGGTGCAGGTCAGGAATCTGCAGCGGCTGCCGCTGCCGCAGCGGGCGGCGCTGGTGGCGGTGGTCAGGGCGGCCTGGGTGCGGGTGGCGCTGGCCAAGGTTACGGTGCCGCTGGCCTGGGCGGTCAGGGTGGTGCGGGCCAGGGCGGCGGCTCTGGCGCGGCGGCTGCGGCCGGTGGTCAAGGTGGTCAGGGCGGCTATGGTGGCCTGGGCCCGCAAGGCGCGGGTGGTGCGGGCCAGGGTGGCTACGGTGGTGGTTCCCTGCAATACGGCGGTCAGGGTCAGGCTCAGGCAGCTGCGGCATCCGCGGCGGCATCCCGCCTGTCTTCCCCATCCGCAGCGGCACGTGTGTCTTCCGCTGTATCTCTGGTATCCAACGGCGGTCCGACCAGCCCGGCGGCACTGAGCTCTAGCATTTCCAACGTGGTATCTCAGATCTCTGCAAGCAACCCAGGCCTGTCTGGTTGCGATATCCTGGTTCAAGCCCTGCTGGAAATTATCTCTGCGCTGGTTCACATCCTGGGTTCTGCCAACATCGGCCCGGTTAACTCTAGCTCCGCCGGTCAGTCCGCATCCATTGTAGGTCAATCCGTATACCGCGCTCTGTCT

Amino Acid Sequence:

GSQGAGGAGQGGYGAGGGGAAAAAAAAVGAGGGGQGGLGSGGAGQGYGAGLGGQGGASAAAAAAGGQGGQGGQGGYGGLGSQGAGGAGQLGYGAGQESAAAAAAAAGGAGGGGQGGLGAGGAGQGYGAAGLGGQGGAGQGGGSGAAAAAGGQGGQGGYGGLGPQGAGGAGQGGYGGGSLQYGGQGQAQAAAASAAASRLSSPSAAARVSSAVSLVSNGGPTSPAALSSSISNVVSQISASNPGLSGCDILVQALLEIISALVHILGSANIGPVNSSSAGQSASIVGQSVYRALS

***Araneus diadematus* ADF3, Genbank Ascension U47855**

Synthetic DNA Sequence:

GCCCGCGCGGGGTCAGGCCAGCAGGGACCAGGTCAACAGGGCCCGGGCCAACAAGGCCCGGGTCAACAGGGTCCGTACGGTCCGGGTGCCAGCGCGGCGGCCGCGGCCGCAGGAGGGTATGGCCCTGGTAGCGGCCAACAGGGTCCGAGCCAGCAAGGCCCGGGCCAGCAAGGGCCGGGGGGCCAGGGGCCCTACGGCCCTGGTGCGTCAGCTGCCGCAGCCGCAGCTGGCGGTTATGGCCCGGGGTCAGGTCAGCAAGGGCCAGGCGGTCAAGGTCCTTACGGGCCAGGCAGTAGTGCGGCAGCGGCTGCTGCCGGTGGTAACGGCCCGGGGTCGGGCCAGCAAGGGGCGGGACAGCAGGGTCCAGGCCAACAAGGCCCCGGTGCGTCCGCAGCGGCGGCGGCCGCTGGTGGCTATGGCCCGGGTTCAGGCCAGCAGGGCCCGGGGCAGCAGGGCCCGGGTGGACAGGGTCCGTATGGCCCGGGGGCCAGTGCAGCGGCCGCGGCTGCTGGGGGCTATGGCCCTGGCTCAGGTCAGGGTCCGGGTCAACAAGGACCCGGCGGTCAAGGACCGTATGGCCCGGGTGCGTCCGCGGCGGCTGCGGCGGCTGGAGGCTATGGTCCGGGAAGTGGCCAACAGGGCCCTGGACAGCAGGGTCCGGGTCAGCAGGGACCCGGTGGACAGGGCCCGTATGGGCCAGGCGCCTCTGCCGCAGCGGCGGCCGCAGGTGGGTATGGACCGGGGTACGGCCAGCAGGGTCCTGGTCAGCAGGGACCGGGCGGCCAGGGCCCTTACGGCCCCGGCGCGTCAGCTGCAAGCGCTGCCTCGGGTGGCTACGGCCCGGGTTCCGGTCAGCAGGGCCCGGGACAGCAGGGTCCGGGTCAGCAGGGACCGTATGGTCCGGGAGCTTCTGCTGCTGCCGCCGCGGCGGGTGGTTATGGACCCGGCAGTGGCCAACAAGGTCCGGGGCAGCAGGGTCCAGGTCAGCAGGGCCCAGGACAGCAGGGCCCTGGTGGCCAAGGACCGTACGGTCCCGGCGCAAGTGCGGCCGCTGCAGCTGCCGGAGGCTACGGTCCAGGTAGTGGACAGCAAGGACCGGGTCAGCAGGGCCCCGGTCAACAGGGGCCGGGCCAGCAAGGCCCCGGGCAGCAGGGACCTGGGCAGCAGGGTCCCGGGCAGCAAGGTCCTGGGCAACAGGGTCCGGGACAGCAAGGCCCTGGCGGCCAGGGTGCGTATGGGCCTGGTGCATCTGCCGCGGCGGGCGCCGCGGGTGGGTACGGGCCGGGGAGCGGCCAGCAAGGTCCGGGCCAACAGGGCCCCGGACAACAGGGTCCTGGCCAGCAAGGACCTGGCCAGCAGGGGCCGGGACAACAAGGGCCCGGCCAACAAGGCCCAGGGCAACAAGGCCCGTACGGCCCTGGGGCCTCGGCAGCCGCGGCAGCGGCCGGCGGCTATGGCCCGGGCAGTGGTCAACAAGGTCCAGGCCAACAGGGCCCAGGGCAGCAGGGTCCGGGGGGTCAAGGTCCGTACGGACCGGGTGCCGCCTCGGCAGCGGTGAGTGTAGGCGGCTACGGACCTCAAAGCTCCTCTGTGCCAGTCGCCAGTGCGGTGGCTAGCCGTCTGTCTAGCCCCGCCGCCAGCAGTCGTGTCAGCTCAGCCGTGTCGTCTTTAGTATCATCAGGACCGACTAAACACGCAGCCTTGTCAAACACCATTAGCAGCGTTGTCTCTCAGGTGTCAGCGAGTAACCCGGGGCTGTCGGGTTGCGACGTCCTGGTACAGGCCCTGCTGGAAGTGGTGAGCGCCCTCGTGTCTATTCTGGGTTCTAGTTCCATTGGCCAGATTAACTATGGGGCGAGTGCGCAATACACCCAAATGGTCGGACAATCTGTTGCGCAGGCACTGGCG

Amino Acid Sequence:

ARAGSGQQGPGQQGPGQQGPGQQGPYGPGASAAAAAAGGYGPGSGQQGPSQQGPGQQGPGGQGPYGPGASAAAAAAGGYGPGSGQQGPGGQGPYGPGSSAAAAAAGGNGPGSGQQGAGQQGPGQQGPGASAAAAAAGGYGPGSGQQGPGQQGPGGQGPYGPGASAAAAAAGGYGPGSGQGPGQQGPGGQGPYGPGASAAAAAAGGYGPGSGQQGPGQQGPGQQGPGGQGPYGPGASAAAAAAGGYGPGYGQQGPGQQGPGGQGPYGPGASAASAASGGYGPGSGQQGPGQQGPGQQGPYGPGASAAAAAAGGYGPGSGQQGPGQQGPGQQGPGQQGPGGQGPYGPGASAAAAAAGGYGPGSGQQGPGQQGPGQQGPGQQGPGQQGPGQQGPGQQGPGQQGPGQQGPGGQGAYGPGASAAAGAAGGYGPGSGQQGPGQQGPGQQGPGQQGPGQQGPGQQGPGQQGPGQQGPYGPGASAAAAAAGGYGPGSGQQGPGQQGPGQQGPGGQGPYGPGAASAAVSVGGYGPQSSSVPVASAVASRLSSPAASSRVSSAVSSLVSSGPTKHAALSNTISSVVSQVSASNPGLSGCDVLVQALLEVVSALVSILGSSSIGQINYGASAQYTQMVGQSVAQALA

***Araneus diadematus* ADF4, Genbank Ascension U47856**

Synthetic DNA Sequence:

GCAGGCTCTAGCGCCGCAGCTGCCGCTGCTGCAAGCGGTAGCGGTGGTTACGGTCCAGAGAACCAGGGTCCGTCCGGCCCAGTAGCATATGGCCCTGGTGGTCCAGTCTCTTCCGCTGCTGCCGCAGCTGCTGCGGGCTCCGGTCCAGGTGGCTACGGTCCGGAAAACCAGGGCCCGTCTGGTCCGGGCGGTTATGGCCCGGGTGGCTCTGGTAGCTCTGCAGCGGCGGCAGCCGCGGCAGCGTCTGGCCCAGGTGGTTACGGCCCAGGCTCCCAGGGCCCGTCCGGTCCGGGCGGTAGCGGCGGTTATGGTCCTGGTTCCCAGGGTGCAAGCGGCCCTGGTGGTCCGGGCGCATCTGCGGCAGCCGCCGCAGCAGCGGCTGCGGCAAGCGGTCCGGGTGGCTACGGTCCGGGCAGCCAGGGTCCGTCTGGTCCTGGCGCCTACGGTCCAGGTGGCCCGGGTTCCTCCGCTGCAGCCGCGGCTGCGGCTGCGAGCGGTCCTGGTGGCTACGGTCCGGGTAGCCAGGGTCCTTCCGGTCCAGGTGTGTACGGCCCTGGTGGCCCGGGTTCTTCTGCTGCTGCAGCGGCGGCTGCTGGCTCTGGTCCGGGCGGTTATGGCCCGGAAAACCAGGGTCCGTCTGGCCCTGGTGGTTACGGCCCAGGTGGTTCCGGTTCCTCCGCTGCTGCGGCGGCAGCAGCTGCCAGCGGTCCAGGCGGTTACGGTCCTGGCTCTCAAGGCCCGTCCGGCCCTGGCGGTTCCGGTGGCTATGGTCCGGGTTCTCAGGGCGGTTCTGGTCCGGGCGCGAGCGCAGCTGCAGCGGCAGCCGCTGCATCTGGTCCTGGCGGTTACGGTCCGGGTAGCCAGGGTCCATCCGGTCCGGGCTATCAGGGCCCGTCTGGCCCGGGTGCTTATGGTCCATCCCCGAGCGCATCTGCGTCCGTGGCCGCTTCCGTCTATCTGCGTCTGCAACCGCGTCTGGAAGTTTCCTCTGCTGTTAGCAGCCTGGTTTCCAGCGGTCCGACTAACGGCGCTGCTGTCTCTGGCGCCCTGAACTCTCTGGTTTCCCAGATTTCTGCAAGCAACCCTGGTCTGTCTGGTTGCGACGCGCTGGTGCAGGCTCTGCTGGAACTGGTTTCTGCGCTGGTTGCAATCCTGAGCAGCGCAAGCATCGGTCAGGTTAACGTTTCTTCTGTCAGCCAGAGCACCCAAATGATTTCTCAGGCACTGAGC

Amino Acid Sequence:

AGSSAAAAAAASGSGGYGPENQGPSGPVAYGPGGPVSSAAAAAAAGSGPGGYGPENQGPSGPGGYGPGGSGSSAAAAAAAASGPGGYGPGSQGPSGPGGSGGYGPGSQGASGPGGPGASAAAAAAAAAASGPGGYGPGSQGPSGPGAYGPGGPGSSAAAAAAAASGPGGYGPGSQGPSGPGVYGPGGPGSSAAAAAAAGSGPGGYGPENQGPSGPGGYGPGGSGSSAAAAAAAASGPGGYGPGSQGPSGPGGSGGYGPGSQGGSGPGASAAAAAAAASGPGGYGPGSQGPSGPGYQGPSGPGAYGPSPSASASVAASVYLRLQPRLEVSSAVSSLVSSGPTNGAAVSGALNSLVSQISASNPGLSGCDALVQALLELVSALVAILSSASIGQVNVSSVSQSTQMISQALS

7. Supplementary References

1. Muller, J., S. Oehler, and B. Muller-Hill, *Repression of lac promoter as a function of distance, phase and quality of an auxiliary lac operator.* J Mol Biol, 1996. **257**(1): p. 21-9.

2. Darwin, K.H. and V.L. Miller, *Type III secretion chaperone-dependent regulation: activation of virulence genes by SicA and InvF in Salmonella typhimurium.* Embo J, 2001. **20**(8): p. 1850-62.

3. Widmaier, D.M., et al., *Engineering the Salmonella type III secretion system to export spider silk monomers.* Mol Syst Biol, 2009. **5**: p. 309.

4. Ayoub, N.A., et al., *Blueprint for a high-performance biomaterial: full-length spider dragline silk genes.* PLoS One, 2007. **2**(6): p. e514.
